# Tandem Thio‐Michael Addition/Remote Lactone Activation of 5‐Hydroxymethylfurfural‐Derived δ‐Lactone‐Fused Cyclopentenones

**DOI:** 10.1002/cssc.202102204

**Published:** 2022-01-18

**Authors:** Rafael F. A. Gomes, Joao M. J. M. Ravasco, Késsia H. S. Andrade, Jaime A. S. Coelho, Rui Moreira, Rafael Oliveira, Fátima Nogueira, Carlos A. M. Afonso

**Affiliations:** ^1^ Research Institute for Medicines (iMed.ULisboa) Faculty of Pharmacy Universidade de Lisboa Av. Prof. Gama Pinto 1649-003 Lisboa Portugal; ^2^ Centro de Química Estrutural, Institute of Molecular Sciences Faculdade de Ciências Universidade de Lisboa Campo Grande 1749-016 Lisboa Portugal; ^3^ Global Health and Tropical Medicine GHTM Instituto de Higiene e Medicina Tropical IHMT Universidade NOVA de Lisboa UNL Rua da Junqueira, 10 1349-008 Lisboa Portugal; ^4^ Institute of Tropical Medicine and International Health Charité – Charité-Universitätsmedizin Berlin Augustenburger Platz 1 (Campus Adress: Südring 2–3) 13353 Berlin Germany

**Keywords:** antimalarial, biomass, cyclopentenones, furans, sustainable chemistry

## Abstract

The creation of structurally diverse chemical entities from fairly simple biorefinery products remains a challenge. In this work 5‐hydroxymethylfurfural (HMF) was identified as a key synthon for preparing highly complex cyclopentenones (CP) via tandem 1,4‐addition/elimination/remote lactone activation to external O‐ and N‐nucleophiles in δ‐lactone‐fused‐CPs hotspots. This scaffold was also reactive enough to be incorporated into model cysteine‐peptides in low concentrations, paving the way to a potential translation generating complexity in the synthesis of small peptides. The new enones also exhibited activity against intraerythrocytic *Plasmodium falciparum* (IC_50_=1.32 μm).

## Graphical abstract

**Building a scaffold**: Bicyclic cyclopentenones, readily available from biomass‐derived 5‐hydroxymethylfurfural (HMF), undergo a tandem Michael‐addition/elimination/methanolysis to obtain new bioactive scaffolds. Integrated in a diversity‐oriented synthesis strategy of HMF, the work focuses on the mechanism of the remote lactone activation and on a broad substrate scope, including biomacromolecules, thus discovering an antimalarial hit compound.

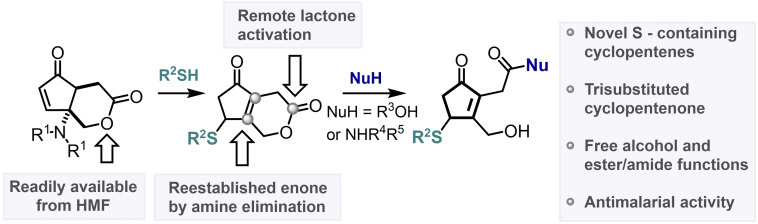

## Introduction

**Figure 1:**
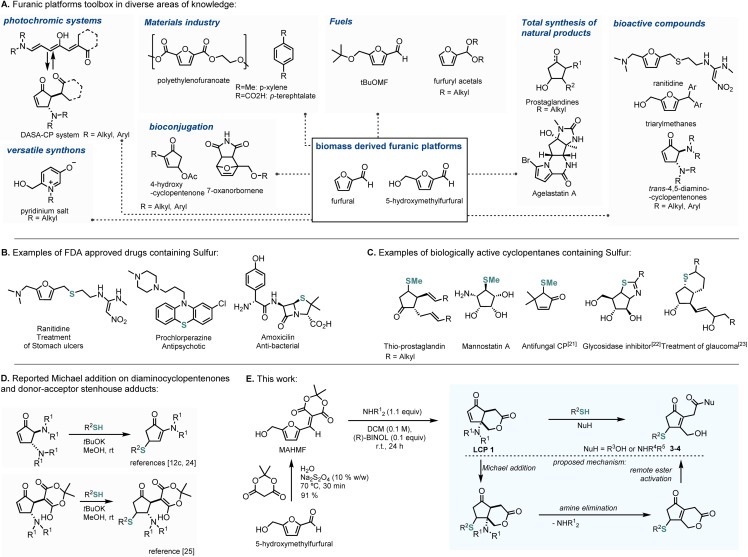
Importance of synthetic diversification of biomass derived furanic platforms. (A) Furanic platforms toolbox in diverse areas of knowledge. (B) Examples of FDA‐approved drugs containing sulfur. (C) Examples of biologically active cyclopentanes containing sulfur. (D) Reported Michael addition on a diaminocyclopentenones and donor–acceptor stenhouse adducts. (E) This work.

The increased awareness concerning sustainability has led to a shift for greener approaches towards fine chemicals from the materials and pharma industries.^[1]^ In this sense, and in line with one of the 12 principles of green chemistry,^[2]^ the use of biorenewable feedstock for the production of diversified compounds is an important topic that has been tackled by several research groups. Amongst the biorenewable feedstocks,^[3]^ furanic platform molecules such as furfural and 5‐hydroxymethylfurfural (HMF) are included in the top 10+4 biobased product opportunities^[4]^ and have been used for the preparation of value‐added chemicals as depicted in Figure 1A (e. g., photochromic systems;^[5]^ materials;^[6]^ fuels;^[7]^ intermediates for total synthesis;^[8]^ bioactive compounds including FDA‐approved ranitidine;^[9]^ drug delivery;^[10]^ bioconjugation systems^[11]^). Additionally, the reactivity of furanic platforms has been harnessed to design complex structures such as 7‐oxanorbornenes, triarylmethanes, pyridinium salts, and substituted cyclopentenones (CP).

A popular strategy to access these latter CPs has been through the Lewis acid‐promoted condensation of furan derivatives with secondary amines.^[12]^ In 2014, Read de Alaniz and co‐workers reported a catalyst‐free methodology to generate donor–acceptor Stenhouse adducts (DASA) through condensation of amines and activated furfural, which upon irradiation undergo cyclization to the corresponding CP.^[13]^ More recently, activated HMF prepared by Knoevenagel condensation with Meldrum's acid^[14]^ was reported to react with secondary amines under mild conditions to afford hydrolytically stable δ‐lactone‐fused CP (LCP, Figure 1D).^[15]^

Transversal to most value‐added products obtained from furans is the high content of oxygen and nitrogen. While not native to these renewable raw sources, sulfur is a key constituent of over 20 % FDA‐approved drugs (Figure 1B),[^16^, ^17^] mostly in the form of sulfonamide, sulfone, or sulfide, and is responsible for significant favorable S–aromatic and S–O/N interactions in many targets.^[18]^ Indeed, sulfur‐containing scaffolds are transversal in medicinal chemistry, and it is one of the most abundant heteroatoms in FDA‐approved drugs. Amongst these sulfur‐containing scaffolds, several cyclopentanes have relevant biological activities (Figure 1C). For instance, thio‐prostanglandines have shown relevant bronchodilator properties,^[19]^ while mannostatin A, a sulfur‐containing aminocyclopentitol, has been described to possess α‐mannosidase inhibitory activity.^[20]^ Miller and co‐workers reported sulfur‐containing cyclopentenones (CP) with remarkable antifungal activity.^[21]^ Some sulfur‐containing cyclopentanes have been patented both as glycosidase inhibitors^[22]^ and for the treatment of glaucoma.^[23]^

To increase the chemical space enclosed by furan‐derived products, the CP‐enone system has been exploited to incorporate sulfur via Michael addition. For instance thiophenol can undergo 1,4‐addition with 4,5‐diaminoCP (DCP) in the presence of base, in which occurs a subsequent elimination of the amine at C4 position (Figure 1D).[^12c^, ^24^] Barner‐Kowollik and co‐workers expanded the scope of this methodology to the DASA‐CP system, which in contrast to the aforementioned, occurs without C4 amine elimination (Figure 1D).^[25]^

Despite the structural similarity to DASA‐CP systems, LCPs undergo elimination of the amine upon Michael addition of thiophenol,^[15]^ in the likelihood to DCP systems.

Our approach was based on the mechanistic hypothesis that the reestablishment of the enone could lead to a consequent activation of the previously hydrolytically stable lactone to allow subsequent opening with an external nucleophile. This offers a unique opportunity to access new biomass‐derived CP molecular skeletons bearing multifunctionality and potentially interesting biological activity (Figure 1E). In such regard, herein we report the study and reactivity of LCP with thiols as a versatile tool for the generation of functionally rich biomass‐derived scaffolds bearing an enone, a sulfide, a free alcohol, and an ester/amide functions (Figure 1E) and evaluate the biological activity of these new complex CPs.

## Results and Discussion

**Figure 2:**
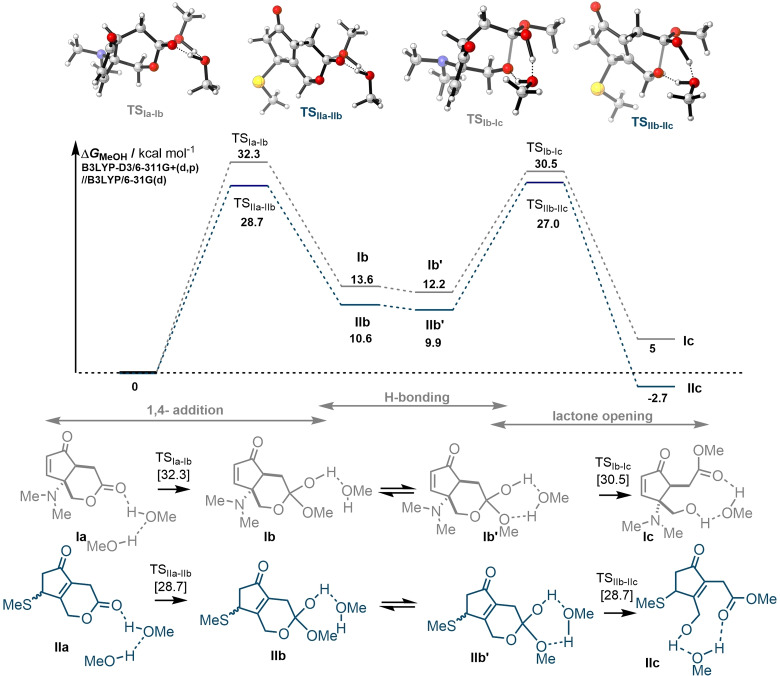
Free‐energy profiles for the methanolysis of lactones **Ia** and **IIa**.

Density functional theory (DFT) calculations were performed at B3LYP‐D3/6‐311+G(d,p)/SMD(MeOH) level of theory for the methanolysis of **1** and **2** (Figure 2). Calculations indicated that the formation of the methanolysis product from **1** is thermodynamically disfavored (Δ*G*=5.0 kcal mol^−1^), whereas methanolysis product from the thiol addition product **2** is favored (Δ*G*=−2.7 kcal mol^−1^), suggesting that re‐establishment of the enone post‐1,4‐Michael addition might indeed affect the lactone stability.

This is in accordance with experimental results where LCP **1** was resistant to methanolysis under basic (NaOMe in refluxing MeOH) and acidic [*para*‐toluenesulfonic acid (pTSA) in refluxing MeOH] conditions.

We continued by inquiring whether the 1,4‐thiol addition to LCP would occur using mild reaction conditions and how it would impact the stability of the lactone. In this line, a model reaction using thiophenol and LCP **1** was studied in methanol at room temperature. In the absence of base, the reaction proceeded smoothly with 80 % conversion after 5 h (Table 1, entry 1). Interestingly, the lactone **2 a** was obtained as the major product, resulting from the elimination of the amine. However, full methanolysis to **3 a** was observed after 12 h, which indicated an increased susceptibility of the lactone to hydrolysis, probably as a consequence to the enone shift (Table 1, entry 2). The use of sodium methoxide or potassium *tert*‐butoxide resulted in the selective formation of **3 a**, affording 60 and 70 % yield after 5 h (Table 1, entries 3 and 4 respectively). Additionally, whereas previous findings showed that thiol additions to DCP could not be performed in the presence of sodium methoxide due to the competitive methoxide addition to the olefin,^[24]^ this was not observed in this case (LCP). Both potassium carbonate and sodium hydroxide resulted in a marked decrease in the formation of **3 a** (Table 1, entries 5 and 6).

**Table 1:** Reaction optimization.^[a]^

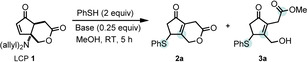				
Entry	Base	Yield^[b]^ [%]		Conv.^[b]^
		**2 a**	**3 a**	[%]
1	–	70	10	80
2^[c]^	–	0	80	100
3	KO*t*Bu	0	70	100
4	NaOMe	0	60	100
5	K_2_CO_3_	0	20	100
6	NaOH	0	40	100
7^[d]^	–	42	0	57

[a] Reaction conditions: **1** (20 mg, 0.8 mmol), PhSH (18 mg, 2.0 equiv.), MeOH (0.8 mL), and base (0.25 equiv.). [b] Yield calculated by ^1^H NMR spectroscopy using 1,3,5‐trimethoxybenzene as internal standard. [c] Reaction time is 12 h. [d] Reaction performed with 1 equiv. of PhSH.

Despite the improvement of the reaction rates when employing base, we decided to proceed with the base‐free method due to the reduction of work‐up steps (the base‐promoted reaction requires an extraction prior to chromatography) and the possibility to use base‐sensitive moieties in future works. Reducing the load of thiol reduced the yield of **2 a** to 42 % (Table 1, entry 7).

**Scheme 1:**
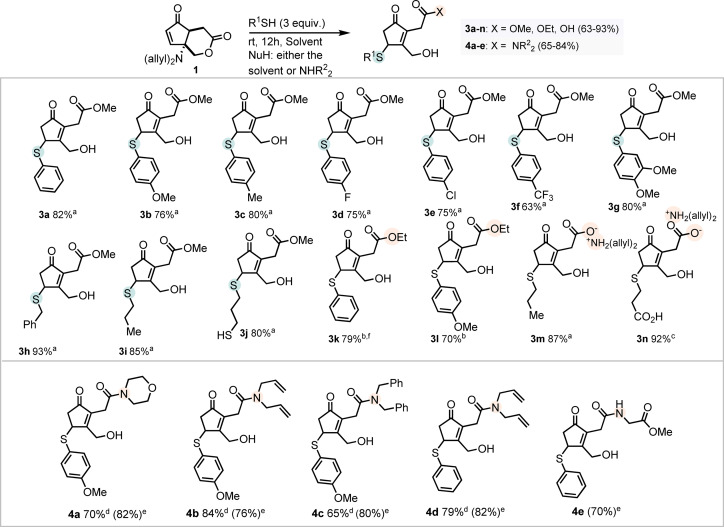
Reaction conditions: **1** (100 mg, 4.0 mmol), RSH (3 equiv., 12 mmol), and solvent (4 mL). [a] Reaction performed in MeOH. [b] Reaction performed in EtOH. [c] Reaction performed in water. [d] Reaction performed in acetonitrile using 5 equiv. of NHR^2^_2_. [e] Reaction performed in *i*PrOH using 5 equiv. of NHR^2^_2_. [f] Reaction performed on 1 g scale.

Next, we studied the reactivity of **1** with different thiol nucleophiles, under the aforementioned conditions (Scheme 1). A series of aliphatic, benzylic, and electronically distinct thiophenols were used, achieving moderate‐to‐excellent yields (63–93 %), although in some cases the products can be contaminated with traces of closed lactone as observed by ^1^H NMR spectroscopy. Both electron‐rich as well as electron‐poor thiophenols afforded moderate‐to‐good yields (**3 a**–**g**; 63–82 %), with a slight drop in yield when employing electron‐poor thiols (**3 f**, 63 % and **3 e**, 75 %). The use of a benzylic nucleophile resulted in excellent yield (**3 h**; 93 %), while alkyl thiols did not significantly differ from the aromatic analogs (**3 i** and **3 j**, 85 and 80 %, respectively). Notably, the use of propanedithiol showed no traces of either dimerization or dithiolane formation, providing **3 j** as a single product in 80 % yield. The reaction was also performed in ethanol using thiophenol and 4‐methoxythiophenol, affording **3 k** and **3 l** in 79 and 70 % yield. Importantly, the reaction is easily scalable, with **3 k** efficiently produced on a gram scale (Scheme 1). The reaction in water occurred smoothly using propanethiol and a carboxylic acid thiol, affording the corresponding salt with diallylamine **3 m** and **3 n** (Scheme 1). To further explore the versatility of the remote lactone activation, we turned our attention to the formation of amides. Thus, we performed the 1,4‐addition followed by amine capture of intermediate **2** in a one‐pot fashion by using non‐nucleophilic solvents and an external amine. As model reaction we selected 4‐methoxythiophenol, **1**, and morpholine, using acetonitrile as solvent. Gratifyingly, after 12 h the sole observable product was the result of the thiol addition with incorporation of morpholine **4 a** (Scheme 1). The amidation occurred in the presence of morpholine, piperidine, diallylamine, and dibenzylamine (**4 a**–**e**) in good yields both in acetonitrile and isopropanol (Scheme 1).

Attempts to incorporate amino acid derivative glycine methyl ester in acetonitrile were not complete, and formation of non‐identifiable side products occurred. However, when performed in isopropanol, the reaction occurred smoothly and the desired product **4 e** was isolated in 70 % yield.

**Scheme 2:**
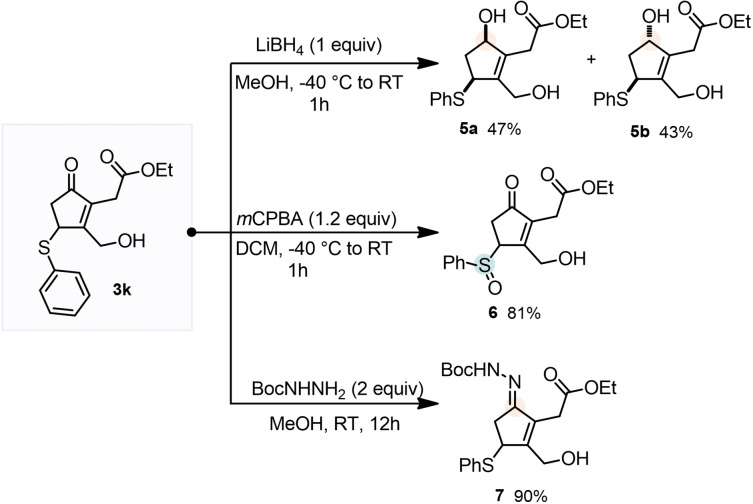
Structural diversification of **3**.

Complex CPs **3** can be target of further diversification, which is of particular importance when pursuing possible hits for medicinal chemistry purposes (Scheme 2). Luche reduction of **3 k** afforded two diastereomers **5 a** and **5 b**, which were easily separated by chromatography in a roughly 1 : 1 ratio with overall 90 % yield. Oxidation to the sulfoxide promoted by *meta*‐chloroperoxybenzoic acid (*m*CPBA) afforded **6** in 81 % yield. Reaction with Boc‐hydrazine afforded the hydrazone **7** in excellent yield (90 %), without traces of aza‐Michael product even using of hydrazine excess (2 equiv.).

**Figure 3:**
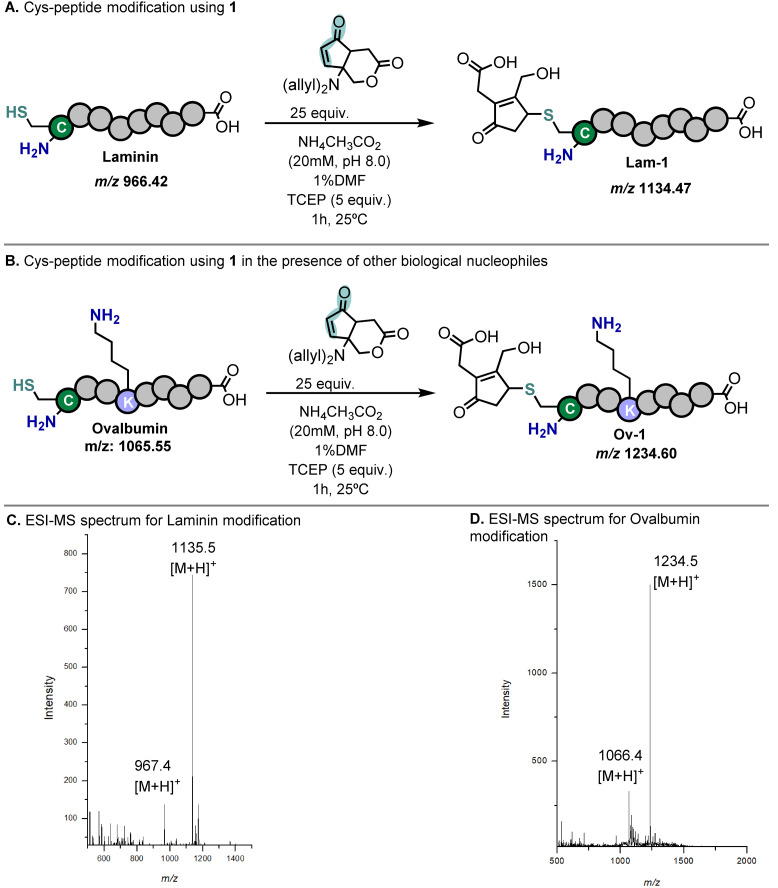
Modification of peptide structures using **1** (25 equiv.) in ammonium acetate buffer (20 mm, pH 8.0) at 25 °C for 1 h. (A) Modification of a laminin fragment with **1**. (B) Modification of an ovalbumin fragment with **1**. (C) ESI‐MS spectrum for laminin modification. (D) ESI‐MS spectrum for ovalbumin modification.

The possibility of performing this reaction in aqueous environment prompted us to inquire whether this scaffold could be used to quickly react with more complex structures, namely under bioconjugation conditions as described recently for other cyclopentenones.^[11d]^ For this purpose, a laminin fragment peptide, a non‐internalizing peptide containing a single cysteine at the N‐terminal position, was used as model nucleophile. The construct **Lam‐1** (*m*/*z* 1135) was observed by electrospray ionization mass spectrometry (ESI‐MS; Figure 3) upon incubation with **1** (25 equiv.) in ammonium acetate buffer (20 mm, pH 8.0) at 25 °C for 1 h (Figure 3A). Due to the highly diluted environment, ring‐opening hydrolysis of the 1,4‐lactone intermediate promptly occurred, and the ring‐closed lactone conjugate was never observed. Despite being less reactive than gold‐standard bioconjugation tools such as maleimides and other activated Michael acceptors,^[26]^ **1** is capable of delivering clean modifications in mild and fast conditions. Moreover, the 1,4‐product showed excellent stability at pH 8 and in the presence of a large excess of mercaptoethanol for up to 24 h. Interestingly, this contrasts with the reported stability of other cyclopentenones in peptides,^[11d]^ highlighting the important protective effect of the alkyl substitution in this enone product. Modification of an ovalbumin peptide containing both an internal lysine and a terminal cysteine resulted in single cysteine modification, with no aza‐Michael cross‐reactivity, even in the presence of a large excess of **1** (25–100 equiv.; Figure 3B). While rapid ring opening under diluted aqueous media may impair the introduction of an external nucleophile, the fast kinetics highlight its potential usefulness as an easily introducible hotspot for generating complexity in small peptides on a larger scale employing compatible non‐aqueous solvents (e. g., DMF, *i*PrOH).

**Figure 4:**
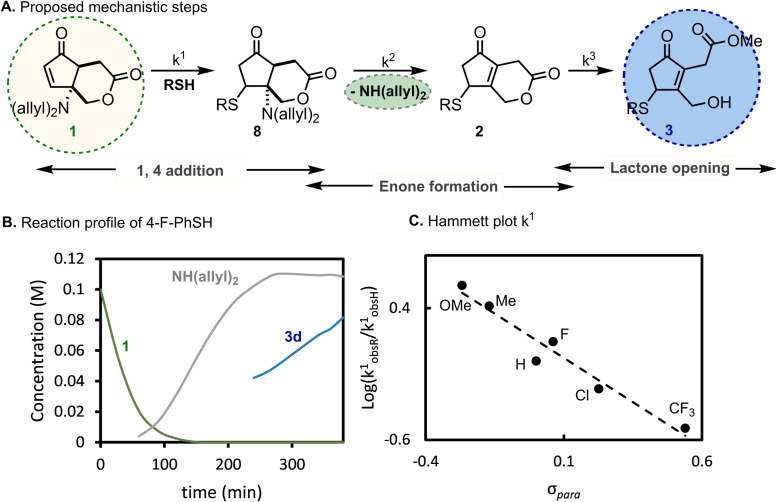
(A) Proposed reaction mechanism. (B) Reaction profile of 4‐F‐PhSH. (C) Hammett plot indicating strong influence of the electronic character of the thiol on rates of the Michael addition. k^1^_obsR_=observed rate constant for the first elementary step of the corresponding R substituent of thiophenol; k^1^_obsH_=observed rate constant for the first elementary step of thiophenol.

To grasp further knowledge on the dynamics and proposed mechanism of this 1,4‐addition‐driven remote lactone activation (Figure 4A), we monitored the reaction of **1** with 4‐F‐thiophenol by ^1^H NMR spectroscopy. The reaction profile shows rapid monoexponential consumption of **1** accompanied by the formation of an intermediate, which can be postulated to be structure **8**, as elimination of diallylamine does not immediately proceed to 1,4‐addition. Unlike previous reports of Michael additions to CPs,^[27]^ full consumption of the starting material is possible due to prohibition of the retro‐Michael reaction driven by irreversible elimination of diallylamine to afford intermediate enone **2**. Lactone opening, which is unfavored in **1**, is now more favored by the enone‐shift in **2** and proceeds to afford **3** (Figure 4B). The effect of the thiol substituent was evaluated by performing the reaction between **1** and an excess of different *para*‐substituted aryl thiols.

Quantitative ^1^H NMR spectroscopic kinetics were traced for the different thiols, and a correlation was observed between the rate constant for the first elementary step (*k*_1_) and the electronegativity of the substituent. Moreover, the Hammett plot of log(*k* ^1^_obs*R*_/*k* ^1^_obsH_) vs. *σ*_para_ of the thiol substituents was linear with a *ρ* value of −4.49, similar to previous findings in aza‐Michael additions (Figure 4C).^[28]^

**Scheme 3:**
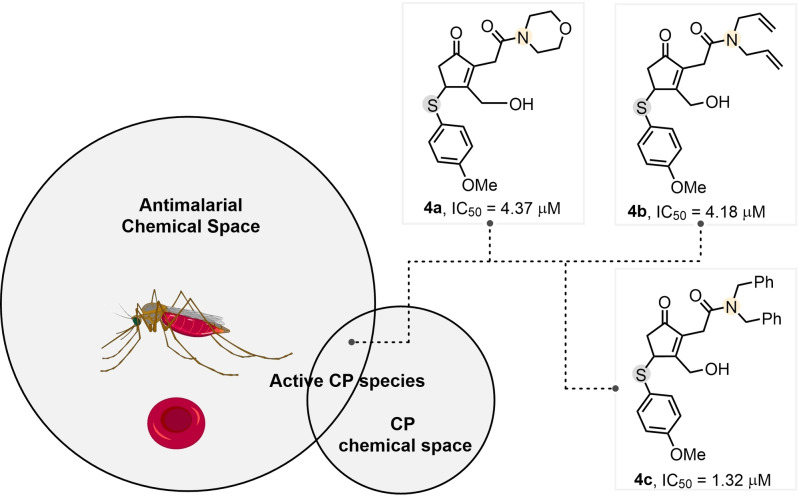
Modification of Antimalarial activities evaluation. IC_50_ against *Plasmodium falciparum* (3D7‐GFP).

The ability of cyclic enones to react as efficient electrophiles^[29]^ led us to evaluate the biological activity of compounds **3**–**4**. When tested against a panel of cancer cell lines (HT‐29, MCF‐7, NCI‐H460), none of the CPs displayed cytotoxicity. Based on precedented reports that Michael acceptors are active against the malaria parasites, both *in* *vitro* and *in* *vivo*,^[30]^ through inhibition of cysteine proteases, we tested selected enones for antiplasmodial activity. When screened for *in* *vitro* activity against intraerythrocytic stages of *Plasmodium falciparum* (3D7‐GFP), amides **4 a**–**c** presented antimalarial activity in the low μm range (Scheme 3). The half maximal inhibitory concentration activity is summarized in Scheme 3. The corresponding esters and acids **3** were not active.

Importantly, the CPs were not toxic in healthy cell line HEK 293, exhibiting IC_50_>50 μm (see the Supporting Information), suggesting that compounds **4** have potential for further optimization as antiplasmodial agents.

## Conclusions

We have developed a methodology to incorporate sulfur in complex cyclopentenones (CPs) obtained from biorefinery furanic platforms. This occurs via Michael addition leading to a consequent remote lactone activation. Modification of peptides using δ‐lactone‐fused CP (LCP) was successfully achieved and occurs selectively in cysteine residues, which can be employed for the design of small peptide‐CP scaffolds, highlighting its potential as an easily introducible hotspot to generate complexity in the synthesis of small peptides on a larger scale. This ligation is very stable, and the CP handle can potentially be used to anchor a variety of payloads through modification of the added functionalities (enone, allylic alcohol, carboxylic acid) or serve as a glycomimetic appendage. Finally, we observed that the new complex thio‐CP, in particular the amide derivatives, exhibited promising antimalarial activity with IC_50_ of 1.32 μm against intraerythrocytic *Plasmodium falciparum* (3D7‐GFP). These results highlight the importance of structural diversification for the discovery of new scaffolds for drug discovery. Further studies on lead optimization and target identification of CP derivatives are ongoing.

## Conflict of interest

The authors declare no conflict of interest.

## Supporting information

As a service to our authors and readers, this journal provides supporting information supplied by the authors. Such materials are peer reviewed and may be re‐organized for online delivery, but are not copy‐edited or typeset. Technical support issues arising from supporting information (other than missing files) should be addressed to the authors.

Supporting Information
