# Supplementary material for: Tandem Thio‐Michael Addition/Remote Lactone Activation of 5‐Hydroxymethylfurfural‐Derived δ‐Lactone‐Fused Cyclopentenones
Source: ChemSusChem. 2022 Jan 18;15(13):e202102204. doi: 10.1002/cssc.202102204 (PMC9401029; doi:10.1002/cssc.202102204)
Supplement: Supplementary file 1 — Supporting Information [file CSSC-15-0-s001.pdf]

ChemSusChem

Supporting Information

Tandem Thio-Michael Addition/Remote Lactone Activation of 5-Hydroxymethylfurfural-Derived δ-Lactone-Fused Cyclopentenones

Rafael F. A. Gomes,* Joao M. J. M. Ravasco, Késsia H. S. Andrade, Jaime A. S. Coelho, Rui Moreira, Rafael Oliveira, Fátima Nogueira, and Carlos A. M. Afonso*This publication is part of a collection of invited contributions focusing on "Green Conversion of HMF". Please visit to view all contributions.© 2022 The Authors. ChemSusChem published by Wiley-VCH GmbH. This is an open access article under the terms of the Creative Commons Attribution License, which permits use, distribution and reproduction in any medium, provided the original work is properly cited.

Supporting Information

Tandem thio-Michael addition/remote lactone activation of 5-hydroxymethylfurfural derived δ-lactone-fused cyclopentenones

Rafael F. A. Gomes*,[a] Joao M. J. M. Ravasco,[a] Késsia H. S. Andrade,[a] Jaime A. S. Coelho,[b] Rui Moreira,[a] Rafael Oliveira,[c],[d] Fátima Nogueira,[c] Carlos A. M. Afonso*[a]

Table of Contents

General Information 3

General Procedure for the synthesis of LCP-thioadducts in nucleophilic solvents 3

General Procedure for the synthesis of LCP-thioadducts in nucleophilic solvents 8

Reduction of thio-adduct CP: 10

Oxidation of thio-adduct CP to ethyl 2-(2-(hydroxymethyl)-5-oxo-3-(phenylsulfinyl)cyclopent-1-en-1-yl)acetate 6 11

Hydrazone formation of thio-adduct CP to tert-butyl (E)-2-(2-(2-ethoxy-2-oxoethyl)-3-(hydroxymethyl)-4-(phenylthio)cyclopent-2-en-1-ylidene)hydrazine-1-carboxylate 7 12

NMR studies: 13

Peptide Assays 14

Computational studies 17

Biological studies 25

Copies of NMR spectra 28

References 69

General Information

All solvents were distilled prior to use. All reagents were used as received from commercial suppliers, unless otherwise stated. 5-Hydroxymethylfurfural[1] and δ-Lactone-fused Cyclopentenones (LCP **1**)[2] was prepared according to our previously reported procedure. Reaction progress was monitored by thin-layer chromatography (TLC) performed on aluminum plates coated with silica gel F254 with 0.2 mm thickness. ^{1}H and ^{13}C NMR spectra were acquired on Bruker MX300 spectrometer.

General Procedure for the synthesis of LCP-thioadducts in nucleophilic solvents

To a solution of LCP **1** (100 mg, 0.40 mmol) in MeOH (4 mL) was added the corresponding thiol (3 equiv, 1.2 mmol) and the mixture was allowed to stir for 12 h. The solvent was evaporated under reduced pressure and the crude mixture was purified by column chromatography.

Methyl 2-(2-(hydroxymethyl)-5-oxo-3-(phenylthio)cyclopent-1-en-1-yl)acetate **3a**

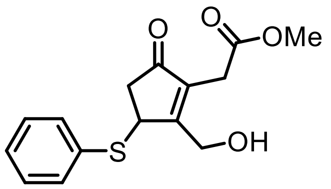

The titled compound was prepared according to general procedure. The crude mixture was purified by flash chromatography using hexane:ethyl acetate (9:1 to 2:8) affording 100 mg (82% yield) of pure product as a yellow oil.

^{1}H NMR (300 MHz, $CDCl_3$) δ 7.55 – 7.29 (m, 5H), 5.02 – 4.56 (m, 2H), 4.35 (m, 1H), 3.69 (s, 3H), 3.35 (s, 2H), 2.91 (dd, J = 19.2, 6.7 Hz, 1H), 2.56 (dd, J = 19.2, 1.8 Hz, 1H).**^{13}C NMR (75 MHz, $CDCl_3$)** δ 208.4, 163.2, 139.4, 136.5, 135.2, 129.5, 128.3, 127.26, 117.3, 64.6, 64.1, 55.3, 53.6. **HRMS (ESI-MS)** *m/z* calcd for $C_{15}H_{17}O_4S$ $[M + H]^+$ 293.08475, found 293.08395.

Methyl 2-(2-(hydroxymethyl)-3-((4-methoxyphenyl)thio)-5-oxocyclopent-1-en-1-yl)**acetate 3b**

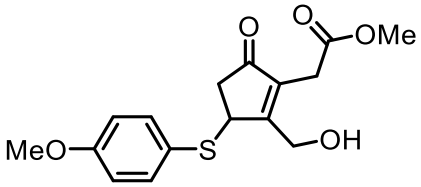

The titled compound was prepared according to general procedure. The crude mixture was purified by flash chromatography using hexane:ethyl acetate (9:1 to 2:8) affording 99 mg (76% yield) of pure product as a yellow oil.

^{1}H NMR (300 MHz, $CDCl_3$) δ 7.24 (d, J = 8.9 Hz, 2H), 6.74 (d, J = 8.8 Hz, 2H), 4.75 (d, J = 16.1 Hz, 1H), 4.63 (d, J = 16.1 Hz, 1H), 4.20 – 4.07 (m, 1H), 3.71 (s, 3H), 3.60 (s, 3H), 3.24 (s, 2H), 2.76 (dd, J = 19.2, 6.8 Hz, 1H), 2.44 (dd, J = 19.2, 1.8 Hz, 1H).**^{13}C NMR (75 MHz, $CDCl_3$)** δ 204.6, 171.9, 161.8, 160.5, 137.0, 134.9, 120.6, 114.7, 69.1, 60.5, 55.3, 46.7, 43.4, 28.1. **HRMS (ESI-MS)** *m/z* calcd for $C_{16}H_{19}O_5S$ $[M + H]^+$ 323.09532, found 323.09497

Methyl 2-(3-((4-methylphenyl)thio)-2-(hydroxymethyl)-5-oxocyclopent-1-en-1-yl)acetate **3c**

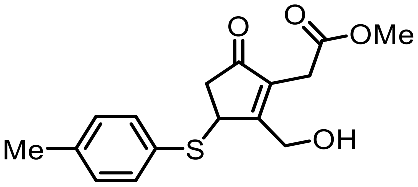

The titled compound was prepared according to general procedure. The crude mixture was purified by flash chromatography using hexane:ethyl acetate (9:1 to 2:8) affording 99 mg (80% yield) of pure product as a yellow oil.

^{1}H NMR (300 MHz, $CDCl_3$) δ 7.27 (d, J = 8.1 Hz, 2H), 7.20 – 6.96 (m, 2H), 4.81 (d, J = 16.0 Hz, 1H), 4.69 (d, J = 16.1 Hz, 1H), 4.30 (m, 1H), 3.69 (s, 3H), 3.34 (d, J = 1.0 Hz, 2H), 2.87 (dd, J = 19.2, 6.7 Hz, 1H), 2.53 (dd, J = 19.2, 1.8 Hz, 1H), 2.33 (s, 3H).**^{13}C NMR (75 MHz, $CDCl_3$)** δ 204.6, 171.9, 171.8, 139.1, 135.2, 134.3, 130.1, 127.6, 60.6, 52.6, 46.6, 42.3, 28.3, 21.3. **HRMS (ESI-MS)** *m/z* calcd for $C_{16}H_{19}O_4S$ $[M + H]^+$ 307.10040, found 307.09982.

Methyl 2-(3-((4-fluorophenyl)thio)-2-(hydroxymethyl)-5-oxocyclopent-1-en-1-yl)**acetate 3d**

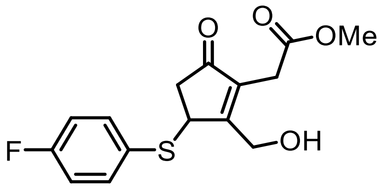

The titled compound was prepared according to general procedure. The crude mixture was purified by flash chromatography using hexane:ethyl acetate (9:1 to 2:8) affording 94 mg (75% yield) of pure product as a yellow oil.

^{1}H NMR (300 MHz, $CDCl_3$) δ 7.40 – 7.35 (m, 2H), 7.02 – 6.96 (m, 2H), 4.82 (d, J = 16.1 Hz, 1H), 4.69 (d, J = 16.3 Hz, 1H), 4.26 (m, 1H), 3.69 (s, 3H), 3.34 (s, 2H), 2.87 (dd, J = 19.3, 6.8 Hz, 1H), 2.50 (dd, J = 19.2, 1.7 Hz, 1H). **^{13}C NMR (75 MHz, $CDCl_3$)** δ 204.3, 171.9, 171.7, 165.0, 136.9, 136.8, 135.4, 125.9, 116.7, 116.4, 60.6, 52.7, 46.7, 42.1, 28.2. **HRMS (ESI-MS)** *m/z* calcd for $C_{15}H_{16}FO_4S$ $[M + H]^+$ 311.07533, found 311.07471

Methyl 2-(3-((4-chlorophenyl)thio)-2-(hydroxymethyl)-5-oxocyclopent-1-en-1-yl)acetate **3e**

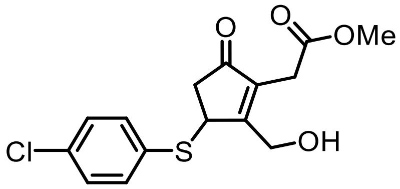

The titled compound was prepared according to general procedure. The crude mixture was purified by flash chromatography using hexane:ethyl acetate (9:1 to 2:8) affording 99 mg (75% yield) of pure product as a yellow oil.

^{1}H NMR (300 MHz, $CDCl_3$) δ 7.74 – 6.97 (m, 4H), 4.76 (d, J = 16.0 Hz, 1H), 4.62 (d, J = 15.9 Hz, 1H), 4.28 (m, 1H), 3.65 (s, 3H), 3.82 (s, 2H), 2.86 (dd, J = 19.3, 6.7 Hz, 1H), 2.46 (dd, J = 19.3, 1.5 Hz, 1H).**^{13}C NMR (75 MHz, $CDCl_3$)** δ 204.3, 171.8, 171.6, 135.6, 135.0, 129.9, 129.5, 60.41,

52.7, 46.4, 42.3, 28.3. **HRMS (ESI-MS)** *m/z* calcd for $C_{15}H_{16}ClO_4S$ $[M + H]^+$ 327.04523 and 329.04228, found 327.04462 and 329.04123.

Methyl 2-(3-((4-trifluoromethylphenyl)thio)-2-(hydroxymethyl)-5-oxocyclopent-1-en-1-yl)acetate **3f**

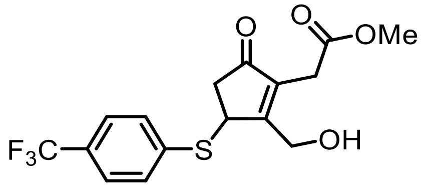

The titled compound was prepared according to general procedure. The crude mixture was purified by flash chromatography using hexane:ethyl acetate (9:1 to 2:8) affording 92 mg (63% yield) of pure product as a yellow oil.

^{1}H NMR (300 MHz, CDCl$_3$) δ 7.55 (d, J = 8.4 Hz, 2H), 7.47 (d, J = 8.3 Hz, 2H), 4.77 (d, J = 15.8 Hz, 1H), 4.64 (d, J = 15.8 Hz, 1H), 4.52 – 4.37 (m, 1H), 3.70 (s, 3H), 3.41 (s, 2H), 3.00 (dd, J = 19.2, 6.7 Hz, 1H), 2.56 (dd, J = 19.2, 1.9 Hz, 1H).**^{13}C NMR (75 MHz, CDCl$_3$)** δ 203.9, 171.7, 170.9, 138.2, 136.1, 131.4, 129.7, 126.2, 126.2, 126.2, 126.1, 125.8, 60.3, 52.7, 45.8, 42.9, 28.3. **HRMS (ESI-MS)** *m/z* calcd for $C_{16}H_{16}F_3O_4S$ $[M + H]^+$ 361.07214, found 361.07159

Methyl 2-(3-((3,4-dimethoxyphenyl)thio)-2-(hydroxymethyl)-5-oxocyclopent-1-en-1-yl)acetate **3g**

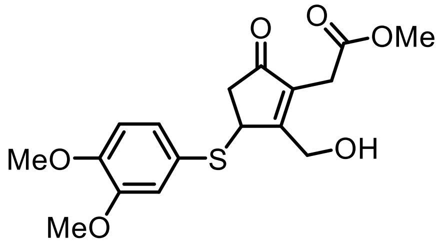

The titled compound was prepared according to general procedure. The crude mixture was purified by flash chromatography using hexane:ethyl acetate (9:1 to 2:8) affording 114 mg (80% yield) of pure product as a yellow oil.

^{1}H NMR (300 MHz, CDCl$_3$). δ 6.97 (dd, J = 8.3, 1.9 Hz, 1H), 6.87 (s, 1H), 6.76 (dd, J = 8.3, 1.5 Hz, 1H), 4.81 (d, J = 16.1 Hz, 1H), 4.68 (d, J = 16.1 Hz, 1H), 4.22 (m, 1H), 3.84 (s, 3H), 3.83 (s, 3H), 3.65 (s, 3H), 3.31 (s, 2H), 2.96 – 2.74 (m, 1H), 2.54 (dd, J = 19.2, 1.7 Hz, 1H). **^{13}C NMR (75 MHz, CDCl$_3$)** δ 204.6, 172.0, 171.9, 150.1, 149.1, 135.0, 128.4, 121.3, 117.8, 111.5, 60.6, 56.1, 55.9, 52.6, 46.9, 42.1, 28.2. **HRMS (ESI-MS)** *m/z* calcd for $C_{17}H_{21}O_6S$ $[M + H]^+$ 353.10588, found 353.10541.

Methyl 2-(3-(benzylthio)-2-(hydroxymethyl)-5-oxocyclopent-1-en-1-yl)acetate **3h**

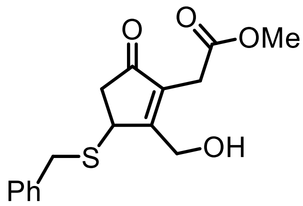

The titled compound was prepared according to general procedure. The crude mixture was purified by flash chromatography using hexane:ethyl acetate (9:1 to 2:8) affording 115 mg (93% yield) of pure product as a yellow oil.

^{1}H NMR (300 MHz, $CDCl_3$) δ 7.47 – 7.13 (m, 5H), 4.56 (d, J = 16.0 Hz, 1H), 4.47 (d, J = 16.0 Hz, 1H), 3.93 (m, 1H), 3.69 (s, 2H), 3.65 (s, 3H), 3.33 (s, 2H), 2.82 (dd, J = 19.3, 6.7 Hz, 1H), 2.47 (dd, J = 19.3, 1.8 Hz, 1H). **^{13}C NMR (75 MHz, $CDCl_3$)** δ 204.9, 172.2, 171.6, 137.6, 135.4, 129.0, 128.9, 127.6, 60.3, 52.6, 43.2, 43.1, 34.7, 28.3. **HRMS (ESI-MS)** *m/z* calcd for $C_{16}H_{19}O_4S$ $[M + H]^+$ 307.10040, found 307.10007.

Methyl 2-(2-(hydroxymethyl)-5-oxo-3-(propylthio)cyclopent-1-en-1-yl)acetate **3i**

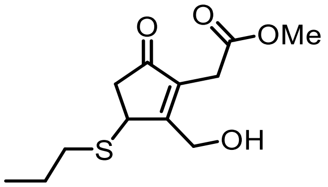

The titled compound was prepared according to general procedure. The crude mixture was purified by flash chromatography using hexane:ethyl acetate (9:1 to 2:8) affording 89 mg (85% yield) of pure product as a yellow oil.

^{1}H NMR (300 MHz, $CDCl_3$) δ 4.83 – 4.47 (m, 2H), 4.17 – 3.90 (m, 1H), 3.69 (s, 3H), 3.41 (s, 2H), 2.96 (dd, J = 19.3, 6.8 Hz, 1H), 2.55 (dd, J = 19.3, 1.8 Hz, 1H), 2.43 (td, J = 7.3, 1.1 Hz, 2H), 1.61 (dt, J = 14.6, 7.6 Hz, 2H), 0.98 (t, J = 7.3 Hz, 3H).**^{13}C NMR (75 MHz, $CDCl_3$)** δ 204.9, 172.5, 171.7, 134.9, 60.5, 52.6, 43.4, 43.0, 31.6, 28.3, 22.9, 13.7. **HRMS (ESI-MS)** *m/z* calcd for $C_{12}H_{19}O_4S$ $[M + H]^+$ 259.10040, found 259.09964.

Methyl 2-(2-(hydroxymethyl)-3-((3-mercaptopropyl)thio)-5-oxocyclopent-1-en-1-yl)acetate **3j**

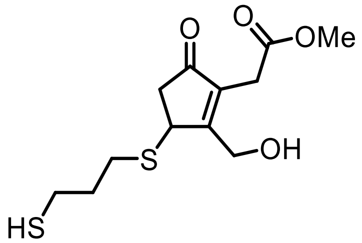

The titled compound was prepared according to general procedure. The crude mixture was purified by flash chromatography using hexane:ethyl acetate (9:1 to 2:8) affording 93 mg (80% yield) of pure product as a yellow oil.

^{1}H NMR (300 MHz, $CDCl_3$) δ 4.82 – 4.51 (m, 2H), 4.17 – 3.97 (m, 1H), 3.69 (s, 3H), 3.41 (s, 2H), 2.97 (dd, J = 19.2, 6.8 Hz, 3H), 2.73 – 2.43 (m, 6H).**^{13}C NMR (75 MHz, $CDCl_3$)** δ 204.7, 172.0, 171.5, 135.2, 60.2, 52.5, 43.2, 42.9, 33.1, 28.2, 27.5, 23.4. **HRMS (ESI-MS)** *m/z* calcd for $C_{12}H_{19}O_4S_2$ $[M - H]^-$ 289.05737, found 289.05777.

Ethyl 2-(2-(hydroxymethyl)-3-(phenylthio)-5-oxocyclopent-1-en-1-yl)acetate **3k**

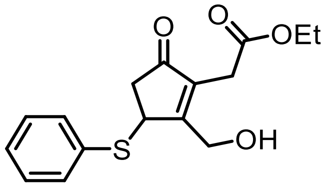

The titled compound was prepared according to general procedure using ethanol as solvent. The crude mixture was purified by flash chromatography using hexane:ethyl acetate (9:1 to 2:8) affording 97 mg (79% yield) of pure product as a yellow oil.

^{1}H NMR (300 MHz, $CDCl_3$) δ 7.41 – 7.33 (m, 2H), 7.32 – 7.27 (m, 3H), 4.79 (d, J = 15.9 Hz, 1H), 4.67 (d, J = 15.9 Hz, 1H), 4.36 (m, 1H), 4.13 (q, J = 7.1 Hz, 2H), 3.33 (s, 2H), 2.90 (dd, J = 19.2, 6.7 Hz, 1H), 2.55 (dd, J = 19.2, 1.8 Hz, 1H), 1.25 (t, J = 7.2 Hz, 3H). **^{13}C NMR (75 MHz, $CDCl_3$)** δ 204.5, 171.7, 171.4, 135.5, 133.5, 131.9, 129.3, 128.5, 61.7, 60.5, 46.4, 42.7, 28.5, 14.2. **HRMS (ESI-MS)** *m/z* calcd for compound $C_{16}H_{19}O_4S$ [M + H]$^+$ 307.09986, found 307.09988.

Ethyl 2-(2-(hydroxymethyl)-3-((4-methoxyphenyl)thio)-5-oxocyclopent-1-en-1-yl)acetate **3l**

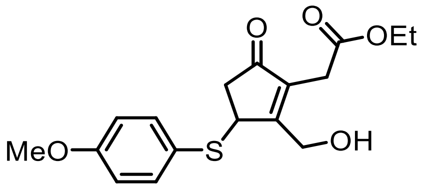

The titled compound was prepared according to general procedure using ethanol as solvent. The crude mixture was purified by flash chromatography using hexane:ethyl acetate (9:1 to 2:8) affording 95 mg (70% yield) of pure product as a yellow oil.

^{1}H NMR (300 MHz, $CDCl_3$) δ 7.24 (d, J = 8.8 Hz, 2H), 6.74 (d, J = 8.8 Hz, 2H), 4.75 (d, J = 16.0 Hz, 1H), 4.63 (d, J = 16.1 Hz, 1H), 4.14 (dt, J = 6.7, 0.9 Hz, 1H), 4.06 (q, J = 7.1 Hz, 2H), 3.71 (s, 3H), 3.23 (s, 2H), 2.76 (dd, J = 19.2, 6.7 Hz, 1H), 2.43 (dd, J = 19.2, 1.7 Hz, 1H), 1.18 (t, J = 7.1 Hz, 3H). **^{13}C NMR (75 MHz, $CDCl_3$)** δ 204.6, 171.9, 171.4, 160.6, 137.0, 135.1, 120.9, 114.8, 128.5, 61.7, 60.6, 55.4, 46.9, 42.0, 28.5, 14.2. **HRMS (ESI-MS)** *m/z* calcd for compound $C_{16}H_{19}O_4S$ [M + H]$^+$ 337.11042, found 337.11038.

Diallyamine 2-(2-(hydroxymethyl)-5-oxo-3-(propylthio)cyclopent-1-en-1-yl)aceticetate **3m**

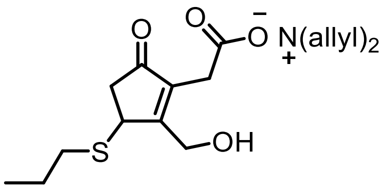

The titled compound was prepared according to general procedure using water as solvent. The crude mixture was purified by flash chromatography using hexane:ethyl acetate (9:1 to 2:8) affording 119 mg (87% yield) of pure product as a yellow oil.

^{1}H NMR (300 MHz, D_2O) δ 5.88 – 5.79 (m, 2H), 5.46 – 5.39 (m, 4H), 4.60 (d, J = 15.5 Hz, 1H), 4.49 (d, J = 15.6 Hz, 1H), 4.12 (m. 1H), 3.58 (d, J = 6.7 Hz, 4H), 3.23 – 3.09 (m, 2H), 3.00 (dd, J = 19.6, 6.5 Hz, 1H), 2.52 (dd, J = 19.7, 1.5 Hz, 1H), 2.43 – 2.36 (m, 2H), 1.55 – 1.43 (m, 2H), 0.86

(t, J = 7.3 Hz, 4H).[13]**C NMR (75 MHz, D_2O)** δ 210.6, 177.2, 173.2, 137.5, 127.4, 123.7, 58.1, 48.6, 43.2, 42.4, 31.0, 30.9, 22.4, 12.9. **HRMS (ESI-MS)** *m/z* calcd for compound $C_{11}H_{15}O_4S$ [M – $C_6H_{10}N$ + H]$^+$ 245.08421, found 245.08412.

Diallyamine 3-((3-(carboxymethyl)-2-(hydroxymethyl)-4-oxocyclopent-2-en-1-yl)thio)propanate **3n**

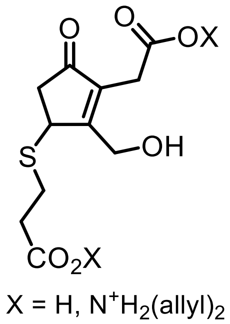

The titled compound was prepared according to general procedure using water as solvent. The crude mixture was purified by flash chromatography using hexane:ethyl acetate (9:1 to 2:8) affording 137 mg (92% yield) of pure product as a yellow oil.

[1]**H NMR (300 MHz, D_2O)** δ 5.96 – 5.87 (m, 2H), 5.54 – 5.48 (m, 4H), 4.73 – 4.56 (m, 2H), 4.22 (m, 1H), 3.67 (d, J = 6.7 Hz, 4H), 3.33 (d, J = 4.0 Hz, 1H), 2.83 – 2.51 (m, 7H).[13]**C NMR (75 MHz, D_2O)** δ 210.3, 177.9, 176.1, 174.0, 136.9, 127.7, 123.9, 58.5, 48.9, 43.5, 33.6, 24.9. **HRMS (ESI-MS)** *m/z* calcd For compound $C_{11}H_{13}O_6S$ [M – $C_6H_{10}N$]$^-$ 273.04383, found 273.04401.

General Procedure for the synthesis of LCP-thioadducts in nucleophilic solvents

To a solution of **LCP** (100 mg, 0.40 mmol) in acetonitrile (or iPrOH) (4 mL) was added the corresponding thiol (3 equiv, 1.2 mmol) and corresponding amine (5 equiv, 2.0 mmol) and the mixture was allowed to stir for 12 h. The solvent was evaporated under reduced pressure and the crude mixture was purified by column chromatography.

3-(hydroxymethyl)-4-((4-methoxyphenyl)thio)-2-(2-morpholino-2-oxoethyl)cyclopent-2-en-1-one **4a**

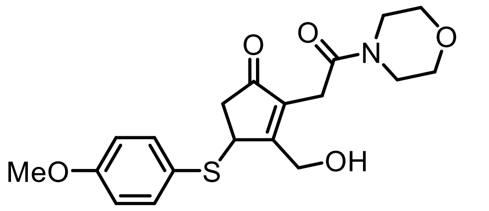

The titled compound was prepared according to general procedure. The crude mixture was purified by flash chromatography using hexane:ethyl acetate (7:3 to 1:9) affording 106 mg (70% yield) of pure product as a yellow oil in acetonitrile and 125 mg (82% yield) of pure product in iPrOH.

[1]**H NMR (300 MHz, $CDCl_3$)** δ 7.30 (d, J = 8.8 Hz, 2H), 6.80 (d, J = 8.8 Hz, 2H), 5.01 (d, J = 17.8 Hz, 1H), 4.62 (d, J = 17.8 Hz, 1H), 4.04 (d, J = 6.5 Hz, 1H), 3.78 (s, 3H), 3.69 – 3.52 (m, 8H), 3.49 –

3.24 (m, 2H), 2.82 (dd, J = 19.3, 6.7 Hz, 1H), 2.50 (dd, J = 19.3, 1.7 Hz, 1H). **^{13}C NMR (75 MHz, $CDCl_3$)** δ 205.0, 174.6, 169.8, 160.7, 137.4, 135.5, 120.8, 114.8, 66.9, 66.7, 62.1, 55.5, 47.3, 47.1, 42.9, 42.2, 25.9. **HRMS (ESI-MS)** *m/z* calcd for compound $C_{19}H_{24}NO_5S$ $[M + H]^+$ 378.13697, found 378.13687.

N,N-diallyl-2-(2-(hydroxymethyl)-3-((4-methoxyphenyl)thio)-5-oxocyclopent-1-en-1-yl)acetamide **4b**

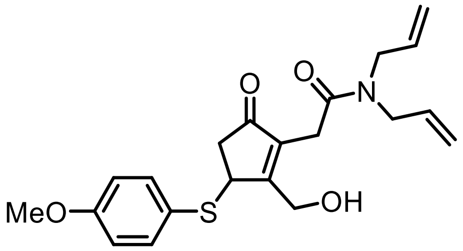

The titled compound was prepared according to general procedure. The crude mixture was purified by flash chromatography using hexane:ethyl acetate (7:3 to 1:9) affording 131 mg (84% yield) of pure product as a yellow oil in acetonitrile and 119 mg (76% yield) of pure product in iPrOH.

^{1}H NMR (300 MHz, $CDCl_3$) δ 7.28 (d, J = 8.8 Hz, 2H), 6.78 (d, J = 8.8 Hz, 2H), 5.80 – 5.62 (m, 2H), 5.42 – 5.03 (m, 4H), 4.96 (d, J = 17.4 Hz, 1H), 4.60 (d, J = 17.5 Hz, 1H), 4.33 – 3.82 (m, 5H), 3.76 (s, 3H), 3.60 – 3.23 (m, 2H), 2.79 (dd, J = 19.2, 6.7 Hz, 1H), 2.46 (dd, J = 19.2, 1.7 Hz, 1H). **^{13}C NMR (75 MHz, $CDCl_3$)** δ 205.2, 174.4, 171.4, 160.5, 137.2, 135.7, 132.7, 132.3, 120.8, 117.9, 117.1, 114.7, 61.8, 55.4, 50.2, 48.9, 47.3, 41.9, 26.5. **HRMS (ESI-MS)** *m/z* calcd for compound $C_{21}H_{26}NO_4S$ $[M + H]^+$ 388.15771, found 388.15762.

N,N-dibenzyl-2-(2-(hydroxymethyl)-3-((4-methoxyphenyl)thio)-5-oxocyclopent-1-en-1-yl)acetamide **4c**

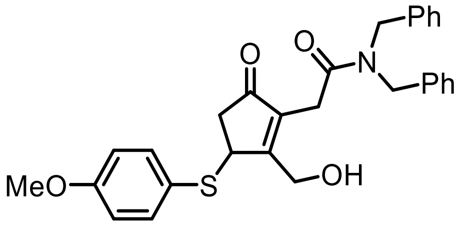

The titled compound was prepared according to general procedure. The crude mixture was purified by flash chromatography using hexane:ethyl acetate (7:3 to 1:9) affording 128 mg (65% yield) of pure product as a yellow oil in acetonitrile and 157 mg (80% yield) of pure product is iPrOH.

^{1}H NMR (300 MHz, $CDCl_3$) δ 7.47 – 7.08 (m, 7H), 6.79 (d, J = 8.8 Hz, 2H), 5.03 (d, J = 17.8 Hz, 1H), 4.64 (d, J = 17.8 Hz, 1H), 4.34 (m, 2H), 4.04 (m, 1H), 3.84 – 3.75 (m, 5H), 3.26 (m, 2H), 2.80 (dd, J = 19.2, 6.7 Hz, 1H), 2.48 (dd, J = 19.3, 1.7 Hz, 1H). **^{13}C NMR (75 MHz, $CDCl_3$)** δ 205.4, 174.2, 170.6, 160.6, 137.4, 137.3, 135.1, 128.9, 128.6, 128.6, 127.9, 127.8, 120.6, 114.8, 61.9, 55.4, 46.9, 44.2, 42.1, 30.8. **HRMS (ESI-MS)** *m/z* calcd for compound $C_{29}H_{30}NO_4S$ $[M + H]^+$ 488.18901, found 488.18906.

N,N-diallyl-2-(2-(hydroxymethyl)-5-oxo-3-(phenylthio)cyclopent-1-en-1-yl)acetamide **4d**

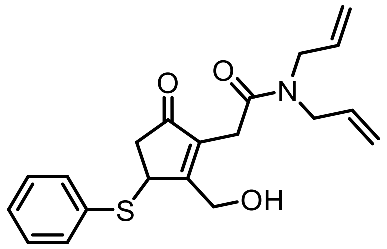

The titled compound was prepared according to general procedure. The crude mixture was purified by flash chromatography using hexane:ethyl acetate (7:3 to 1:9) affording 114 mg (79% yield) of pure product as a yellow oil in acetonitrile and 118 mg (82% yield) of pure product in iPrOH.

^{1}H NMR (300 MHz, $CDCl_3$) δ 7.37 - 7.35(m, 2H), 7.29 – 7.27 (m, 3H), 5.84 – 5.64 (m, 2H), 5.25 – 5.05 (m, 4H), 4.93 (d, J = 17.6 Hz, 1H), 4.60 (dd, J = 17.9, 5.5 Hz, 1H), 4.23 – 3.84 (m, 5H), 3.49 (d, J = 14.1 Hz, 1H), 3.39 (d, J = 14.1 Hz, 1H), 2.88 (dd, J = 19.2, 6.7 Hz, 1H), 2.52 (dd, J = 19.2, 1.8 Hz, 1H). **^{13}C NMR (75 MHz, $CDCl_3$)** δ 205.0, 174.2, 171.4, 136.1, 133.7, 132.7, 132.4, 131.9, 129.3, 128.6, 117.9, 117.2, 61.8, 50.3, 49.0, 46.9, 42.7, 26.6. **HRMS (ESI-MS)** *m/z* calcd for compound $C_{20}H_{24}NO_3S$ [M + H]$^+$ 358.14714, found 358.14719.

Methyl (2-(2-(hydroxymethyl)-5-oxo-3-(phenylthio)cyclopent-1-en-1-yl)acetyl)glycinate **4e**

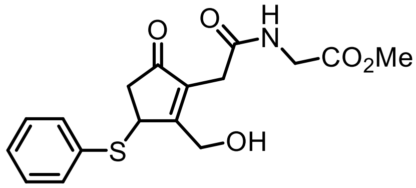

The titled compound was prepared according to general procedure in iPrOH. The crude mixture was purified by flash chromatography using hexane:ethyl acetate (7:3 to 1:9) affording 99 mg (70% yield) of pure product as a yellow oil.

^{1}H NMR (300 MHz, $CDCl_3$ δ 7.45 – 7.23 (m, 2H), 7.21 (dq, J = 3.5, 2.1, 1.7 Hz, 3H), 6.73 (s, 1H), 4.86 (d, J = 17.1 Hz, 1H), 4.56 (d, J = 17.2 Hz, 1H), 4.33 – 4.01 (m, 1H), 3.88 (dd, J = 5.5, 2.6 Hz, 2H), 3.66 (s, 3H), 3.26 (s, 2H), 2.82 (dd, J = 19.2, 6.7 Hz, 1H), 2.47 (dd, J = 19.3, 1.8 Hz, 1H).**^{13}C NMR (75 MHz, $CDCl_3$)** δ 205.3, 173.5, 170.9, 169.9, 135.5, 133.8, 131.8, 129.3, 128.6, 61.4, 52.6, 46.6, 42.8, 41.7, 30.5. **HRMS (ESI-MS)** *m/z* calcd for compound $C_{17}H_{19}NNaO_5S$ [M + Na]$^+$ 372.08761, found 372.08746.

Reduction of thio-adduct CP:

To a solution of a CP **3k** (100 mg, 0.32 mmol) in anhydrous MeOH (3.2 mL) was added $CeCl_3.7H_2O$ (121 mg, 0.32 mmol, 1 equiv) at -40 ºC. $LiBH_4$ (7 mg, 0.32 mmol, 1 equiv) was added and the reaction was stirred until total consumption of the starting material. The reaction was quenched by the addition of a sat. aq. sol. NH_4Cl (10 mL) and extracted with ethyl acetate (10 mL × 3). The combined organic phases were dried with anhydrous $MgSO_4$. The solvent was evaporated under reduced pressure. The crude mixture was purified by flash

chromatography using hexane:ethyl acetate (1:1) affording two fractions, one 47 mg (47% yield) of pure product as a yellow oil and the other diastereoisomer in 43 mg (43% yield) of pure product as a yellow oil.

Ethyl 2-((3,5)-5-hydroxy-2-(hydroxymethyl)-3-(phenylthio)cyclopent-1-en-1-yl)acetate **5a**

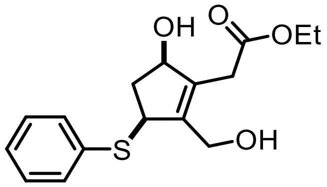

The titled compound was prepared according to reduction procedure. The crude mixture was purified by flash chromatography using hexane:ethyl acetate (1:1) affording 47 mg (47% yield) of pure product as a yellow oil.

^{1}H NMR (300 MHz, CDCl$_3$) δ 7.73 – 7.31 (m, 5H), 4.47 (d, J = 6.6 Hz, 1H), 4.41 (d, J = 4.5 Hz, 2H), 4.32 – 3.75 (m, 3H), 3.44 – 3.10 (m, 2H), 2.77 (dt, J = 15.3, 7.6 Hz, 1H), 1.90 (dt, J = 15.0, 2.9 Hz, 1H), 1.27 (t, J = 7.1 Hz, 3H). **^{13}C NMR (75 MHz, CDCl$_3$)** δ 171.9, 142.7, 137.1, 134.1, 133.2, 129.2, 128.0, 61.6, 58.2, 53.6, 52.7, 40.5, 32.3, 14.3. **HRMS (ESI-MS)** *m/z* calcd for compound $C_{16}H_{20}NaO_4S$ [M + Na]$^+$ 331.09745, found 331.09570.

Ethyl 2-((3,5)-5-hydroxy-2-(hydroxymethyl)-3-(phenylthio)cyclopent-1-en-1-yl)acetate **5b**

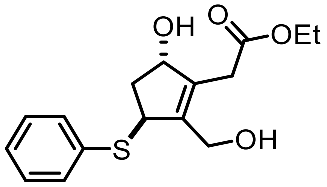

The titled compound was prepared according to reduction procedure. The crude mixture was purified by flash chromatography using hexane:ethyl acetate (1:1) affording 43 mg (43% yield) of pure product as a yellow oil.

^{1}H NMR (300 MHz, CDCl$_3$) δ 7.45 – 7.28 (m, 5H), 4.47 (s, 1H), 4.35 (s, 3H), 4.15 (q, J = 7.1 Hz, 2H), 3.38 (d, J = 15.7 Hz, 2H), 3.14 (d, J = 15.7 Hz, 1H), 2.50 (ddd, J = 14.1, 7.1, 2.3 Hz, 1H), 2.17 (ddd, J = 13.9, 8.0, 5.6 Hz, 1H), 1.26 (t, J = 7.1 Hz, 3H).**^{13}C NMR (75 MHz, CDCl$_3$)** δ 172.3, 141.8, 137.7, 134.4, 132.6, 129.1, 127.6, 61.6, 58.3, 52.7, 42.4, 32.2, 14.2. **HRMS (ESI-MS)** *m/z* calcd for compound $C_{16}H_{20}NaO_4S$ [M + Na]$^+$ 331.09745, found 331.09570.

Oxidation of thio-adduct CP to ethyl 2-(2-(hydroxymethyl)-5-oxo-3-(phenylsulfinyl)cyclopent-1-en-1-yl)acetate **6**

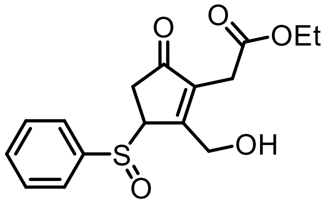

To a solution of CP **3k** (100 mg, 0.32 mmol) in DCM (3.2 mL) was added mCPBA (56 mg, 1 equiv, 0.32 mmol) at -40 ºC and the mixture was allowed to stir for 1 h. The reaction was

quenched with Na_2SO_3 (aq) and the mixture was extracted with DCM (2 x 5 mL). The solvent was evaporated under reduced pressure and the crude mixture was purified by column chromatography using hexane:ethyl acetate (7:3 to 1:9) affording 85 mg (81% yield) of a mixture of diastereoisomers as a brown oil.

^{1}H NMR (300 MHz, $CDCl_3$ δ 7.68 (dd, J = 7.7, 1.9 Hz, 2H), 7.62 – 7.38 (m, 8H), 4.91 – 4.49 (m, 4H), 4.54 – 4.35 (m, 1H), 4.07 (dq, J = 10.5, 7.1 Hz, 4H), 3.28 (dd, J = 16.7, 1.0 Hz, 1H), 3.24 – 3.08 (m, 2H), 2.57 (dd, J = 19.0, 1.9 Hz, 1H), 2.48 – 2.27 (m, 2H), 2.19 (dd, J = 19.0, 6.9 Hz, 1H), 1.54 – 1.02 (m, 6H). **^{13}C NMR (75 MHz, $CDCl_3$)** δ 202.7, 202.4, 169.8, 169.3, 167.1, 140.9, 137.8, 137.1, 132.7, 131.6, 129.6, 129.4, 125.4, 124.5, 65.3, 64.3, 61.4, 60.2, 59.6, 34.3, 31.1, 28.7, 28.7, 14.1, 14.1. **HRMS (ESI-MS)** *m/z* calcd for compound $C_{16}H_{19}O_5S$ $[M + H]^+$ 323.09477, found 323.09305.

Hydrazone formation of thio-adduct CP to tert-butyl (*E*)-2-(2-(2-ethoxy-2-oxoethyl)-3-(hydroxymethyl)-4-(phenylthio)cyclopent-2-en-1-ylidene)hydrazine-1-carboxylate **7**

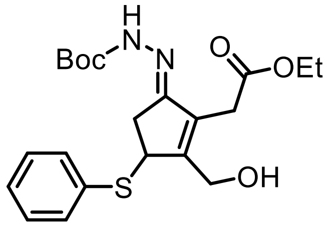

To a solution of CP **3k** (100 mg, 0.32 mmol) in anhydrous MeOH (3.2 mL) was added boc-hydrazine (2 equiv, 86 mg, 0.64 mmol). The solution was allowed to stir for 10 min. The solvent was evaporated under reduce pressure, the crude mixture was washed with cold diethyl ether to remove excess hydrazine. The product was further purified by flash chromatography using hexane:ethyl acetate (1:1) affording 111 mg (81% yield) of a pure product as a brown oil.

^{1}H NMR (300 MHz, $CDCl_3$) δ 7.33 – 7.28 (m, 2H), 7.22 (dd, J = 3.9, 2.5 Hz, 3H), 4.73 (d, J = 15.9 Hz, 1H), 4.61 (d, J = 16.0 Hz, 1H), 4.31 (dd, J = 6.7, 1.0 Hz, 1H), 4.06 (q, J = 7.2 Hz, 2H), 3.27 (s, 2H), 2.83 (dd, J = 19.2, 6.7 Hz, 1H), 2.47 (dd, J = 19.2, 1.8 Hz, 1H), 1.96 (s, 3H), 1.74 (s, 3H), 1.43 (s, 9H), 1.18 (t, J = 7.1 Hz, 3H). **^{13}C NMR (75 MHz, $CDCl_3$)** δ 204. 7, 171.9, 171.3, 135.3, 133.5, 131.8, 129.2, 128.5, 61.6, 60.3, 46.3, 42.5, 28.5, 28.4, 28.3, 25.4, 16.1, 14.1. **HRMS (ESI-MS)** *m/z* calcd For compound $C_{21}H_{29}N_2O_5S$ $[M + H]^+$ 421.17917, found 421.17700.

NMR studies

Monitorization of the reaction of 1 with thiols promoted in $MeOD_4$

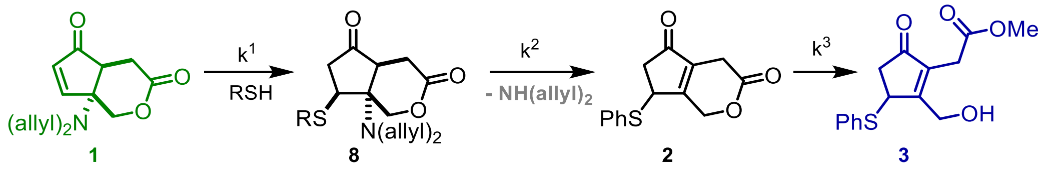

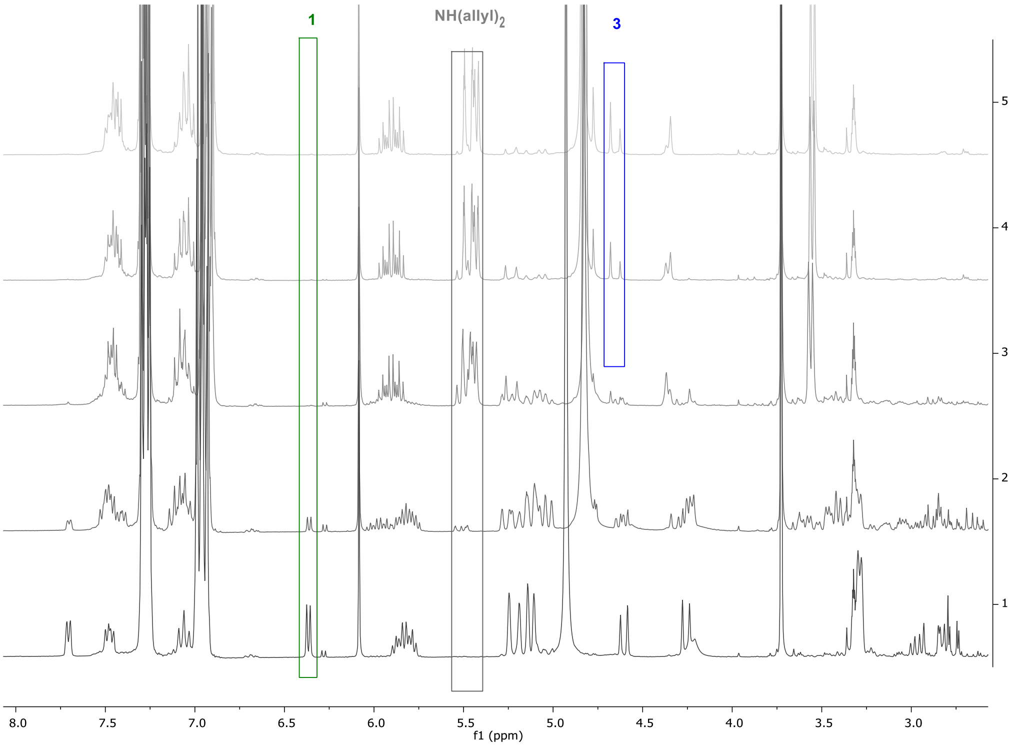

Figure S1. Selected ^{1}H NMR spectra of the reaction of **1** with F-PhSH in $MeOD_4$.

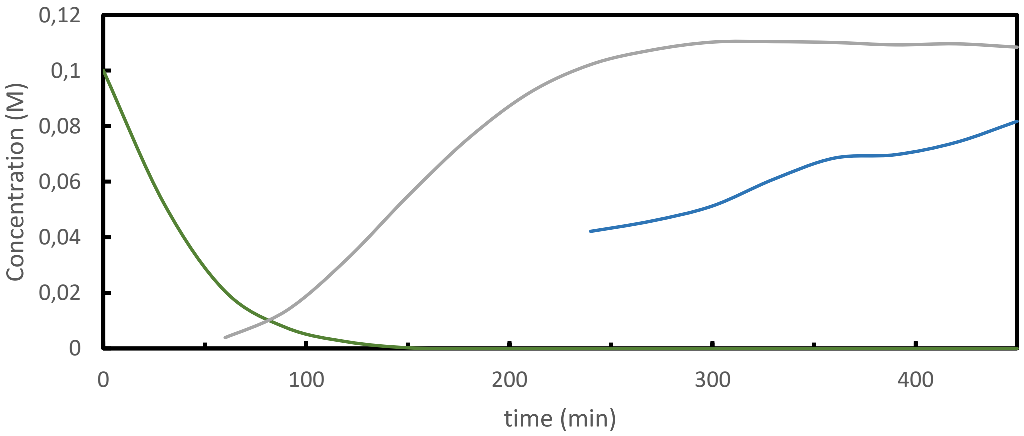

Figure S2. Reaction profile measured by ^{1}H-NMR spectroscopy. In green is the monoexponential decrease of **1**, in grey is the appearance of diallylamine, in blue is the formation of product **3** (due to overlapping signals, product **3** could not be measured prior to 240 minutes).

Kinetic study of the arylthiol substituent on the reaction

To a solution of **1** (10 mg, 0.04 mmol, 0.1 M) in $MeOD_4$ (0.4 mL) was added trimethoxybenzene (2 mg, 0.012 mmol) as the internal standard and ^{1}H NMR analysis was performed at room temperature (t = 0). Then, arylthiol (10 equiv, 0.4 mmol) was added and the reaction monitored by ^{1}H NMR every 3 min to trace the reaction profile.

Least-squares fitting of the monoexponential consumption of starting material **1** using the plot ln ([**1**]) vs time was performed to determine k^1**obs** (s^{-1}). Ln(k^1**obsR**/k^1**obsH**) was plotted against σ_{para} of the different aryl thiols to obtain an Hammett plot.

Table S1. Kinetics of the reaction of **1** with arylthiols in $MeOD_4$ (k^1_{obs}).

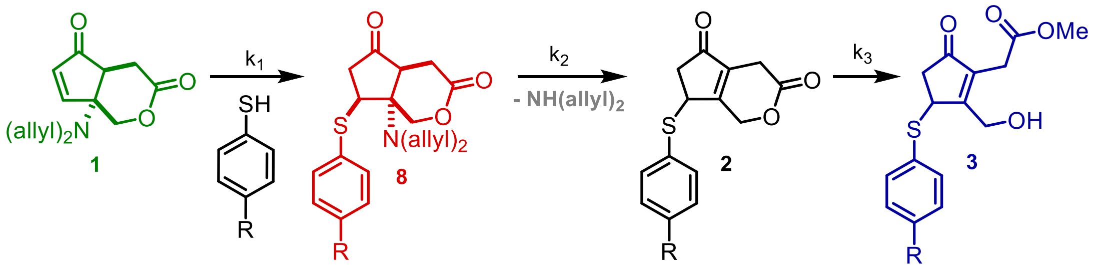

R	σ_{para}	k^1_{obs} (s^{-1})	Log(k^1_{obsR}/k^1_{obsH})
OMe	-0.268	4.87×10^{-2}	5.7×10^{-1}
Me	-0.17	3.39×10^{-2}	4.1×10^{-1}
H	0	1.30×10^{-2}	0
F	0.062	1.82×10^{-2}	1.4×10^{-1}
Cl	0.227	7.99×10^{-3}	-2.1×10^{-1}
CF_3	0.54	4.00×10^{-3}	-5.1×10^{-1}

Figure S3. Hammett plot values for the thioaryl substituents and k^1_{obs}.

Peptide Assays

General Procedure for the reaction optimization with Laminin Fragment

A solution of Laminin Fragment (1 mg/mL in H_2O, 0.925 mM, 5.4 µL, 0.005 µmol) was added to Acetate buffer 20 mM, (0.5 ml) followed by Tris(2-carboxyethyl)phosphine hydrochloride (TCEP) (3.5 mM in water, 1 mg/mL) and the solution was mixed for 2 hours at 25 ºC. Then cyclopentenone **1** (25 mM in DMF) was added and the mass checked in Positive Mode of ESI-MS.

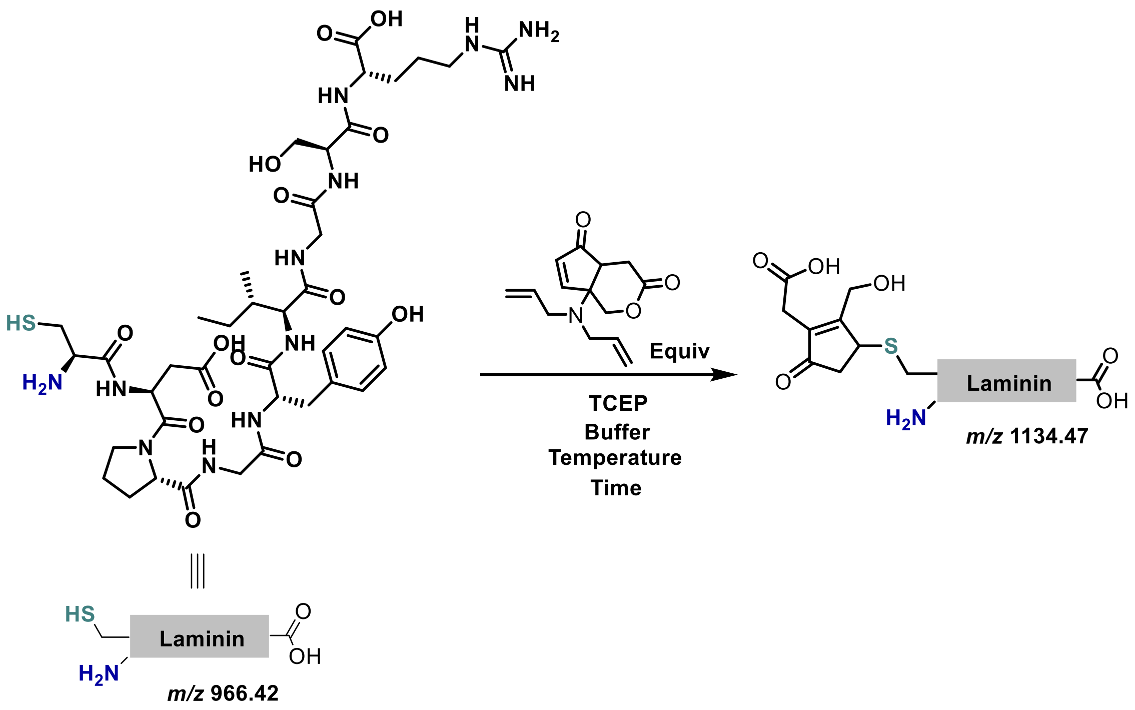

Scheme S1. Modification of laminin fragment with LCP **1**.

Table S3- Optimization for the modification of laminin fragment with LCP **1.** Best conversion in green, with corresponding ESI shown in Figure S5 left.

Entry	T (ºC)	pH	TCEP (equiv.)	1 (equiv.)
**1**	37	8	3	10
**2**	37	7.4	3	10
**3**	37	6	3	10
**4**	37	4.5	3	10
**5**	25	8	3	10
**6**	37	8	5	10
**7**	37	8	10	10
**8**	37	8	5	25
**9**	37	8	5	50
**10**	37	8	5	100

Laminin Fragment + reversibility

A solution of Laminin Fragment (1 mg/mL in H_2O, 0.925 mM, 5.4 µL, 0.005 µmol) was added to Acetate buffer 20 mM, pH8.0 (0.5 ml) followed by Tris(2-carboxyethyl)phosphine hydrochloride (TCEP) (3.5 mM in water, 1 mg/mL, 7.2 µL, 5 equiv) and the solution was mixed for 2 hours at 25 ºC. Then cyclopentenone **1** (25 mM in DMF, 6.5 µL, 25 equiv.) was added and the mass checked in Positive Mode of ESI-MS. Past 1h, 100 equiv of mercaptoethanol was

added . The solution was mixed for 12h, dialyzed, and the mass checked in Positive Mode of ESI-MS.

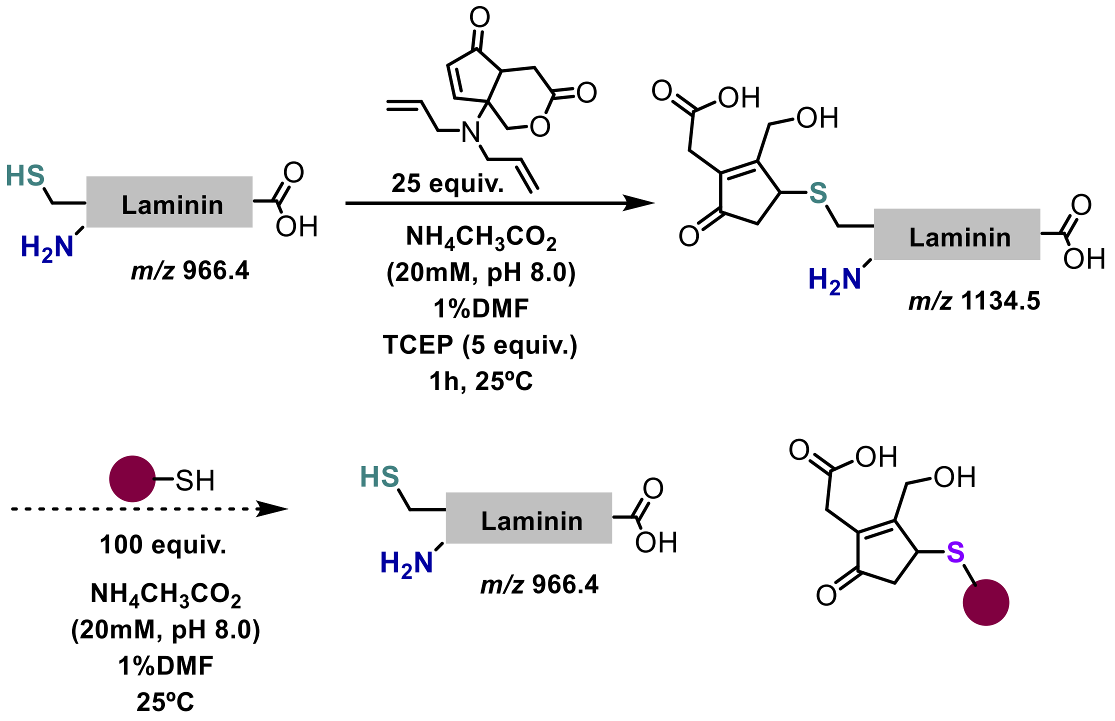

Scheme S2. Modification of laminin fragment with LCP **1**, followed by thiol addition to the modified peptide

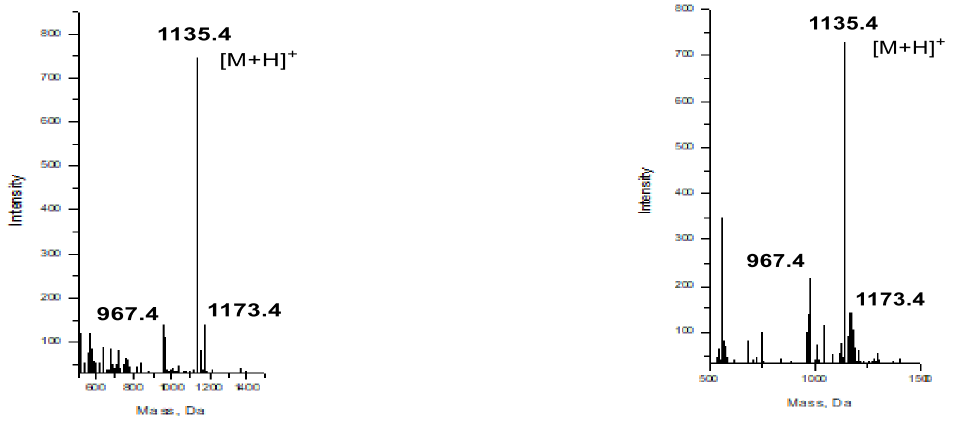

Figure S4. Raw spectra of the reaction of laminin fragment with LCP **1** after 2h in positive mode of ESI-MS (Left); Raw spectra of the reaction of the modified laminin fragment after with 100 equiv of mercaptoethanol, after dialysis, in positive mode of ESI-MS (right).

Ovalbumin modification

A solution of C-Ovalbumin (1mg/mL, 5.9 µL, 0.005 µmol) was added to Acetate buffer 20 mM pH = 8.0 + 1% DMF (0.5 ml) followed by Tris(2-carboxyethyl)phosphine hydrochloride (TCEP) (3.5 mM in water, 1 mg/mL, 4.3 µL, 0.015 µmol) and the solution mixed for 2 hour at 25 ºC. by Tris(2-carboxyethyl)phosphine hydrochloride (TCEP) (3.5 mM in water, 1 mg/mL, 7.2 µL, 5 equiv) and the solution was mixed for 2 hours at 25 ºC. Then cyclopentenone **1** (25 mM in DMF, 6.5 µL, 25 equiv.) was added and the mass checked in Positive Mode of ESI-MS.

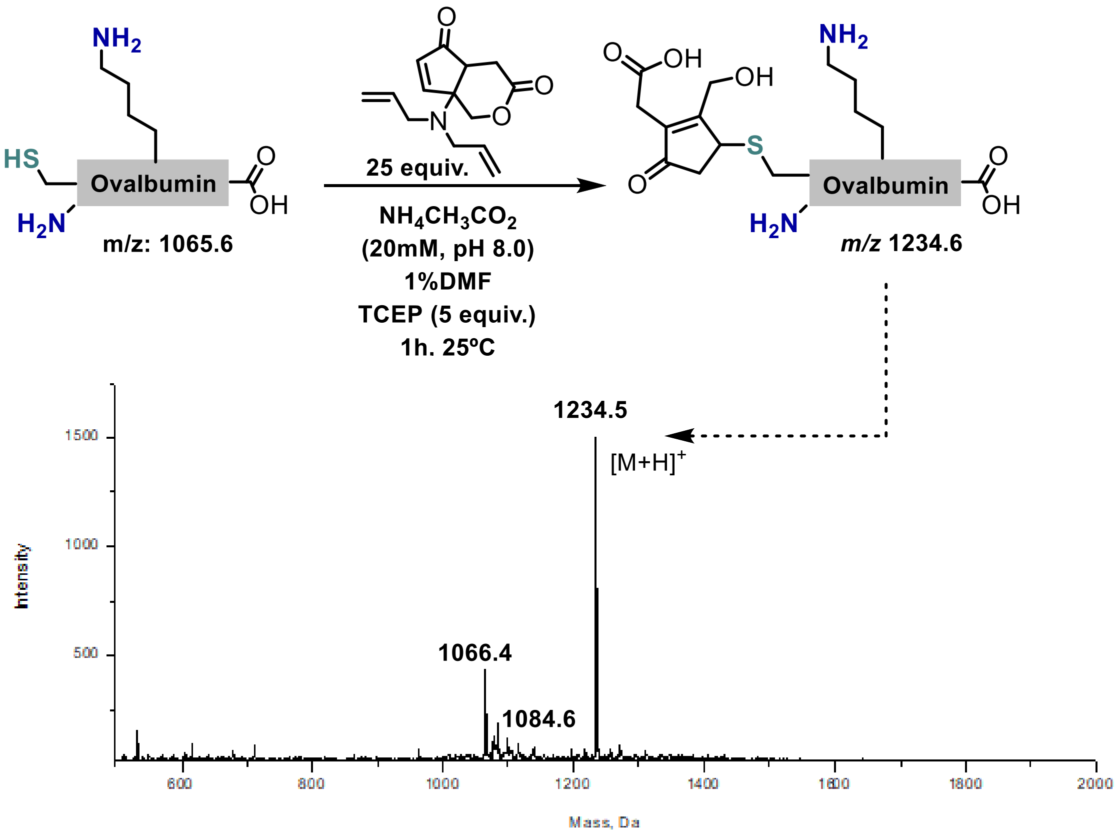

Scheme S3. Modification of ovalbumin fragment with LCP **1**. Raw spectra of the reaction in positive mode of ESI-MS

Computational studies

Geometry optimizations and frequency calculations were carried out using Gaussian 09 software, Revision D.01. Vibrational frequencies and intensities were calculated at the B3LYP/6-31G(d) level of theory, since such functional/basis-set combination was reported to provide a good energetics accuracy cycloadditions.[3-5] All the geometry optimizations were carried out at the B3LYP level of theory with the 6-31G(d) basis set. All of the optimized geometries were verified by frequency computations as minima (zero imaginary frequencies) or transition states (a single imaginary frequency corresponding to the desired reaction coordinate). Single-point energy calculations on the optimized geometries were then evaluated using the B3LYP-D3 functional and the 6-311+G(d,p) basis set. Solvent effects in methanol were evaluated at the B3LYP-D3/6-311+G(d,p) level with a self-consistent reaction field (SCRF) using the CPCM model (SMD) on the gas-phase-optimized structures. Graphical representation of the optimized geometries was generated by using CYLView.[6]

Table S3 - Summary of the calculations performed at B3LYP-D3/6-311+G(d,p) /SMD(MeOH) level of theory for pre-complexes, TS and products **Ia** and **IIa** to **Ic** and **IIc**

	B3LYP/6-31G(d)	B3LYP-D3/6-311+G(d,p) /SMD(MeOH)	
	ΔG (Hartree)	ΔG_{MeOH} (Hartree)	ΔG_{MeOH} (kcal/mol)
Ia	-901.095160	-900.816730	0.0
TSIa-Ib	-901.044646	-900.765257	32.3
Ib	-901.080498	-900.795061	13.6
Ib’	-901.082069	-900.797286	12.2
TSIb-Ic	-901.047531	-900.768157	30.5
Ic	-901.091990	-900.808699	5.0
IIa	-1204.637747	-1204.406210	0.0
TSIIa-IIb	-1204.593736	-1204.360415	28.7
IIb	-1204.628915	-1204.389301	10.6
IIb’	-1204.595908	-1204.390476	9.9
TSIIb-IIc	-1204.628594	-1204.363146	27.0
IIc	-1204.644971	-1204.410505	-2.7

Ia

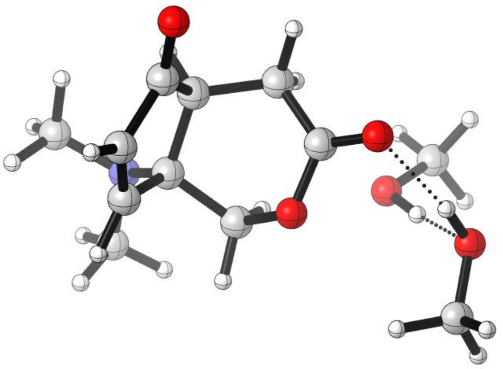

SCF energy = -901.095160325 Hartree
Thermal correction to Gibbs Free Energy = 0.278430238

6	1.057331000	-1.361499000	1.499385000
7	-0.364270000	-1.100385000	1.266221000
6	-0.634754000	-0.130892000	0.175404000
6	-0.096892000	1.291290000	0.522058000
6	-1.196540000	2.355379000	0.684040000
6	-2.211553000	2.306200000	-0.430570000
6	-2.163444000	-0.052025000	-0.048082000
8	-2.533733000	1.069498000	-0.874543000

6	0.080561000	-0.458608000	-1.133798000
6	0.907896000	0.506473000	-1.558291000
6	0.866741000	1.655058000	-0.626497000
8	1.456880000	2.712881000	-0.731391000
6	-1.086452000	-2.369583000	1.170171000
1	0.476316000	1.266578000	1.452400000
1	1.619428000	-0.429374000	1.592694000
1	1.535056000	-1.965156000	0.707766000
1	1.159461000	-1.903800000	2.445243000
1	-0.760158000	3.356048000	0.712405000
1	-1.749999000	2.191868000	1.616942000
1	-2.693924000	0.030187000	0.904746000
1	-2.538154000	-0.917343000	-0.596751000
1	-0.084411000	-1.404823000	-1.643458000
1	1.523826000	0.500544000	-2.451157000
1	-2.167192000	-2.212635000	1.186242000
1	-0.830962000	-2.970251000	0.276854000
1	-0.836540000	-2.971752000	2.049445000
8	-4.436678000	1.514036000	1.482826000
6	-5.100605000	2.098849000	2.588748000
1	-6.150318000	1.775264000	2.664768000
1	-4.581789000	1.776708000	3.497614000
1	-5.084339000	3.198989000	2.556005000
8	-2.726343000	3.276959000	-0.948663000
1	-4.566054000	2.967577000	-1.113175000
1	-4.886939000	1.825390000	0.663307000
6	-5.822924000	1.701845000	-1.926052000
8	-5.439773000	2.587892000	-0.880251000
1	-6.025057000	2.246115000	-2.859194000
1	-5.056772000	0.939719000	-2.120415000
1	-6.745258000	1.205793000	-1.610983000

TS $_{Ia\text{-}Ib}$

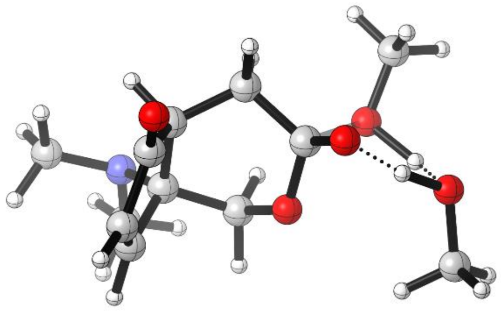

SCF energy = -901.044645765 Hartree

Thermal correction to Gibbs Free Energy = 0.279389168

Im.Freq= 1399.3556 icm^{-1}

6	1.104810000	-1.331973000	1.468066000
7	-0.341171000	-1.224562000	1.265357000
6	-0.742289000	-0.224034000	0.246772000
6	-0.391112000	1.234407000	0.677863000
6	-1.594404000	2.076392000	1.134649000
6	-2.727628000	2.025601000	0.113651000
6	-2.277564000	-0.299983000	0.032675000
8	-2.742755000	0.867260000	-0.643273000
6	-0.023359000	-0.364309000	-1.092275000
6	0.566581000	0.762925000	-1.512212000

6	0.354711000	1.845297000	-0.525040000
8	0.709066000	3.004619000	-0.612631000
6	-0.901621000	-2.562144000	1.071518000
1	0.318050000	1.214374000	1.510627000
1	1.554283000	-0.350650000	1.635921000
1	1.637673000	-1.806642000	0.624957000
1	1.287354000	-1.935359000	2.363783000
1	-1.288070000	3.118987000	1.247877000
1	-1.935355000	1.703879000	2.103428000
1	-2.787034000	-0.400159000	0.996671000
1	-2.559507000	-1.136007000	-0.610876000
1	-0.023606000	-1.305769000	-1.636527000
1	1.116950000	0.913579000	-2.434798000
1	-1.993599000	-2.539331000	1.078134000
1	-0.567492000	-3.061268000	0.142082000
1	-0.586936000	-3.192046000	1.909970000
8	-4.214784000	1.646667000	1.090716000
6	-4.486729000	2.601451000	2.108842000
1	-5.541584000	2.525530000	2.399196000
1	-3.873520000	2.402534000	2.996623000
1	-4.290343000	3.625592000	1.761861000
8	-3.025649000	3.084652000	-0.540665000
1	-4.205757000	2.899382000	-0.949090000
1	-4.981828000	1.952644000	0.102850000
6	-5.690154000	1.632200000	-1.930027000
8	-5.317529000	2.507489000	-0.861920000
1	-5.872590000	2.231213000	-2.827459000
1	-4.904400000	0.896786000	-2.135806000
1	-6.615787000	1.114210000	-1.659279000

Ib

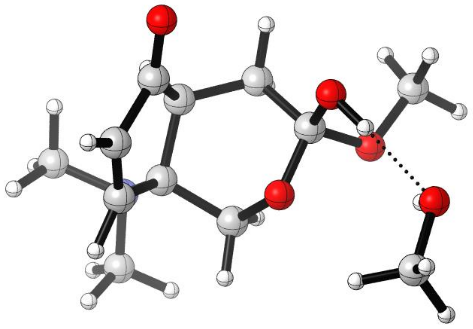

SCF energy = -900.795061 Hartree

Thermal correction to Gibbs Free Energy = 0.285436358

6	1.174390000	-1.358281000	1.311467000
7	-0.285694000	-1.283402000	1.237315000
6	-0.798305000	-0.246720000	0.308778000
6	-0.448191000	1.199592000	0.782483000
6	-1.623711000	1.978170000	1.389116000
6	-2.869674000	1.958945000	0.486748000
6	-2.344650000	-0.351365000	0.218239000
8	-2.879840000	0.828844000	-0.362391000
6	-0.195157000	-0.299684000	-1.091582000
6	0.323525000	0.864852000	-1.503195000
6	0.179795000	1.889781000	-0.444362000
8	0.521785000	3.054776000	-0.494060000
6	-0.826089000	-2.625637000	1.021977000

1	0.332809000	1.156865000	1.548195000
1	1.611884000	-0.371575000	1.478673000
1	1.643778000	-1.785067000	0.407152000
1	1.449290000	-1.990184000	2.163066000
1	-1.320821000	3.015910000	1.550950000
1	-1.877269000	1.536271000	2.358016000
1	-2.763891000	-0.522142000	1.217929000
1	-2.659250000	-1.167766000	-0.436367000
1	-0.215811000	-1.214152000	-1.679854000
1	0.788096000	1.076615000	-2.460386000
1	-1.913651000	-2.636926000	1.124012000
1	-0.562533000	-3.066012000	0.041520000
1	-0.422268000	-3.287895000	1.794871000
8	-4.088122000	1.814971000	1.246934000
6	-4.374508000	2.887827000	2.145077000
1	-5.378591000	2.702368000	2.533345000
1	-3.668928000	2.909175000	2.985878000
1	-4.349583000	3.853037000	1.629004000
8	-2.901623000	3.088696000	-0.292606000
1	-3.713075000	3.021152000	-0.853180000
1	-5.306862000	1.930135000	-0.340958000
6	-5.612119000	1.482924000	-2.232725000
8	-5.449904000	2.422893000	-1.173615000
1	-5.702787000	2.056984000	-3.158616000
1	-4.750686000	0.808445000	-2.312946000
1	-6.526689000	0.886685000	-2.105314000

Ib’

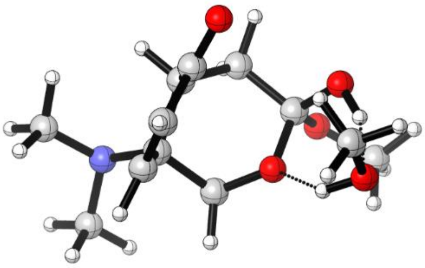

SCF energy = -900.797286 Hartree

Thermal correction to Gibbs Free Energy = 0.284782229

6	1.136525000	-1.370859000	0.815468000
7	-0.242464000	-1.185802000	1.269051000
6	-1.053032000	-0.288895000	0.415017000
6	-0.551314000	1.191630000	0.437853000
6	-1.315482000	2.117248000	1.391618000
6	-2.834118000	2.079528000	1.156195000
6	-2.526575000	-0.279011000	0.917402000
8	-3.197771000	0.875015000	0.430674000
6	-1.029960000	-0.632453000	-1.070139000
6	-0.789742000	0.422577000	-1.860581000
6	-0.575271000	1.635433000	-1.036581000
8	-0.422372000	2.772997000	-1.440240000
6	-0.842125000	-2.493927000	1.532483000
1	0.500781000	1.210999000	0.741518000
1	1.621888000	-0.410977000	0.625931000
1	1.224132000	-1.980529000	-0.101875000
1	1.700784000	-1.874396000	1.607778000
1	-0.979636000	3.147930000	1.252752000

1	-1.110128000	1.823949000	2.424595000
1	-2.544638000	-0.284316000	2.013365000
1	-3.091698000	-1.139896000	0.550837000
1	-1.221798000	-1.643889000	-1.421038000
1	-0.752867000	0.439002000	-2.945042000
1	-1.817656000	-2.393921000	2.013190000
1	-0.960258000	-3.125639000	0.631171000
1	-0.193708000	-3.037696000	2.227339000
8	-3.435942000	2.051978000	2.425130000
6	-4.847219000	2.253481000	2.436688000
1	-5.159402000	2.134141000	3.476390000
1	-5.103679000	3.259814000	2.089528000
1	-5.365390000	1.511897000	1.815815000
8	-3.241896000	3.142852000	0.382943000
1	-3.966898000	2.863792000	-0.226514000
1	-4.247679000	1.246570000	-1.245969000
6	-4.073292000	2.458812000	-2.785543000
8	-4.747785000	2.000567000	-1.610282000
1	-4.070864000	1.689949000	-3.570158000
1	-4.631586000	3.323553000	-3.152101000
1	-3.041885000	2.767254000	-2.573165000

TS $_{Ib-Ic}$

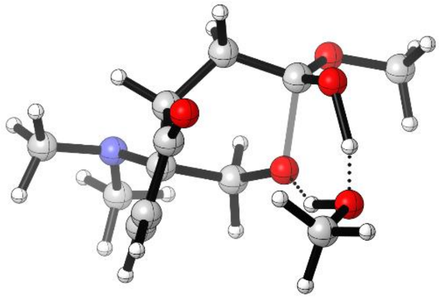

SCF energy = -900.768157 Hartree

Thermal correction to Gibbs Free Energy = 0.279374369

Im.Freq= 1008.097 icm^{-1}

6	1.101833000	-1.316507000	0.792551000
7	-0.290715000	-1.169447000	1.216690000
6	-1.114894000	-0.299236000	0.345040000
6	-0.666434000	1.197761000	0.393327000
6	-1.359831000	2.122424000	1.403377000
6	-2.868341000	2.325793000	1.242123000
6	-2.614951000	-0.365205000	0.785693000
8	-3.327450000	0.742043000	0.342730000
6	-1.021215000	-0.623535000	-1.137337000
6	-0.839424000	0.456308000	-1.911942000
6	-0.722564000	1.666472000	-1.067834000
8	-0.652978000	2.821011000	-1.453653000
6	-0.851202000	-2.494113000	1.482551000
1	0.398567000	1.220729000	0.654048000
1	1.563704000	-0.344522000	0.604777000
1	1.227476000	-1.931065000	-0.117566000
1	1.665774000	-1.797107000	1.599579000
1	-0.910581000	3.117720000	1.317147000
1	-1.163950000	1.748037000	2.412044000
1	-2.639664000	-0.389142000	1.885604000

1	-3.086104000	-1.288313000	0.419421000
1	-1.132588000	-1.641776000	-1.503243000
1	-0.780078000	0.489209000	-2.995243000
1	-1.834377000	-2.422962000	1.950810000
1	-0.938638000	-3.134981000	0.583726000
1	-0.192088000	-3.012369000	2.187169000
8	-3.481137000	2.103062000	2.438783000
6	-4.877684000	2.410439000	2.482106000
1	-5.195681000	2.195378000	3.503724000
1	-5.051649000	3.462018000	2.240348000
1	-5.425388000	1.776082000	1.777849000
8	-3.333372000	3.217627000	0.457537000
1	-4.003962000	2.735621000	-0.575613000
1	-3.870091000	1.174236000	-0.791762000
6	-3.866983000	2.309288000	-2.648635000
8	-4.379669000	2.006584000	-1.336152000
1	-3.936701000	1.407440000	-3.261649000
1	-4.500282000	3.086915000	-3.082330000
1	-2.831618000	2.657183000	-2.597204000

Ic

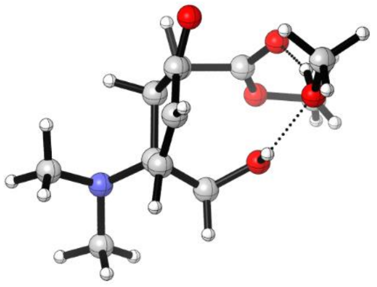

SCF energy = -900.808699 Hartree

Thermal correction to Gibbs Free Energy = 0.283291792

6	1.071930000	-1.282723000	0.657635000
7	-0.274870000	-1.161702000	1.219583000
6	-1.210507000	-0.337205000	0.407254000
6	-0.803336000	1.177514000	0.461533000
6	-1.347721000	2.119494000	1.553645000
6	-2.799225000	2.562980000	1.476543000
6	-2.670722000	-0.583107000	0.889713000
8	-3.604684000	0.343787000	0.398195000
6	-1.158343000	-0.622686000	-1.090161000
6	-1.041430000	0.476874000	-1.847573000
6	-0.912851000	1.677887000	-0.988616000
8	-0.820944000	2.834372000	-1.355186000
6	-0.742028000	-2.490744000	1.614271000
1	0.278036000	1.176217000	0.644988000
1	1.464341000	-0.312062000	0.345685000
1	1.130665000	-1.961272000	-0.212443000
1	1.741529000	-1.673955000	1.431483000
1	-0.758917000	3.041877000	1.484368000
1	-1.157610000	1.672871000	2.533097000
1	-2.702357000	-0.486741000	1.980005000
1	-2.966236000	-1.610588000	0.631413000
1	-1.248755000	-1.637136000	-1.471906000
1	-1.025838000	0.531395000	-2.930817000
1	-1.650169000	-2.434899000	2.216584000
1	-0.925673000	-3.176753000	0.765194000
1	0.031442000	-2.949062000	2.239445000
8	-3.474034000	2.206048000	2.583874000
6	-4.873386000	2.537259000	2.582916000
1	-5.251144000	2.209587000	3.552062000
1	-5.014480000	3.613596000	2.457490000
1	-5.373087000	2.003295000	1.771470000
8	-3.281781000	3.249415000	0.593951000
1	-3.843356000	2.533103000	-1.064861000
1	-3.587262000	0.437713000	-0.576555000
6	-3.785749000	2.516281000	-3.028051000
8	-4.045124000	1.894209000	-1.776802000
1	-3.935770000	1.759846000	-3.804807000
1	-4.483277000	3.344741000	-3.220141000
1	-2.758185000	2.896802000	-3.091866000

IIa

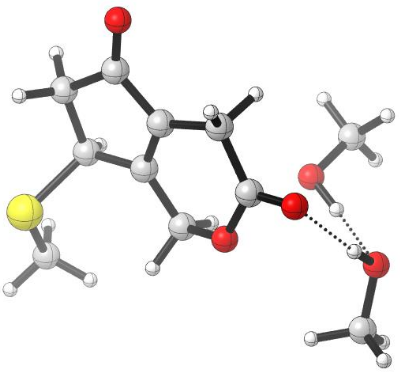

SCF energy = -1204.406210 Hartree

Thermal correction to Gibbs Free Energy = 0.231536530

8	2.843356000	2.848792000	0.445427000
6	1.732640000	2.354629000	0.483683000
6	1.404224000	0.933790000	0.715271000
6	0.080529000	0.736626000	0.625503000
6	-0.511790000	-0.616298000	0.806910000
8	0.373167000	-1.542047000	1.478750000
6	1.717845000	-1.456036000	1.426012000
8	2.373280000	-2.411377000	1.796827000
1	1.668309000	-3.967816000	1.038022000
8	0.913974000	-2.313041000	-1.293588000
6	1.462250000	-2.555273000	-2.577160000
6	2.376021000	-0.168767000	0.964783000
6	-0.687212000	1.994697000	0.274655000
16	-2.099659000	2.387488000	1.408902000
6	-3.488239000	1.568020000	0.545200000
6	0.399175000	3.096915000	0.309321000
1	-1.407479000	-0.577855000	1.433594000
1	-0.758903000	-1.065976000	-0.162617000
1	2.487228000	-2.952588000	-2.527221000
1	0.852736000	-3.256732000	-3.167952000
1	1.492647000	-1.600427000	-3.112309000
1	3.132854000	0.108253000	1.707477000
1	2.930962000	-0.413465000	0.049675000
1	-1.122057000	1.905226000	-0.728647000
1	-3.388266000	0.478915000	0.529195000
1	-4.392428000	1.824569000	1.103607000
1	-3.590073000	1.944565000	-0.477144000

1	0.262416000	3.775059000	1.159517000
1	0.411263000	3.716317000	-0.592136000
1	0.938907000	-3.158561000	-0.786832000
8	1.187033000	-4.511810000	0.378678000
6	0.020842000	-5.029118000	1.009818000
1	-0.595903000	-4.234063000	1.449595000
1	-0.561899000	-5.547627000	0.243301000
1	0.278005000	-5.752334000	1.796117000

TS$_{IIa-IIb}$

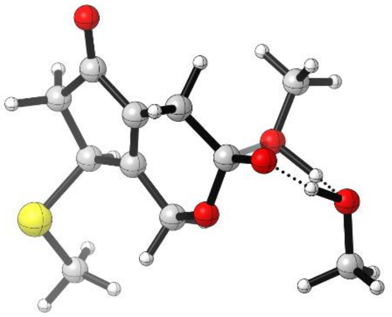

SCF energy = -1204.360415 Hartree

Thermal correction to Gibbs Free Energy = 0.233320980

Im.Freq= 1364.4940icm^{-1}

8	2.782281000	2.501278000	-0.365074000
6	1.676566000	2.088008000	-0.068389000
6	1.339205000	0.739655000	0.427649000
6	0.008340000	0.605734000	0.550756000
6	-0.612067000	-0.676718000	0.994144000
8	0.344201000	-1.632928000	1.471160000
6	1.592288000	-1.701228000	0.863286000
8	2.298858000	-2.697568000	1.242579000
1	1.665573000	-3.738067000	0.839659000
8	0.998828000	-2.109534000	-0.786247000
6	1.993527000	-2.081310000	-1.803478000
6	2.302942000	-0.364717000	0.709332000
6	-0.754726000	1.844249000	0.130126000
16	-1.970353000	2.478181000	1.378765000
6	-3.479899000	1.560269000	0.907079000
6	0.361934000	2.878863000	-0.146156000
1	-1.295433000	-0.506987000	1.833816000
1	-1.184901000	-1.119567000	0.167243000
1	2.945147000	-2.498870000	-1.446259000
1	1.647365000	-2.680237000	-2.654173000
1	2.155456000	-1.053224000	-2.147704000
1	2.836754000	-0.192376000	1.653500000
1	3.076027000	-0.427952000	-0.061144000
1	-1.334692000	1.642757000	-0.779488000
1	-3.386772000	0.484331000	1.079575000
1	-4.284666000	1.944143000	1.539753000
1	-3.740215000	1.745254000	-0.139702000
1	0.383385000	3.667161000	0.615500000
1	0.258122000	3.375526000	-1.115073000
1	0.825800000	-3.339583000	-0.467675000
8	0.999568000	-4.341490000	0.101735000
6	-0.177424000	-4.863578000	0.728579000
1	-0.638674000	-4.120405000	1.388424000
1	-0.889268000	-5.162087000	-0.047043000
1	0.099075000	-5.746890000	1.311682000

IIb

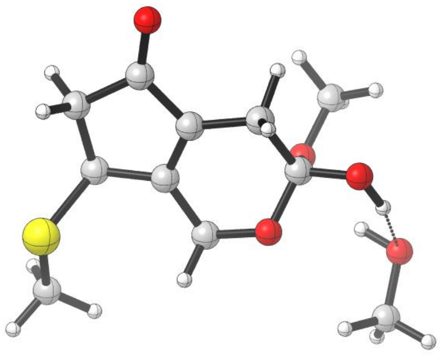

SCF energy = -1204.389301 Hartree

Thermal correction to Gibbs Free Energy = 0.239613730

8	2.753091000	2.648108000	-0.268845000
6	1.655701000	2.193403000	-0.002722000
6	1.344364000	0.806266000	0.393202000
6	0.014315000	0.637606000	0.496964000
6	-0.575413000	-0.692776000	0.839556000
8	0.415176000	-1.637116000	1.250872000
6	1.617299000	-1.651580000	0.497499000
8	2.435390000	-2.625139000	1.004708000
1	1.959452000	-3.485665000	0.896181000
8	1.177940000	-1.967049000	-0.837774000
6	2.203446000	-2.062111000	-1.828163000
6	2.332160000	-0.297933000	0.594093000
6	-0.770472000	1.888656000	0.163370000
16	-2.007785000	2.402987000	1.445226000
6	-3.491157000	1.483439000	0.899776000
6	0.326562000	2.963319000	-0.024152000
1	-1.263284000	-0.604428000	1.688010000
1	-1.145576000	-1.080624000	-0.019408000
1	3.055434000	-2.641001000	-1.456904000
1	1.758691000	-2.575853000	-2.683689000
1	2.543101000	-1.069697000	-2.149210000
1	2.788679000	-0.244784000	1.588942000
1	3.153464000	-0.222506000	-0.125036000
1	-1.339803000	1.745996000	-0.764003000
1	-3.371937000	0.400782000	0.998238000
1	-4.309269000	1.802747000	1.550929000
1	-3.749454000	1.733982000	-0.133835000
1	0.329811000	3.687846000	0.798772000
1	0.216829000	3.534375000	-0.950451000
1	0.504174000	-3.833904000	-0.396893000
8	0.739224000	-4.625113000	0.126350000
6	-0.368616000	-4.961962000	0.960411000
1	-0.648452000	-4.129777000	1.617929000
1	-1.241390000	-5.265060000	0.365585000
1	-0.058409000	-5.811791000	1.573499000

IIb’

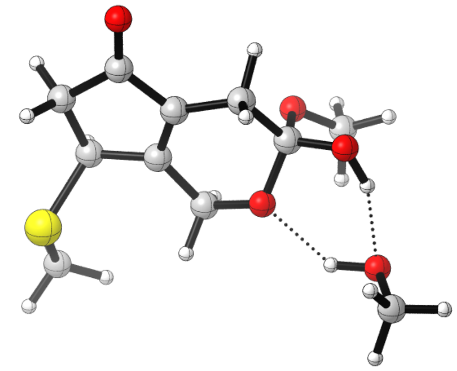

SCF energy = -1204.390476 Hartree

Thermal correction to Gibbs Free Energy = 0.238118570

8	2.746558000	2.823757000	0.930275000
6	1.650331000	2.324580000	0.758257000
6	1.345779000	0.888565000	0.596013000
6	0.036452000	0.717157000	0.343729000
6	-0.541634000	-0.642618000	0.113007000
8	0.372687000	-1.677538000	0.485083000
6	1.740086000	-1.489564000	0.016153000
8	2.446603000	-2.595600000	0.419448000
1	1.878895000	-3.398510000	0.328384000
8	1.723402000	-1.283395000	-1.375084000
6	1.484377000	-2.440027000	-2.172580000
6	2.336981000	-0.230436000	0.650057000
6	-0.729790000	2.019848000	0.257085000
16	-2.247606000	2.106045000	1.321067000
6	-3.525435000	1.486784000	0.168677000
6	0.315944000	3.081087000	0.672529000
1	-1.430439000	-0.796659000	0.734843000
1	-0.840523000	-0.750543000	-0.941609000
1	0.549640000	-2.945072000	-1.897428000
1	1.410911000	-2.084191000	-3.202367000
1	2.312267000	-3.153046000	-2.092852000
1	2.607406000	-0.467150000	1.684928000
1	3.258813000	0.041053000	0.126760000
1	-1.075392000	2.192653000	-0.770056000
1	-3.376121000	0.436324000	-0.097365000
1	-4.482611000	1.579600000	0.688797000
1	-3.561624000	2.095227000	-0.740269000
1	0.092159000	3.509543000	1.656483000
1	0.383722000	3.916557000	-0.030185000
1	-0.016389000	-3.572402000	0.631874000
8	0.406157000	-4.449232000	0.540506000
6	0.430638000	-5.068080000	1.826567000
1	-0.583787000	-5.277701000	2.191288000
1	0.962111000	-6.016305000	1.716017000
1	0.957877000	-4.451587000	2.567120000

$TS_{IIb'-IIc}$

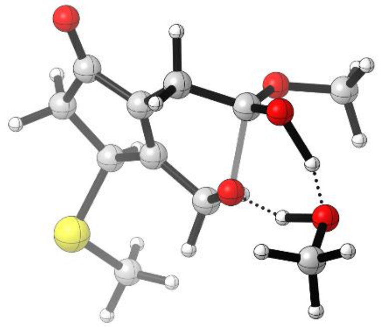

SCF energy = -1204.363146 Hartree

Thermal correction to Gibbs Free Energy = 0.232761990

Im.Freq= 1285.4366 icm^{-1}

8	2.769668000	2.661484000	0.800548000
6	1.654028000	2.184047000	0.693431000
6	1.314267000	0.749043000	0.624456000
6	-0.007115000	0.593746000	0.409621000
6	-0.659190000	-0.750406000	0.264831000
8	0.230065000	-1.756656000	0.677063000
6	1.945063000	-1.593932000	-0.025418000
8	2.464105000	-2.723846000	0.295093000
1	1.574314000	-3.612973000	0.322950000
8	1.697541000	-1.311407000	-1.337387000
6	1.599200000	-2.427054000	-2.228825000
6	2.344115000	-0.338953000	0.724837000
6	-0.734661000	1.916508000	0.263208000
16	-2.237705000	2.112625000	1.332941000
6	-3.548927000	1.471766000	0.231486000
6	0.341165000	2.971204000	0.605626000
1	-1.564226000	-0.789242000	0.890309000
1	-0.985670000	-0.878217000	-0.784481000
1	0.730737000	-3.050366000	-1.989461000
1	1.479229000	-1.995499000	-3.223613000
1	2.504880000	-3.037905000	-2.186229000
1	2.523698000	-0.622709000	1.766395000
1	3.296762000	0.028662000	0.322722000
1	-1.086921000	2.040718000	-0.768889000
1	-3.440039000	0.401929000	0.032277000
1	-4.497116000	1.634117000	0.751127000
1	-3.570260000	2.022580000	-0.714056000
1	0.151172000	3.445583000	1.575737000
1	0.411183000	3.774721000	-0.133213000
1	0.091720000	-3.043919000	0.545707000
8	0.511762000	-4.096558000	0.462163000
6	0.436667000	-4.752171000	1.737269000
1	-0.600491000	-5.043015000	1.925634000
1	1.059653000	-5.649847000	1.706476000
1	0.784612000	-4.090418000	2.538690000

IIc

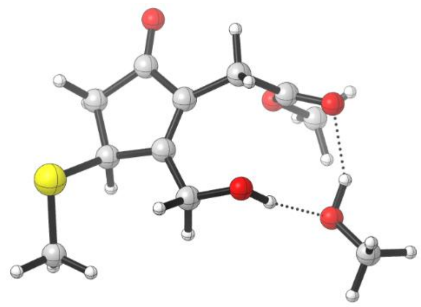

SCF energy = -1204.410505 Hartree
Thermal correction to Gibbs Free Energy = 0.234465900

8	2.663522000	2.352838000	-0.340806000
6	1.542056000	1.941039000	-0.102494000
6	1.210512000	0.669086000	0.580092000
6	-0.130684000	0.524649000	0.668385000
6	-0.921180000	-0.599864000	1.285146000
8	-0.157924000	-1.644497000	1.838843000
6	2.536105000	-1.441820000	0.131195000
8	2.818626000	-2.560386000	0.531195000
1	1.336539000	-3.697942000	0.358171000
8	2.389533000	-1.145685000	-1.168894000
6	2.566484000	-2.235764000	-2.093280000
6	2.330059000	-0.234370000	1.024961000
6	-0.873934000	1.688191000	0.020927000
16	-2.095108000	2.559535000	1.118338000
6	-3.636545000	1.636360000	0.780005000
6	0.235144000	2.659242000	-0.430392000
1	-1.525191000	-0.163027000	2.094212000
1	-1.642648000	-0.969400000	0.534865000
1	1.786681000	-2.987094000	-1.941670000
1	2.481505000	-1.789949000	-3.084267000
1	3.548952000	-2.695010000	-1.959924000
1	2.184200000	-0.609763000	2.036865000
1	3.256319000	0.356086000	0.986054000
1	-1.455772000	1.323208000	-0.835149000
1	-3.610842000	0.617905000	1.176529000
1	-4.438481000	2.185490000	1.280959000
1	-3.849789000	1.611132000	-0.293294000
1	0.204502000	3.611409000	0.110822000
1	0.182070000	2.900190000	-1.496675000
1	-0.069921000	-2.373594000	1.186076000
8	0.380173000	-3.842395000	0.208282000
6	-0.012989000	-5.051070000	0.856609000
1	-1.081593000	-5.183647000	0.669782000
1	0.522921000	-5.917513000	0.447263000
1	0.152019000	-5.008362000	1.941380000

Biological studies

Biological studies

Plasmodium falciparumin vitro **culture:** Laboratory-adapted *Plasmodium falciparum* line 3D7-GFP (MRA-1029, MR4. ATCC® Manassas Virginia; constitutively expressing Green Fluorescence Protein, chloroquine and artemisinin susceptible) was continuously cultivated at 5% hematocrit, 37°C and atmosphere with 5% of CO_2, human serum was replaced with 0.5% AlbuMAXII (Invitrogen™) in the culture medium (RPMI1640, Invitrogen™).

Antimalarial activity primary screening: All compounds were screened for *in vitro* antimalarial activity. Unsynchronized culture with 2% hematocrit and 1% parasitemia, was incubated in a 96-well flat-bottom plate with 1uM and 10uM of each compound for 72h (37 °C and 5% CO2). Each plate also included growth controls wells: no drug added and 5nM and 500 nM of chloroquine. Parasite growth was assessed by flow cytometry (Beckman Coulter, Cytoflex) with a 96-well plate reader, using Fl-1 (green fluorescent protein [GFP]; excitation wavelength, 488 nm). Typically, 20,000 to 40,000 RBCs were counted for each well. Samples were analyzed using FlowJo software (Tree Star Inc.).[7]

Half maximal inhibitory concentration (IC_{50}) Assay:
Compounds exhibiting over 70% growth inhibition in the primary screening assay, were selected as active and confirmed in the dose-response assay to estimate the correspondent IC_{50}. Unsynchronized culture with 2% hematocrit and 1% parasitemia, was incubated with the tested compounds in 3-fold serial dilutions ranging from 10 to 0.014 µM. After 72h at 37 °C and 5% CO_2, parasite growth was assessed by flow cytometry as described above. Half-maximal inhibitory concentrations (IC_{50}) were determined with GraphPad Prism 5 (trial version). At least three experiments, each in duplicate, were performed to obtain the mean IC_{50} presented.[7]

name	Inhibition rate (%) at 10 µM		Inhibition rate (%) at 1 µM		IC_{50} (µM)	
	mean	SD	mean	SD	mean	SD
**3b**	9.72	3.77	0.00	0.00	n.d	n.d
**3h**	4.93	2.33	0.00	0.00	n.d	n.d
**3l**	85.5	1.9	18.8	6.9	n.d	n.d
**3m**	78.1	1.8	18.7	3.7	n.d	n.d
**4a**	91.4	6.7	91.3	1.6	4.367	±0.41
**4b**	68.98	18.60	2.88	1.32	4.18	±0.14
**4c**	98.6	0.2	95.1	1.3	1.319	±0.22

Anticancer Activity

The in vitro anticancer studies were carried out in Human breast cancer cells line (MCF-7), human colorectal cancer cells line (HT-29) and human lung cancer cells line (NCI-H460) by a Neutral red cell cytotoxicity assay, which is a common method based on the detection of viable cells via the uptake of the dye neutral red (a eurhodin dye that stains lysosomes in viable cells).

Cells were cultured in RPMI-1640 medium supplemented with 10% Fetal Bovine Serum (FBS) and antibiotic antimycotic solution in 75 cm^2 tissue culture flasks and incubated with a humidified 5% CO_2 atmosphere and at 37 ºC.

HT-29, NCI-H460 and MCF-7 human cancer cells were seeded in 96-well plates with RPMI-1640 (supplemented with 10%FBS and 1% antibiotic antimycotic solution) at different concentrations ($1x10^5$ cell/mL, $5x10^4$ cell/mL and $1,5x10^5$ cell/mL respectively) and incubated for 24h (in a humidified 5% CO_2 atmosphere at 37 ºC). After that, stock solutions of 20 µM for the corresponding cyclopentenones were prepared in culture medium with DMSO (≤0.5% (v/v)) and then incubated (in triplicate) with the cells for 48h. After incubation, the plate was treated with a medium containing Neutral Red (50 µg/mL) and incubated again for more 3h. Then, the medium was removed, the plate was washed with HBSS followed by the addition of an organic acid solution of 20 mL ethanol + 20 mL H_2O + 400 µL glacial acetic acid. Finally, absorbance was measured by spectrophotometry at 540 nm and the cells viability calculated.

Cytotoxicity against human health cells line

The in vitro cytotoxicity studies were carried out in human embryonic kidney 293T healthy cells line (HEK 293T) by a Neutral red cell cytotoxicity assay. Cells were cultured in DMEM medium supplemented with 10% FBS and antibiotic antimycotic solution (1%) in 75 cm^2 tissue culture flasks and incubated with a humidified 5% CO_2 atmosphere (at 37 ºC).

HEK 293T cells were seeded in a 96-well plate at a concentration of $7x10^4$ cell/mL (in DMEM with 10% FBS and antibiotic antimycotic solution) and were incubated for 24h (in a humidified 5% CO_2 atmosphere at 37 ºC). After that, the procedure was the same as performed previously: compounds **4a**, **4b** and **4c** were incubated for 48h, treated with Neutral Red and the absorbance measured.

name	Inhibition rate (%) at 10 µM (HEK-293T)		Inhibition rate (%) at 50 µM (HEK-293T)	
	mean	SD	mean	SD
**4a**	86.89	15.50	90.38	7.66
**4b**	>100	14.95	>100	19.39
**4c**	>100	967	91.45	17.65

Both cancer and healthy cell lines were purchased from the American Type Culture Collection (ATCC). Cell culture RPMI-1640 Medium, Dulbecco's Modified Eagle's Medium (DMEM),

Trypsin–EDTA solution, Stabilized Antibiotic Antimycotic solution (100x), and Hanks' Balanced Salt solution (HBSS) were purchased from Sigma. Fetal Bovine Serum (FBS) was purchased from VWR and Dimethyl Sulfoxide (DMSO) from Carlo Erba.

Copies of NMR spectra

^{1}H NMR (300 MHz, $CDCl_3$) and ^{13}C NMR (75 MHz, $CDCl_3$) of compound **3a**

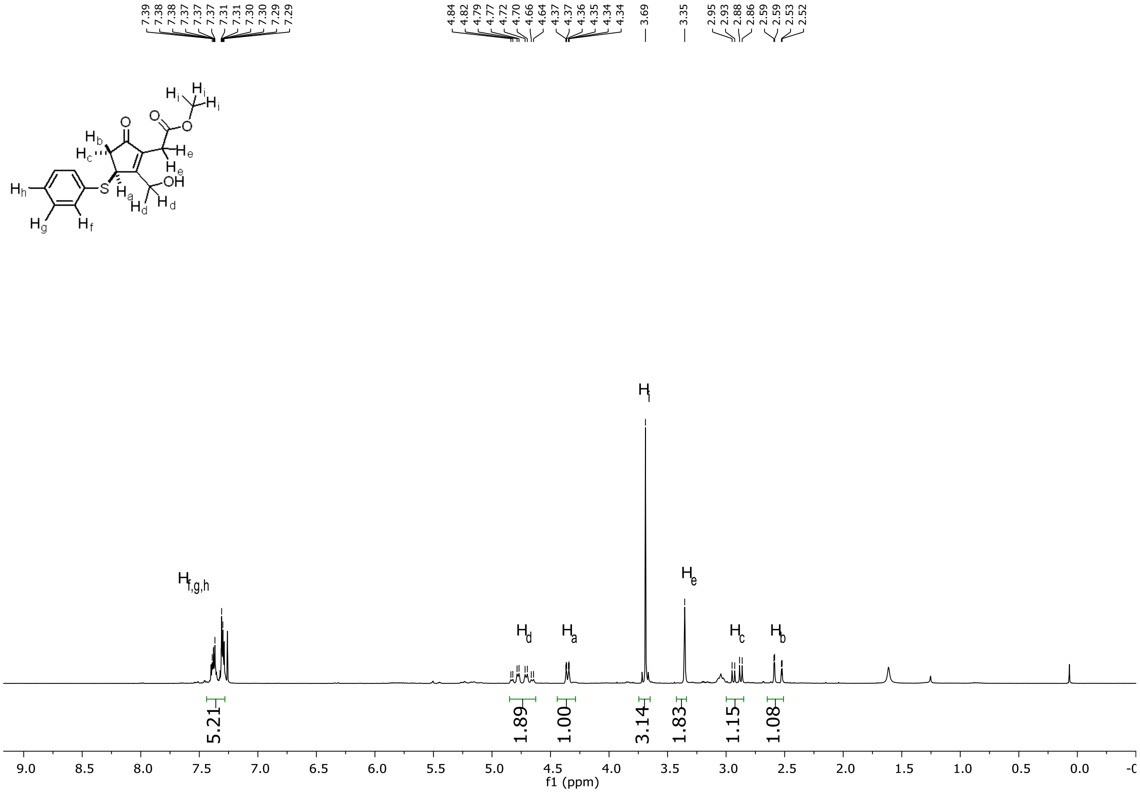

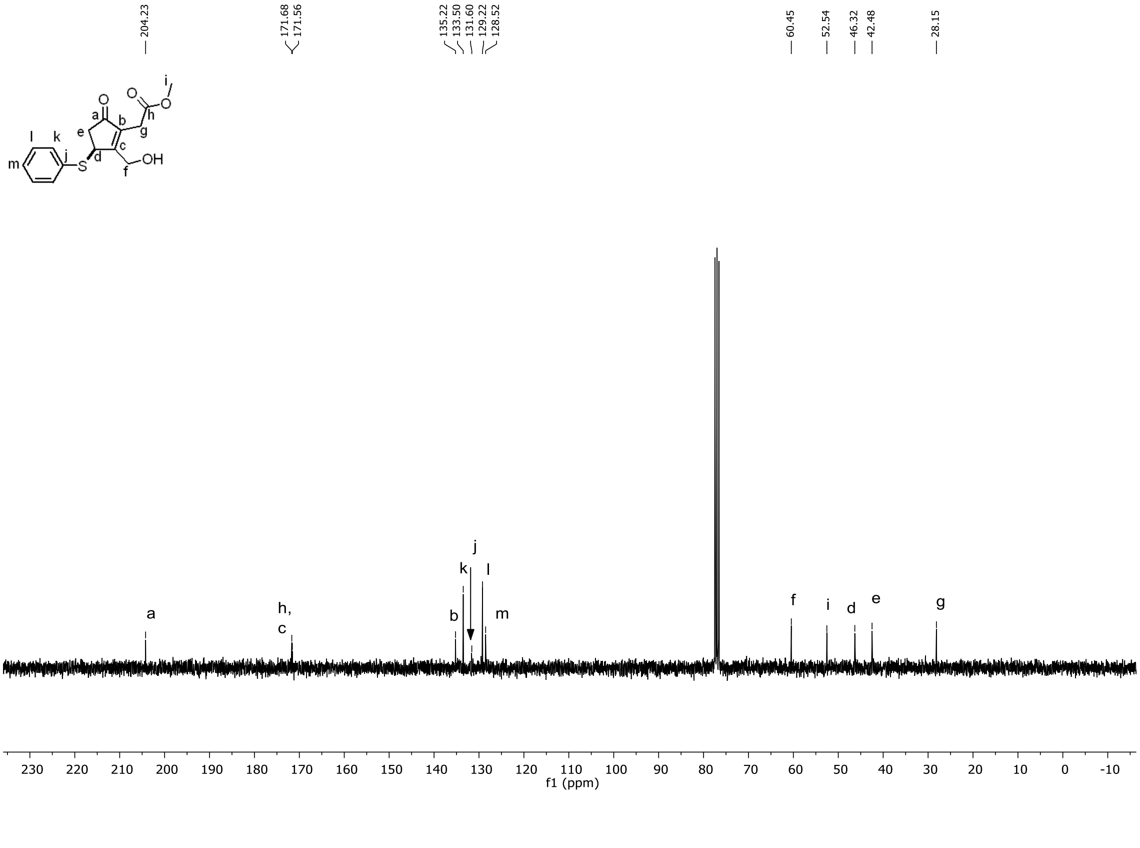

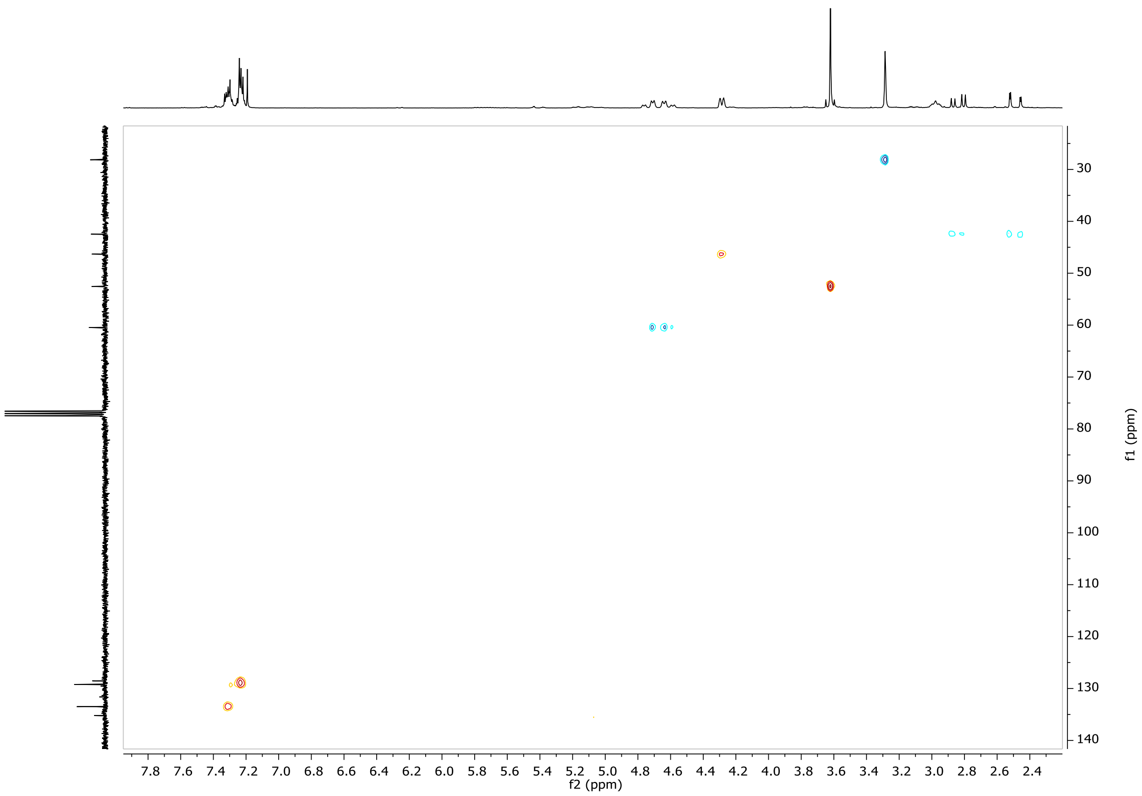

HSQC of compound **3a**

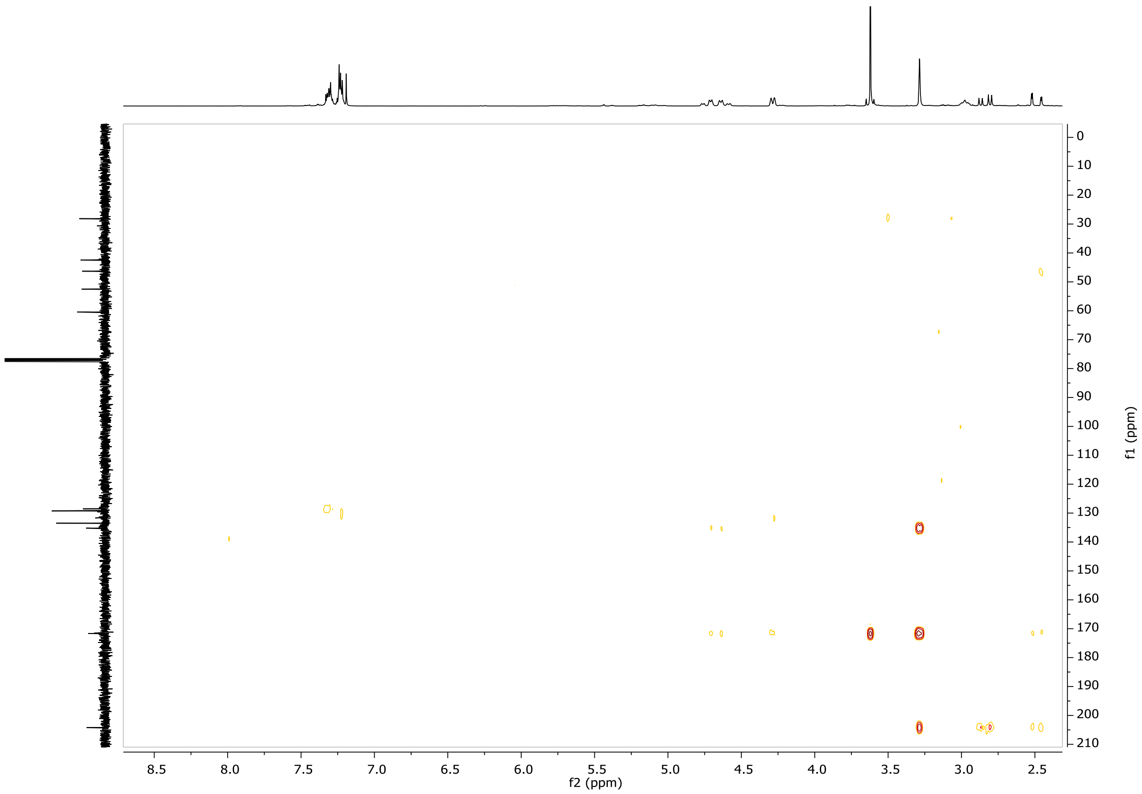

HMBC of compound **3a**

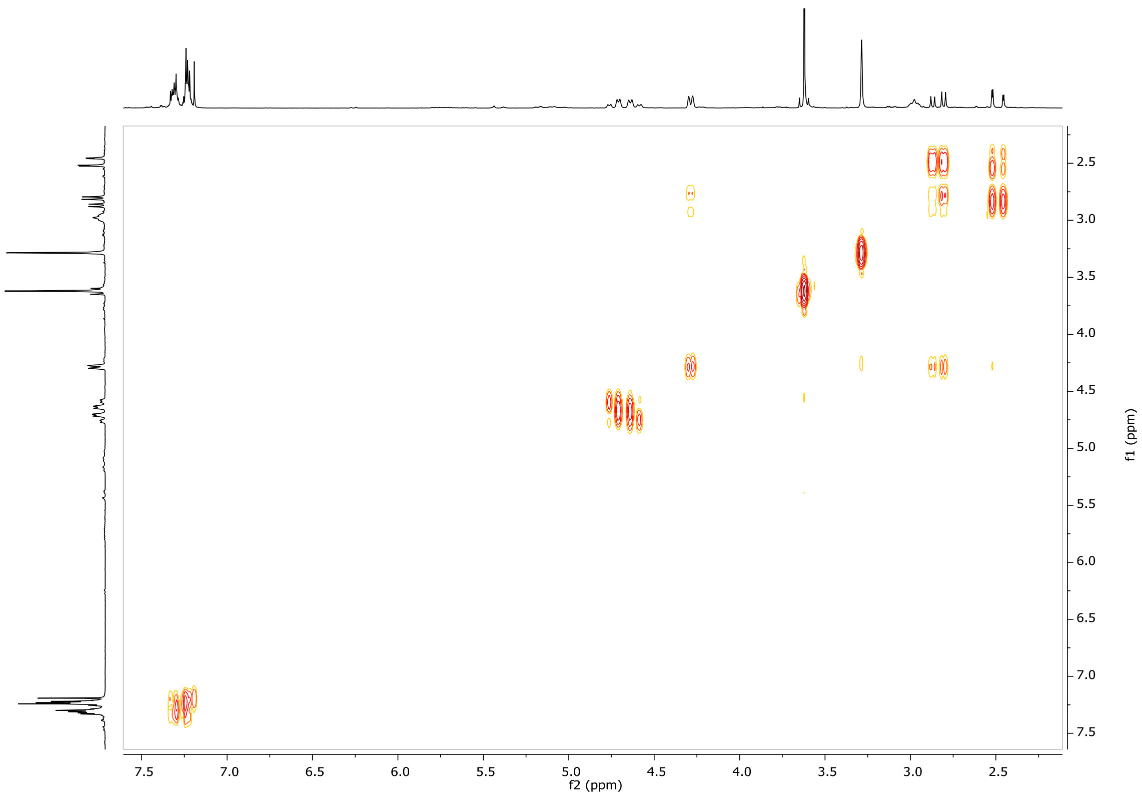

COSY of compound **3a**

^{1}H NMR (300 MHz, $CDCl_3$) and ^{13}C NMR (75 MHz, $CDCl_3$) of compound **3b**

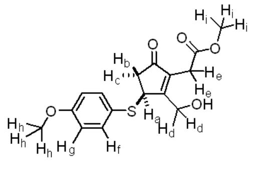

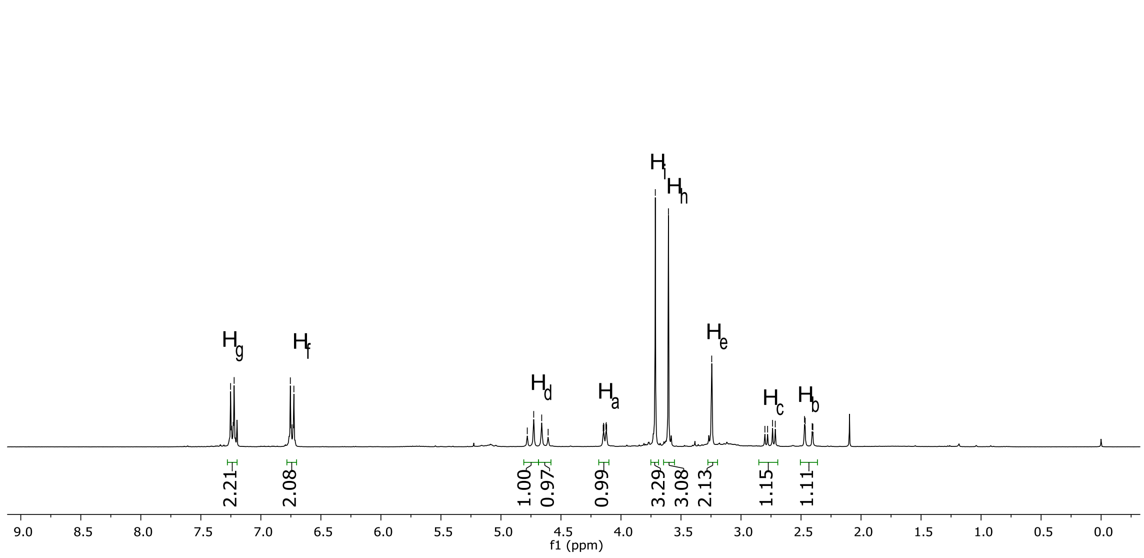

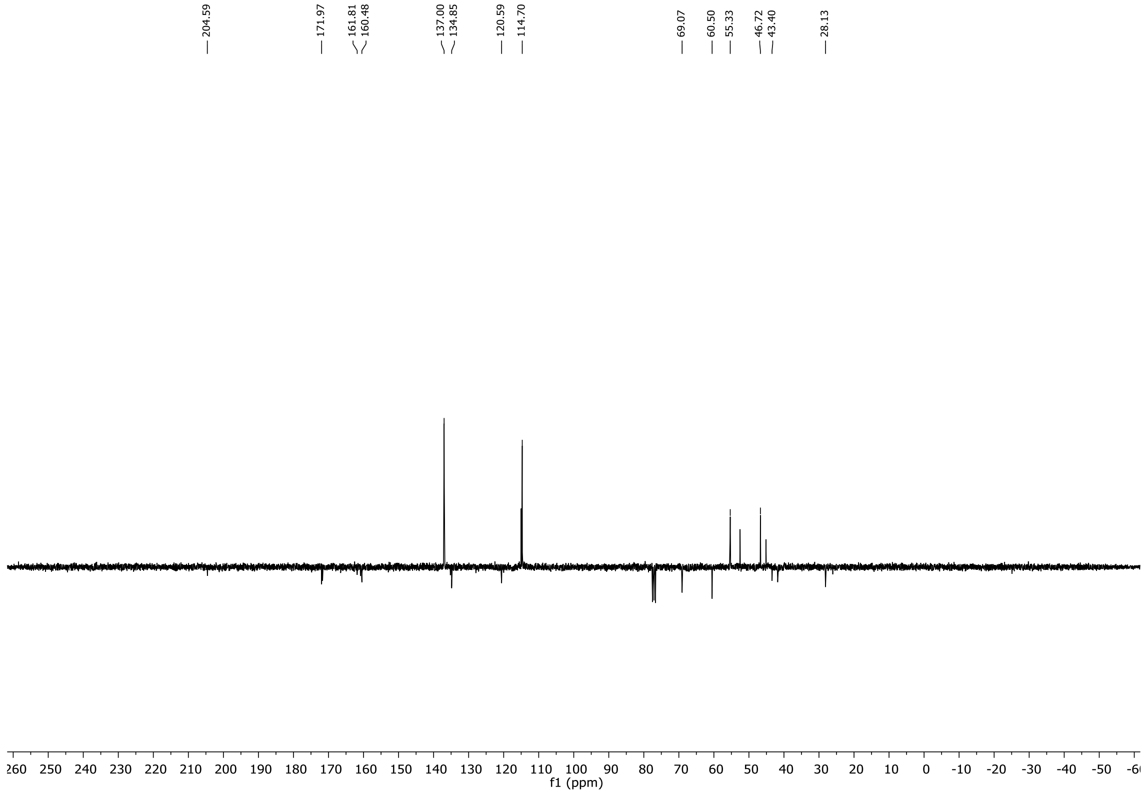

^{1}H NMR (300 MHz, $CDCl_3$) and ^{13}C NMR (75 MHz, $CDCl_3$) of compound **3c**

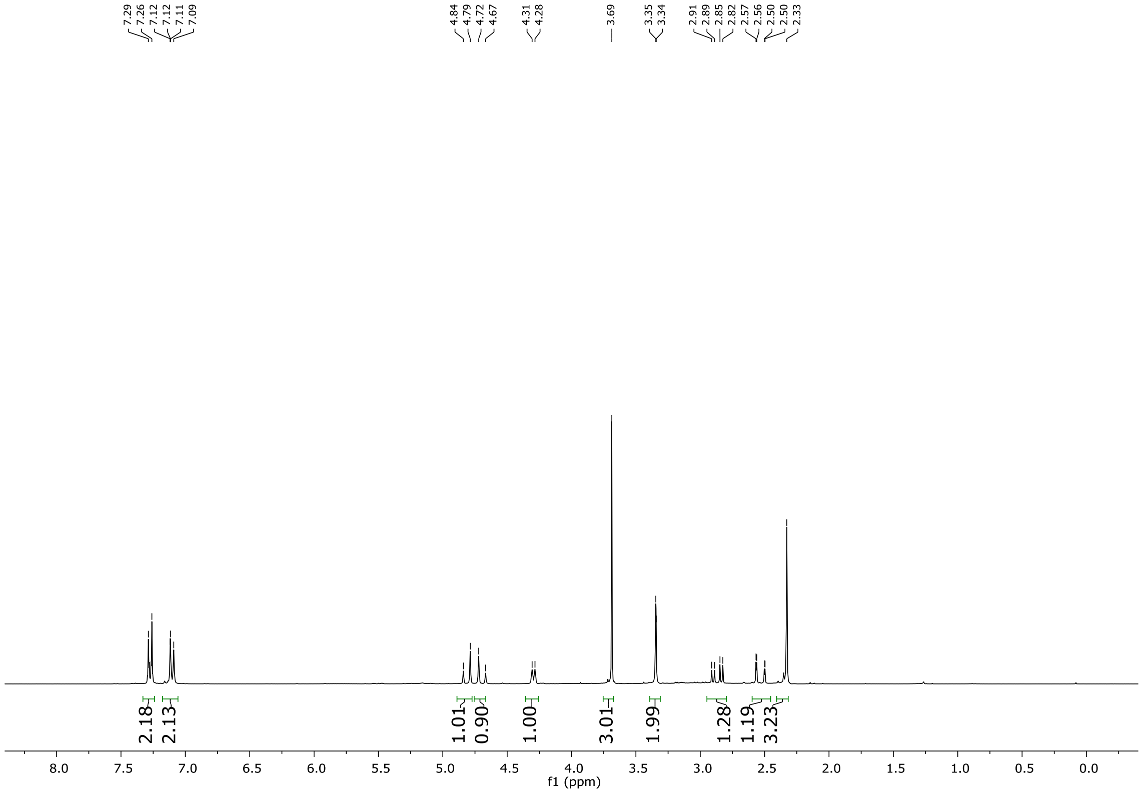

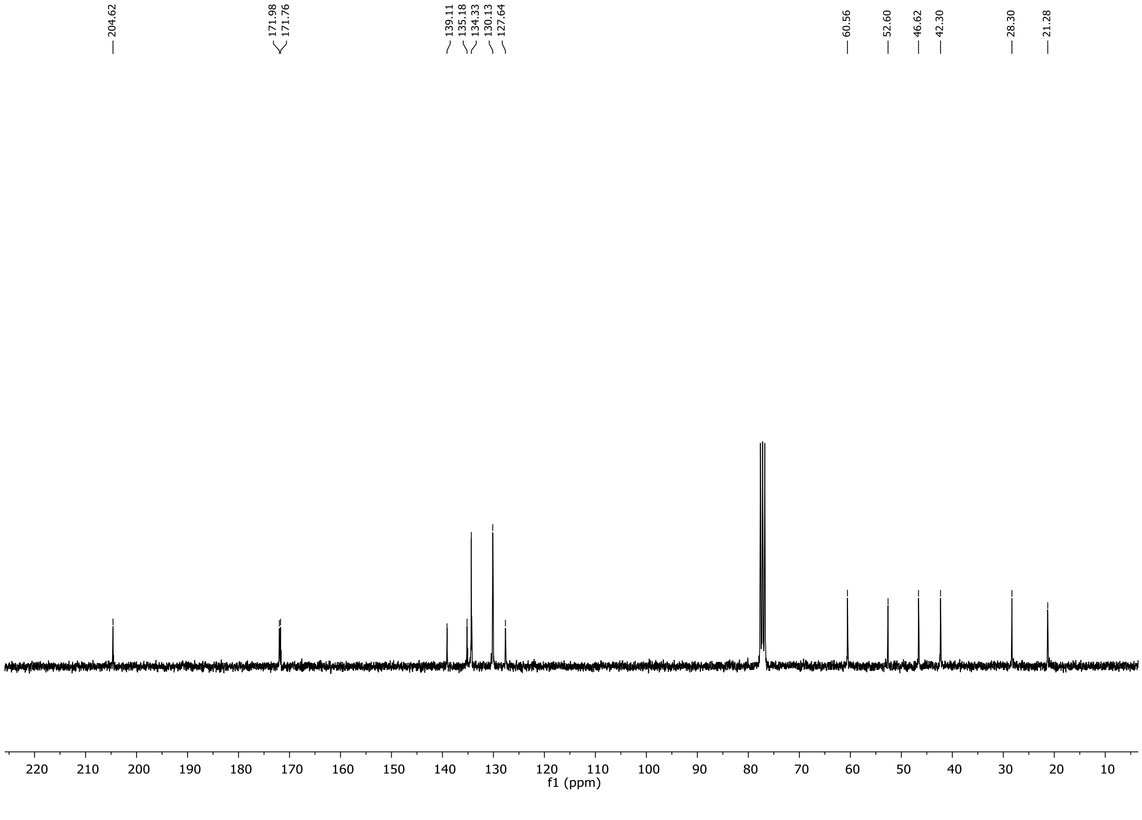

^{1}H NMR (300 MHz, $CDCl_3$) and ^{13}C NMR (75 MHz, $CDCl_3$) of compound **3d**

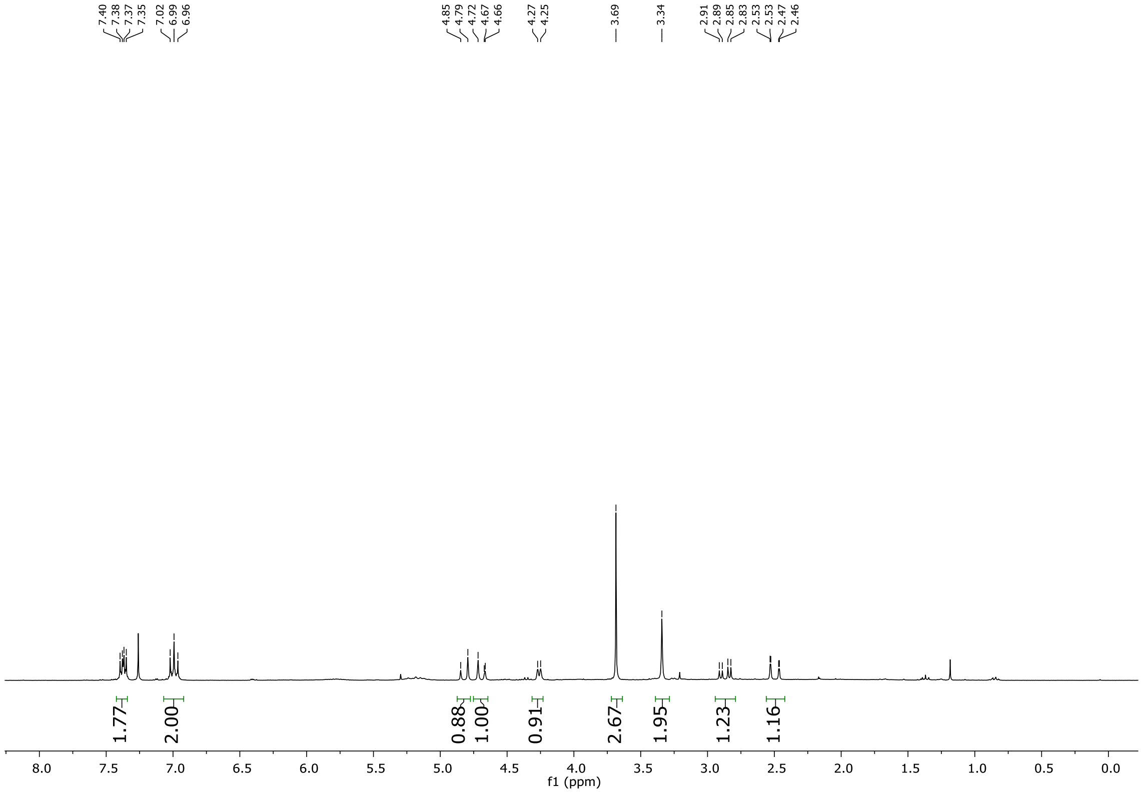

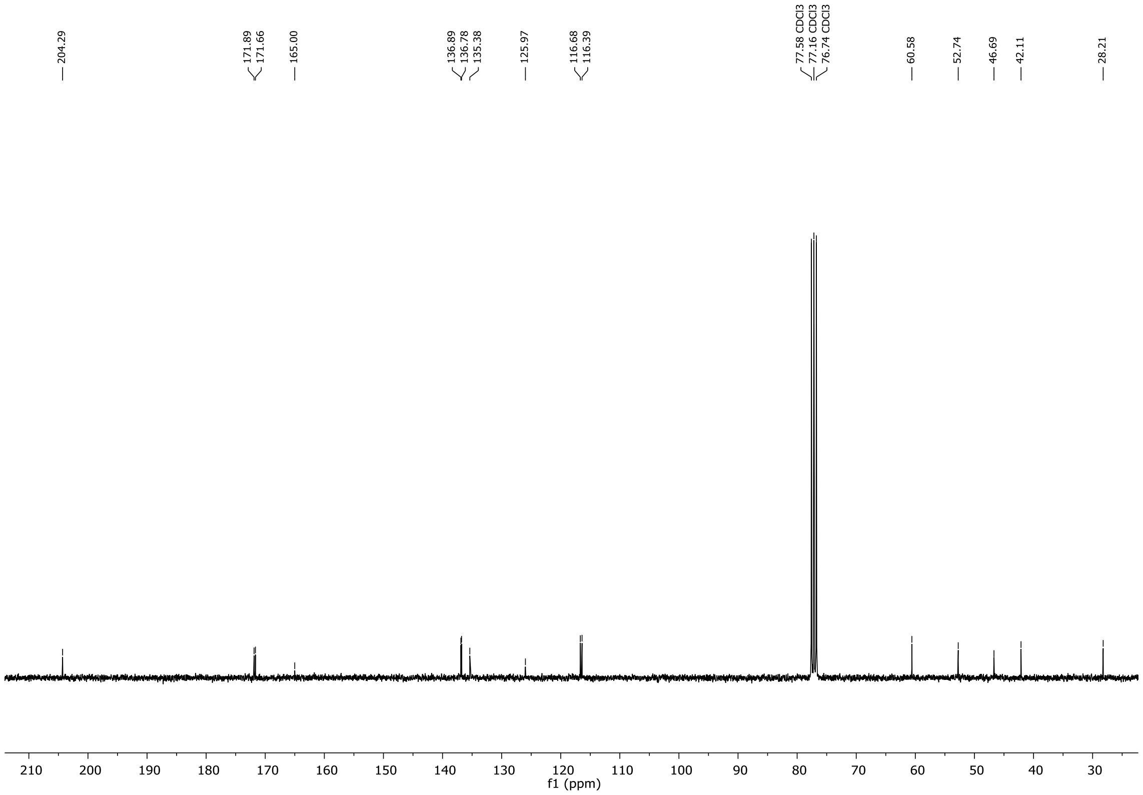

^{1}H NMR (300 MHz, $CDCl_3$) and ^{13}C NMR (75 MHz, $CDCl_3$) of compound **3e**

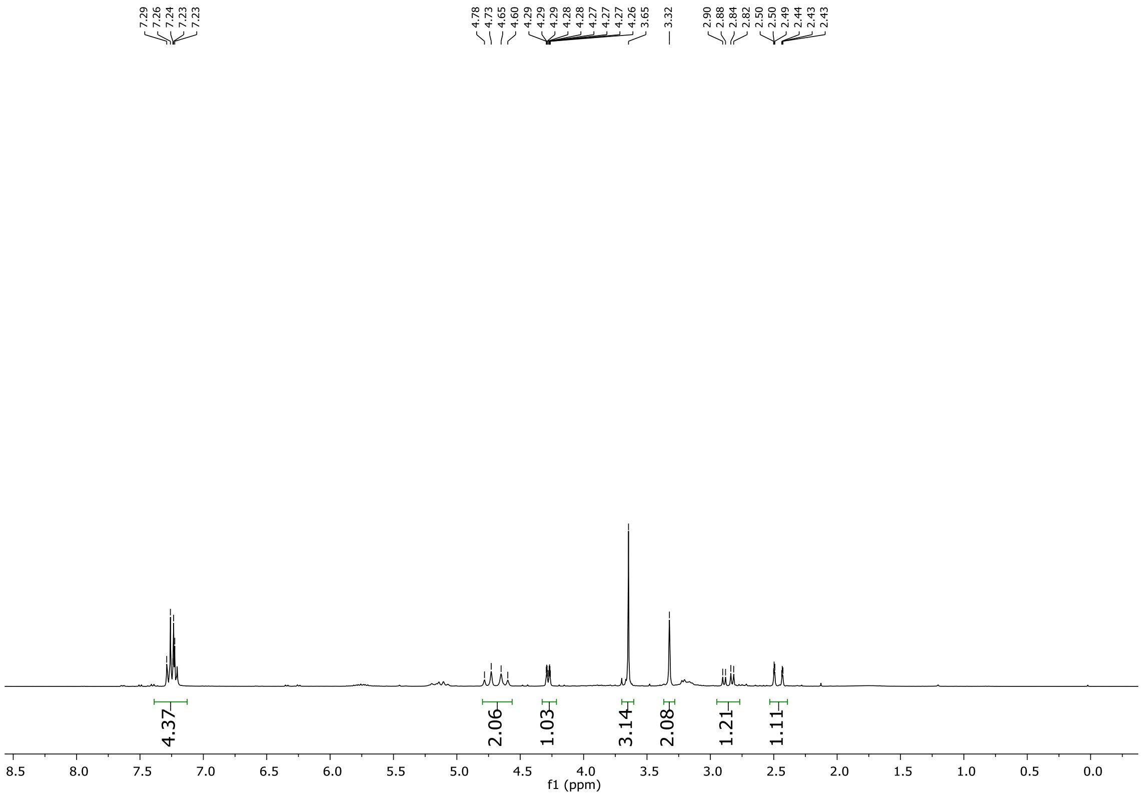

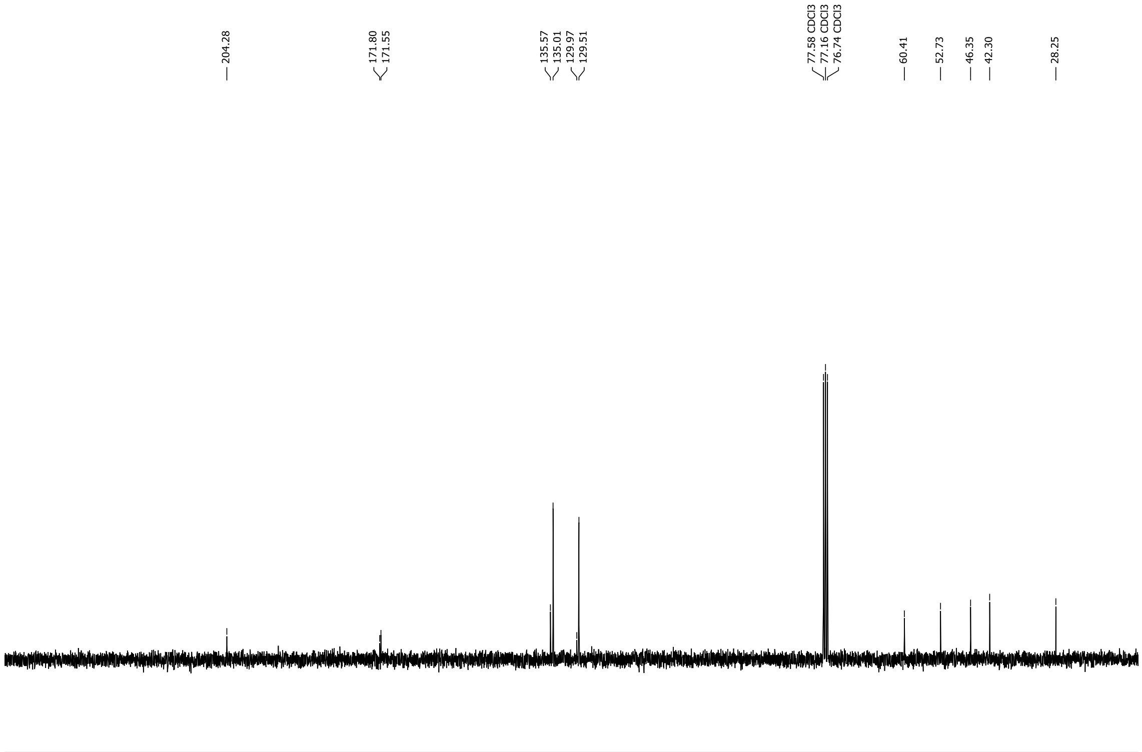

^1H NMR (300 MHz, $CDCl_3$) and ^{13}C NMR (75 MHz, $CDCl_3$) of compound **3f**

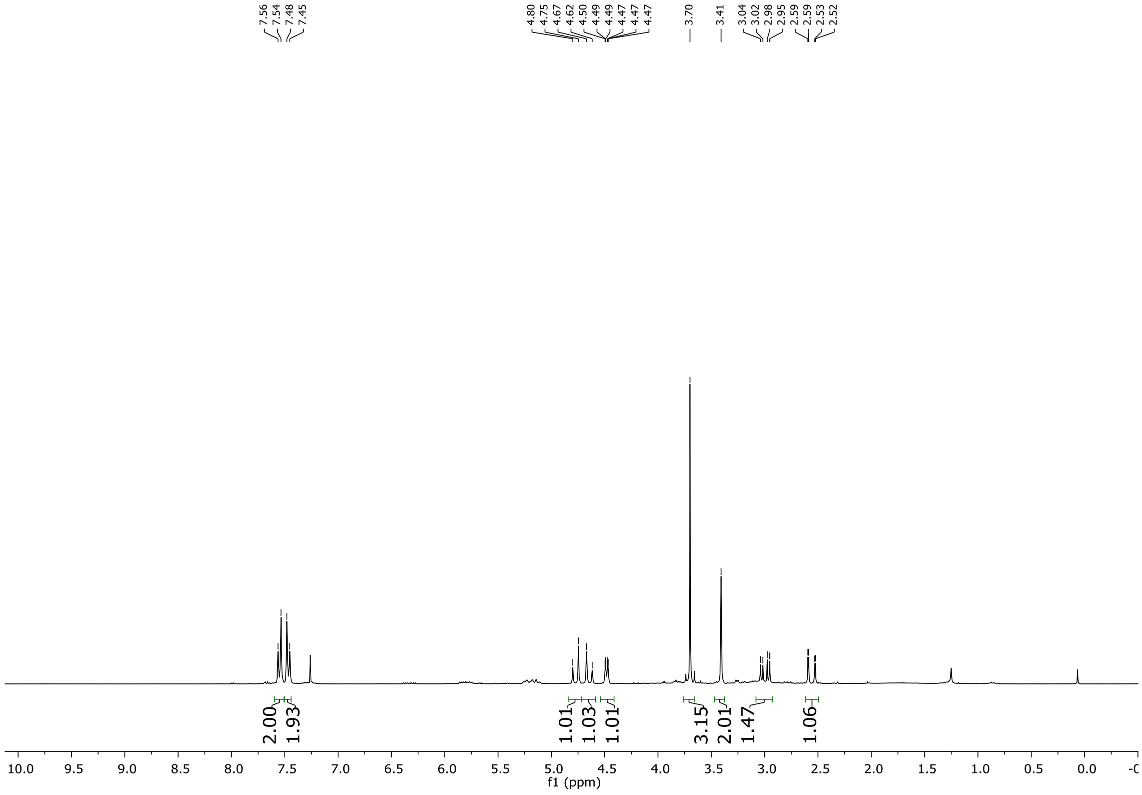

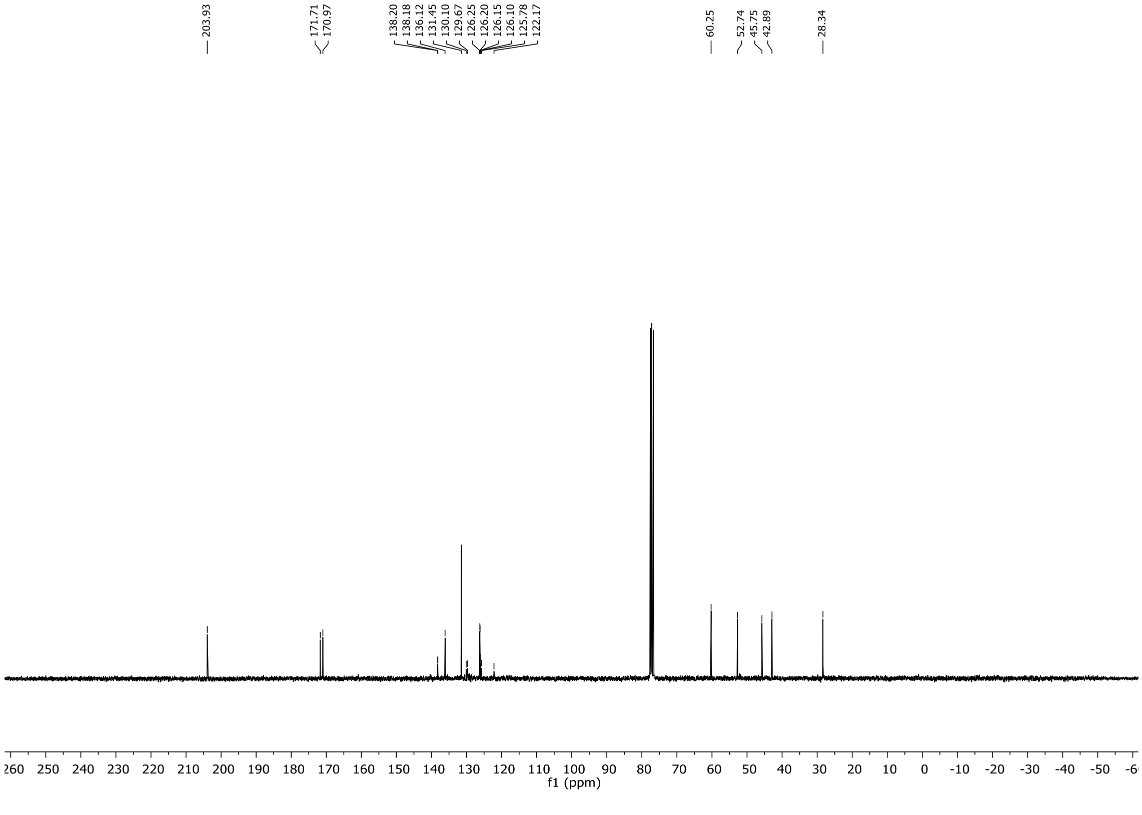

^{1}H NMR (300 MHz, $CDCl_3$) and ^{13}C NMR (75 MHz, $CDCl_3$) of compound **3g**

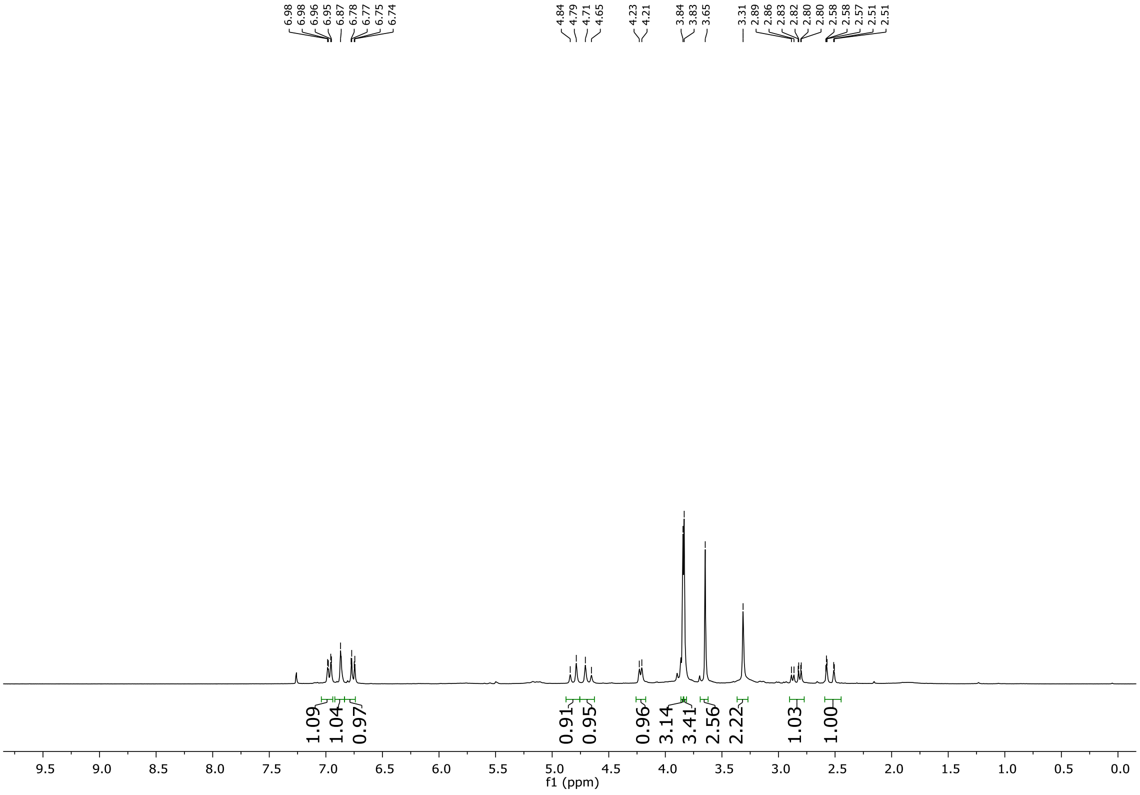

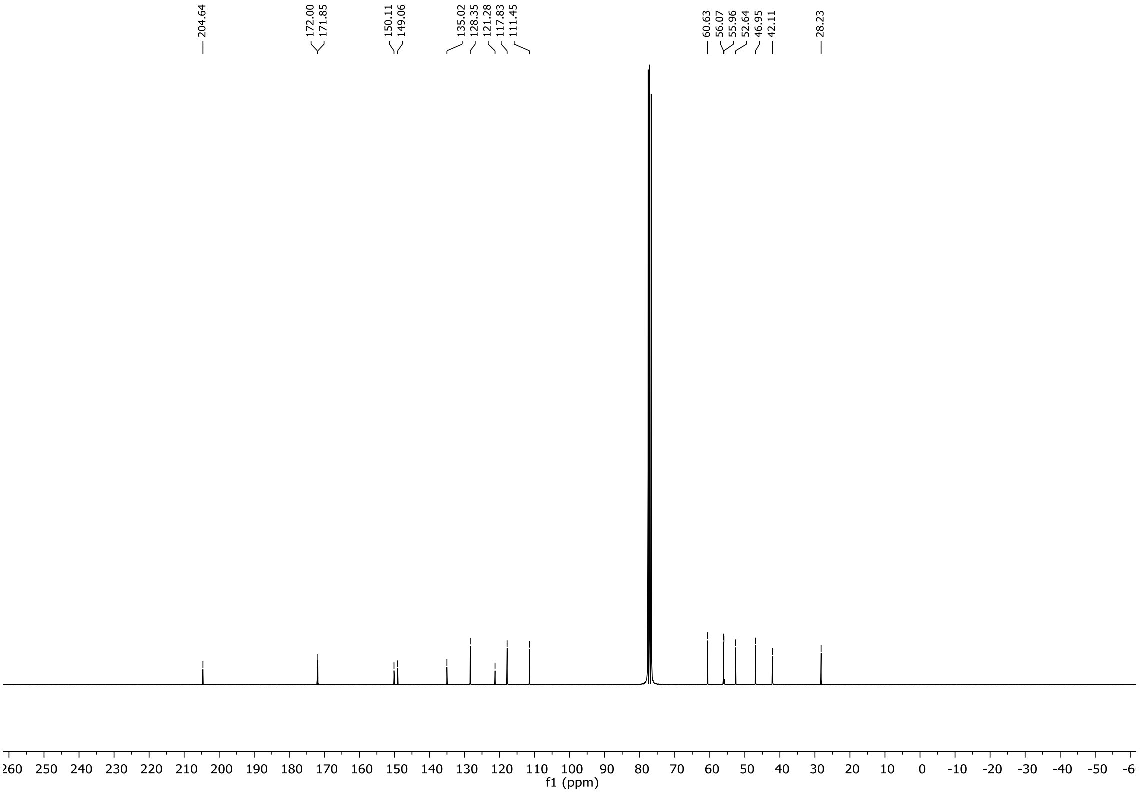

^{1}H NMR (300 MHz, $CDCl_3$) and ^{13}C NMR (75 MHz, $CDCl_3$) of compound **3h**

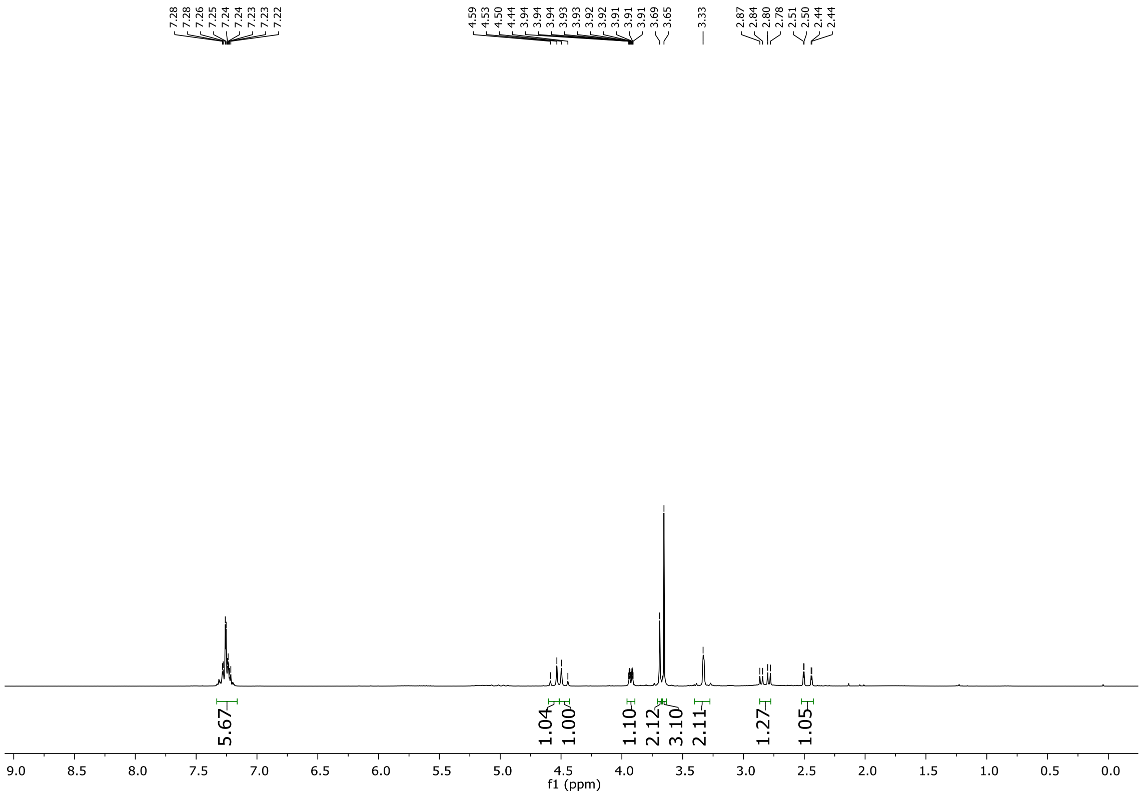

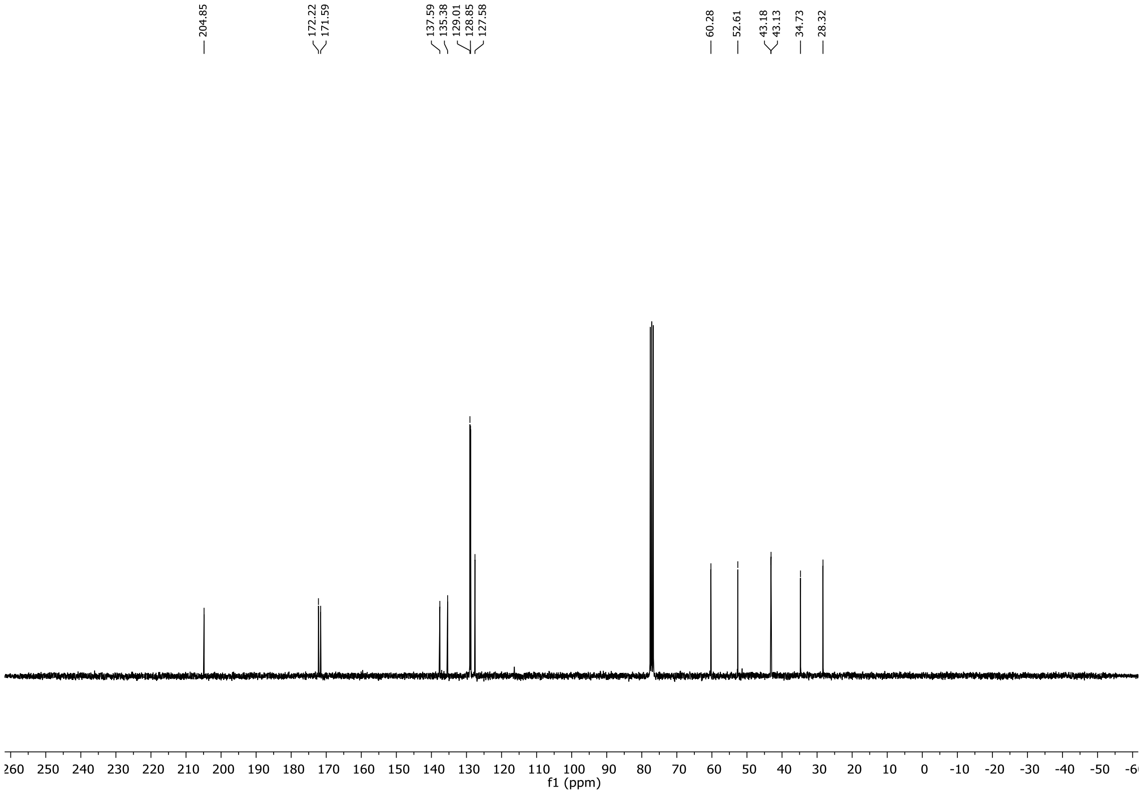

^{1}H NMR (300 MHz, $CDCl_3$) and ^{13}C NMR (75 MHz, $CDCl_3$) of compound **3i**

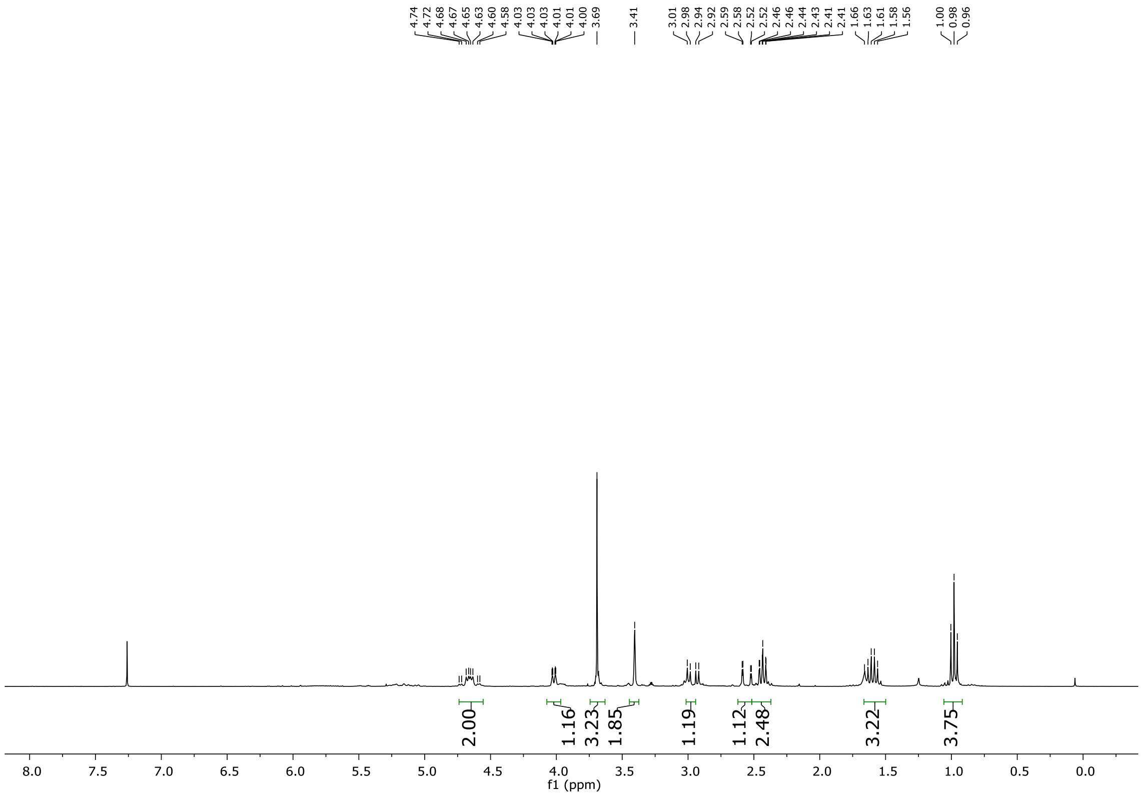

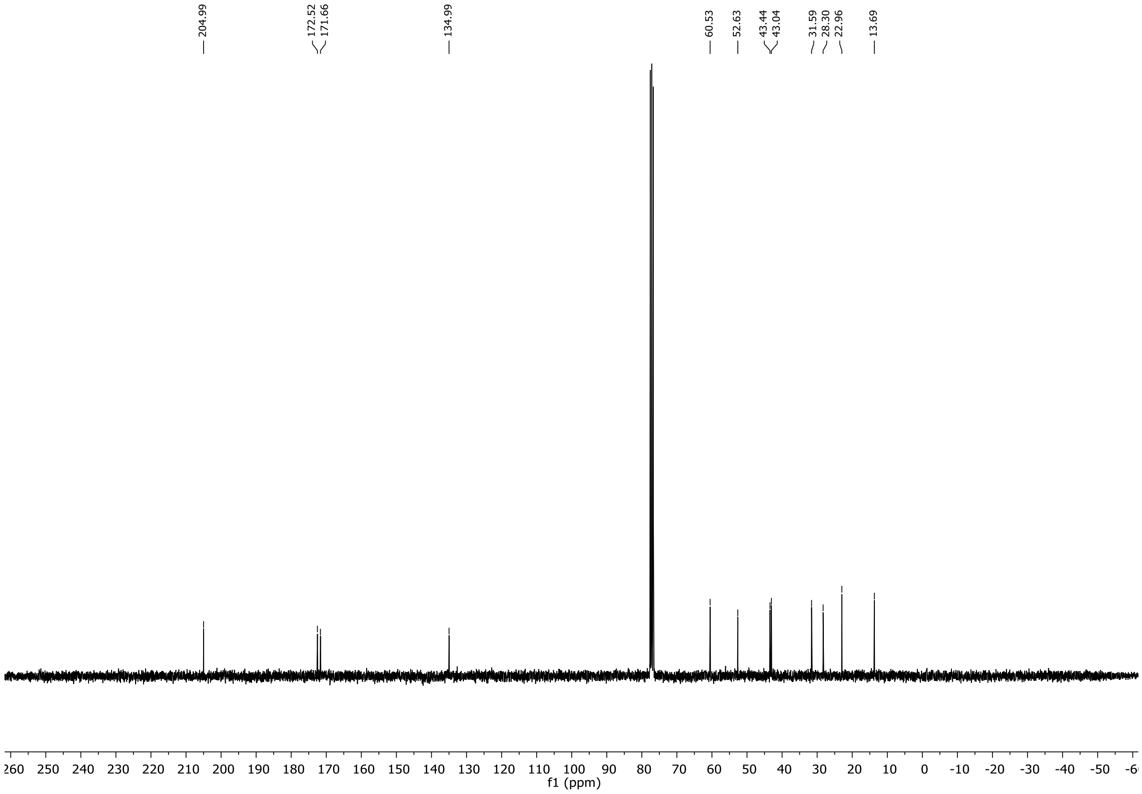

^{1}H NMR (300 MHz, $CDCl_3$) and ^{13}C NMR (75 MHz, $CDCl_3$) of compound **3j**

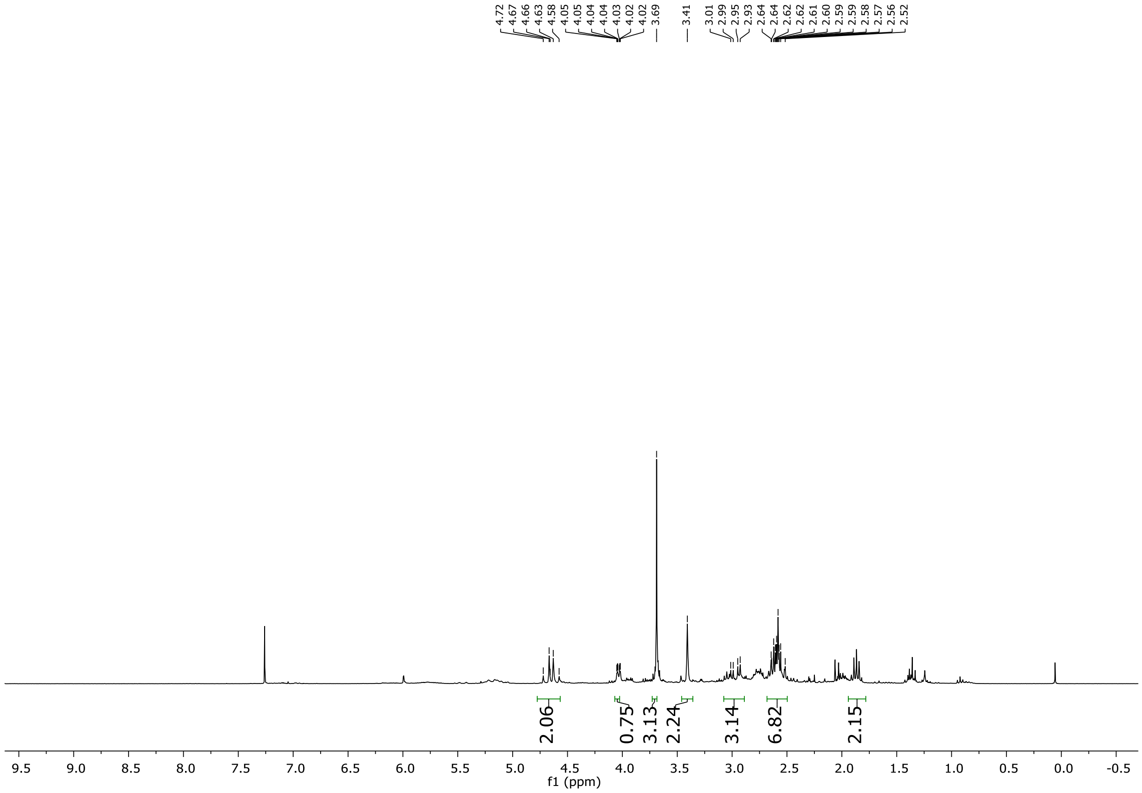

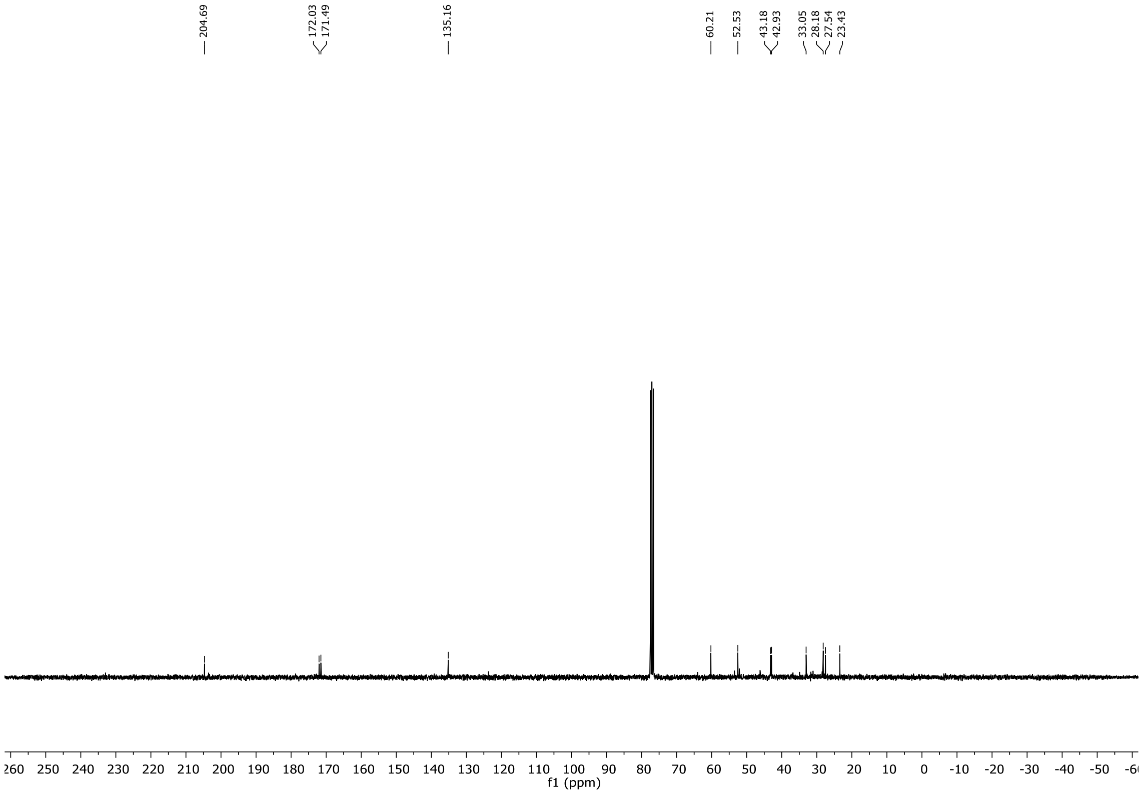

^{1}H NMR (300 MHz, $CDCl_3$) and ^{13}C NMR (75 MHz, $CDCl_3$) of compound **3k**

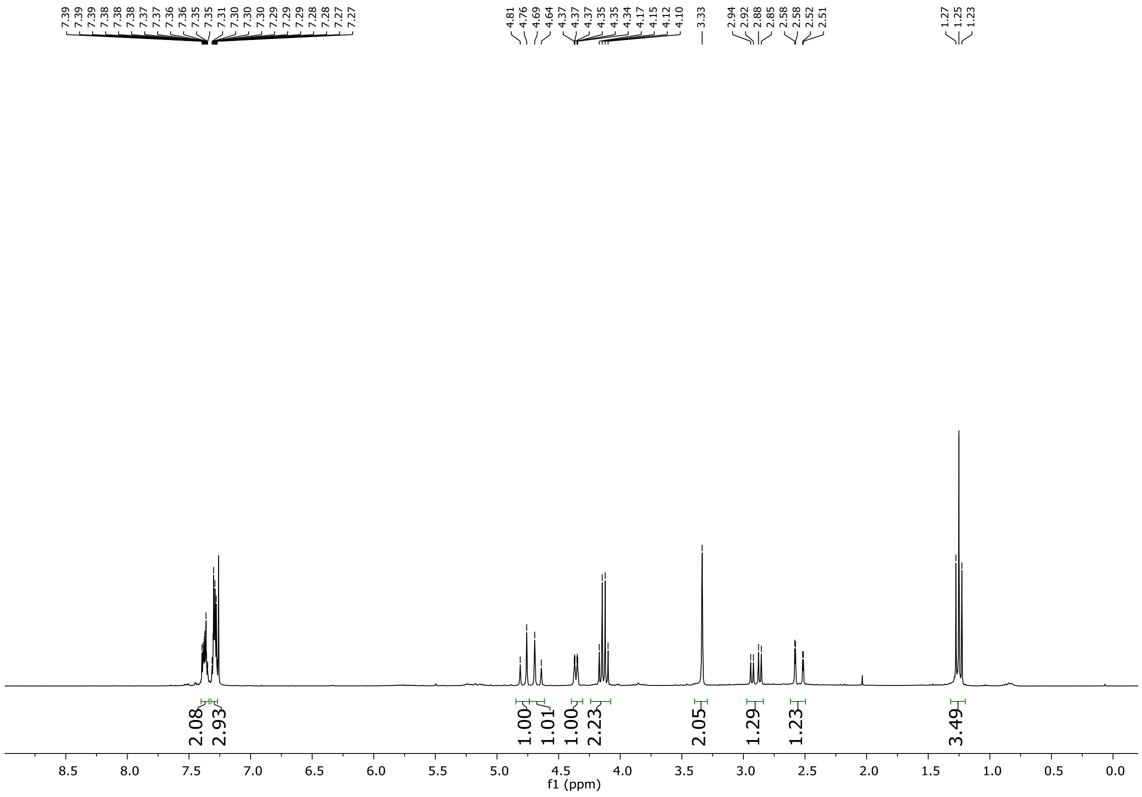

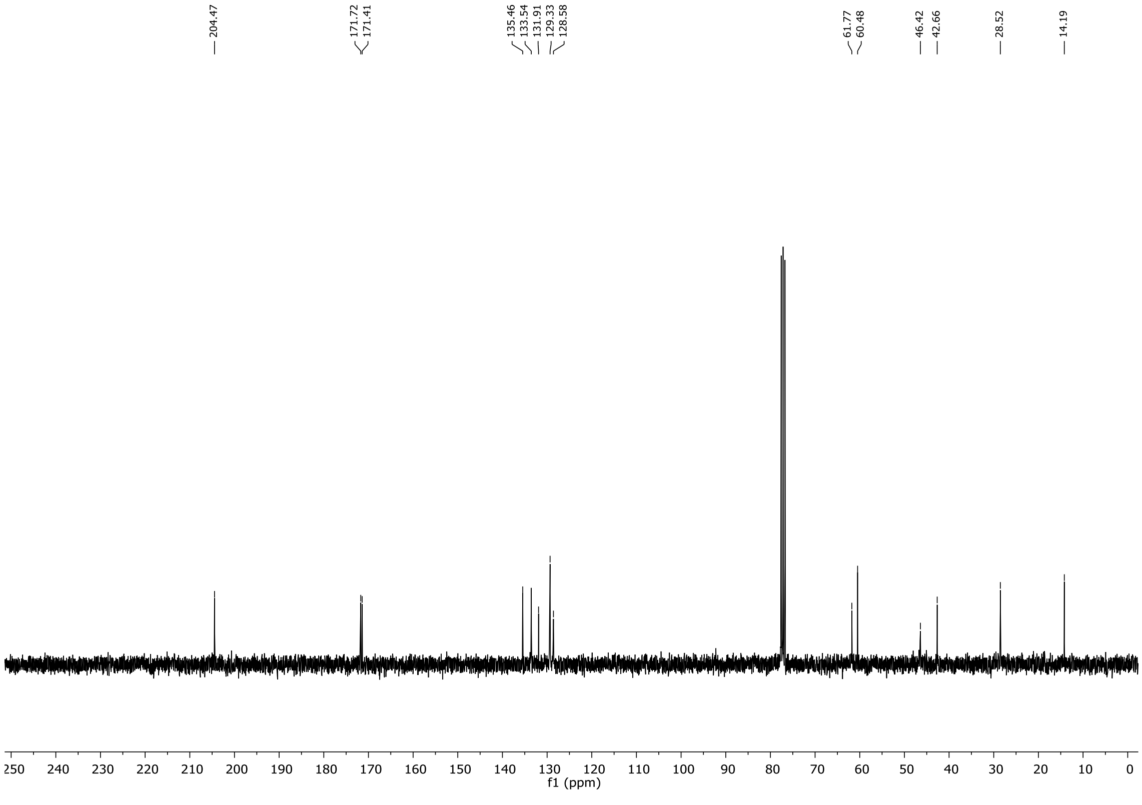

^{1}H NMR (300 MHz, $CDCl_3$) and APT NMR (75 MHz, $CDCl_3$) of compound **3l**

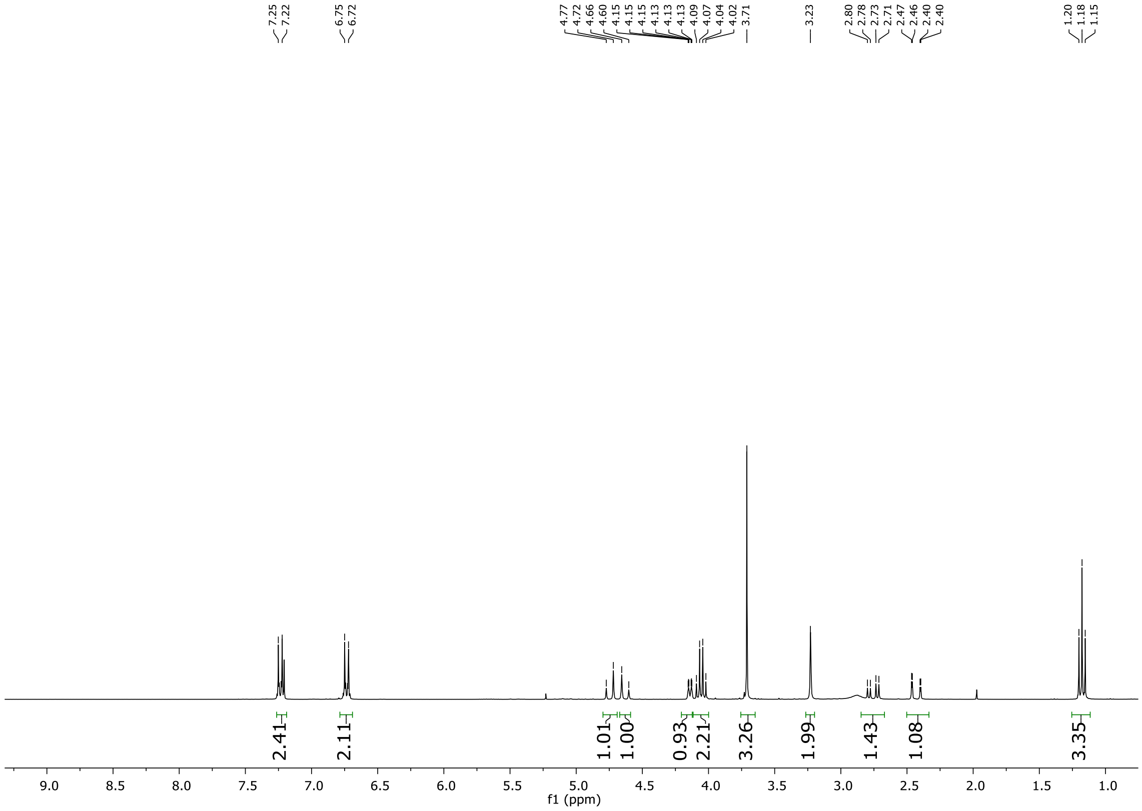

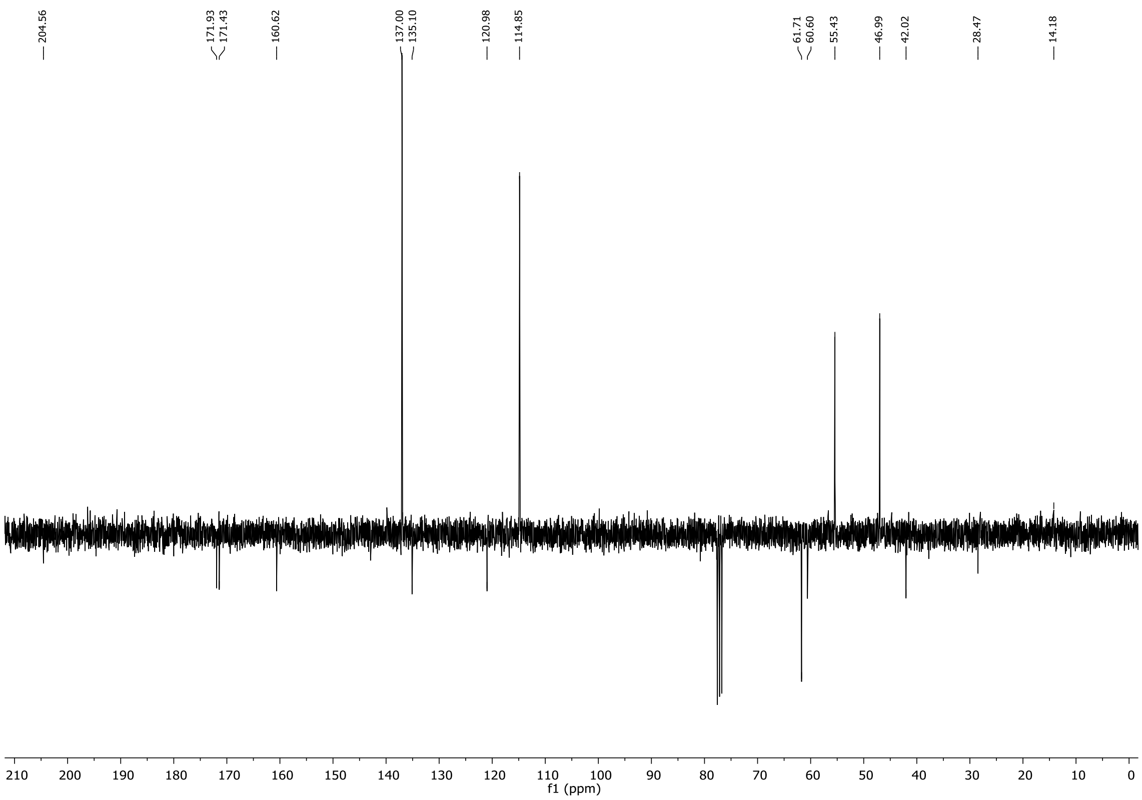

^{1}H NMR (300 MHz, D_2O) and ^{13}C NMR (75 MHz, D_2O) of compound **3m**

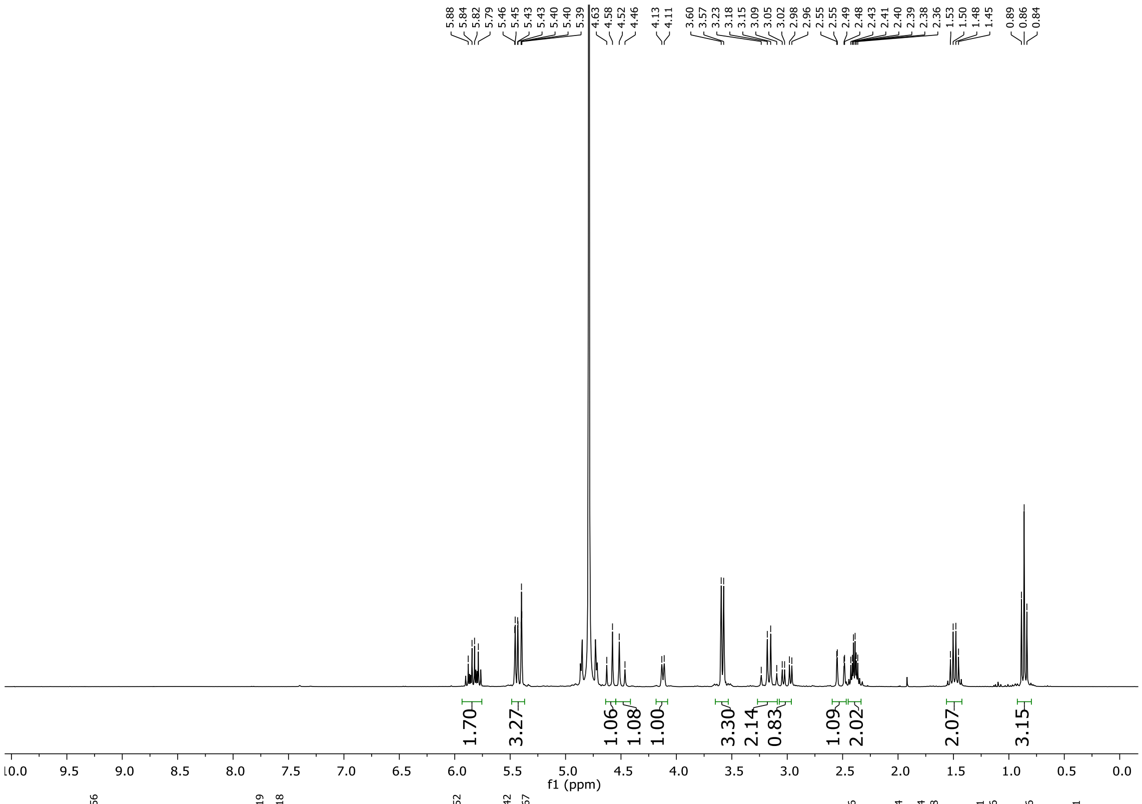

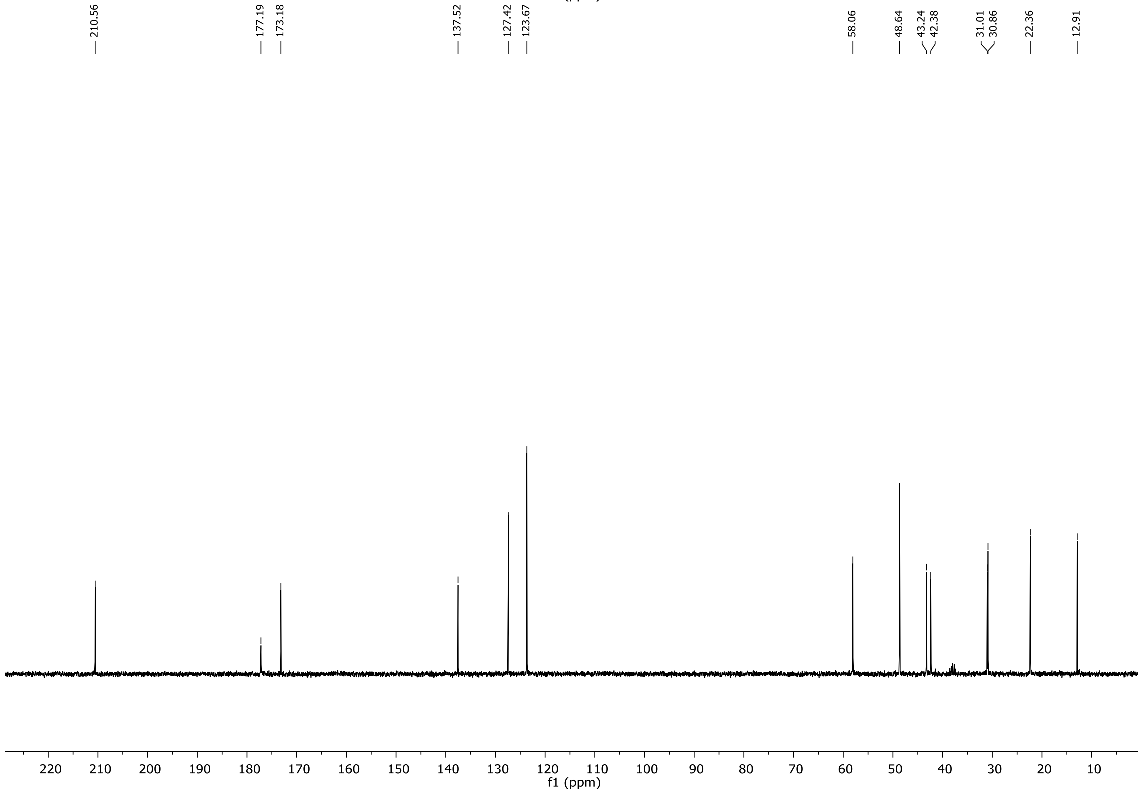

^{1}H NMR (300 MHz, D_2O) and ^{13}C NMR (75 MHz, D_2O) of compound **3n**

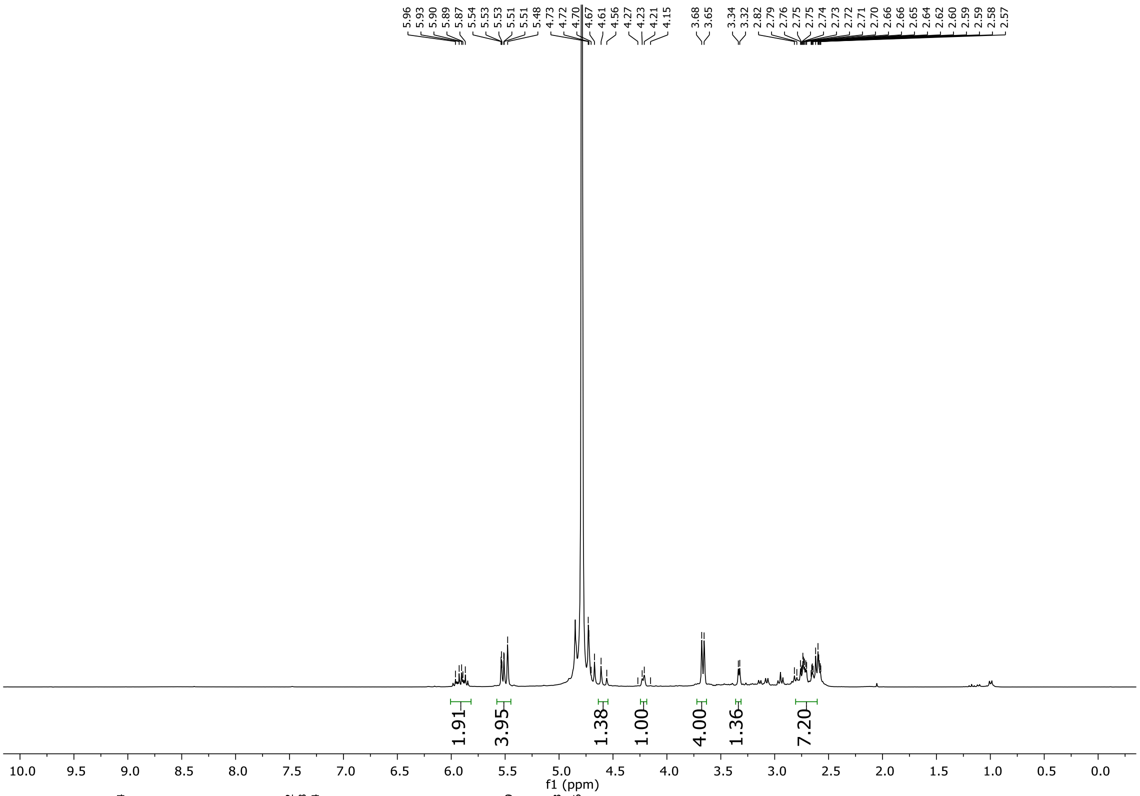

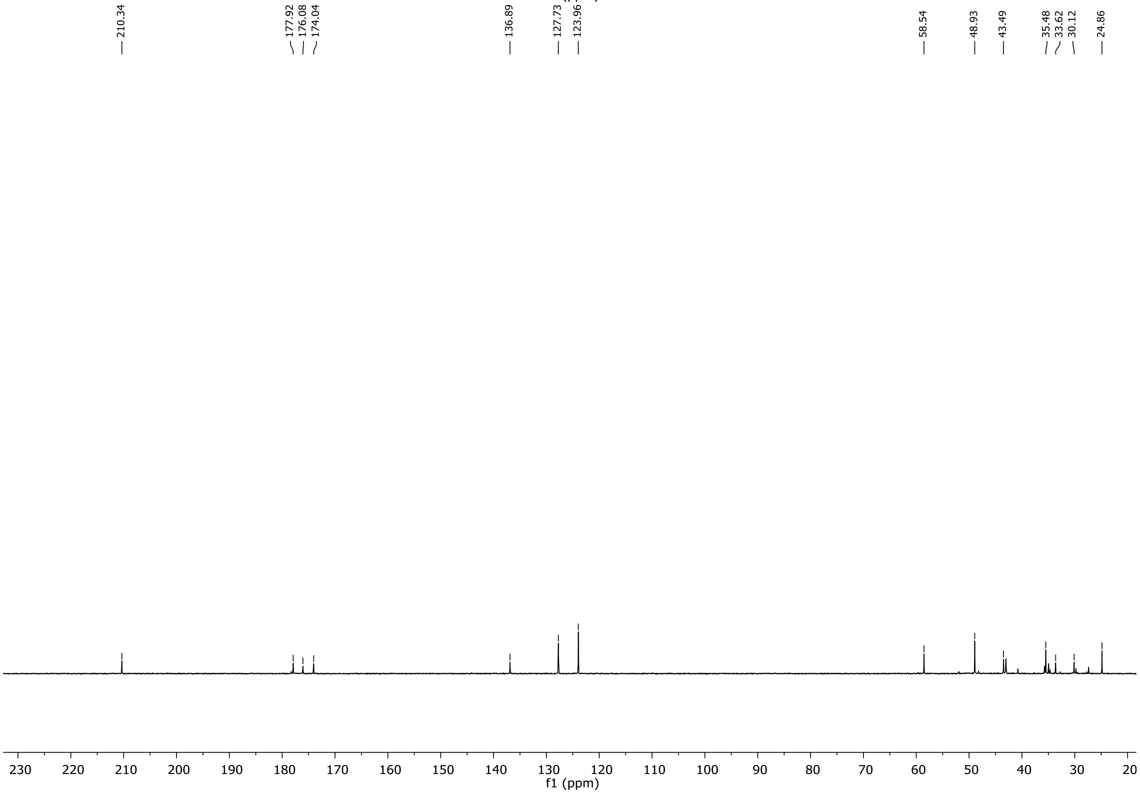

^{1}H NMR (300 MHz, $CDCl_3$) and ^{13}C NMR (75 MHz, $CDCl_3$) of compound **4a**

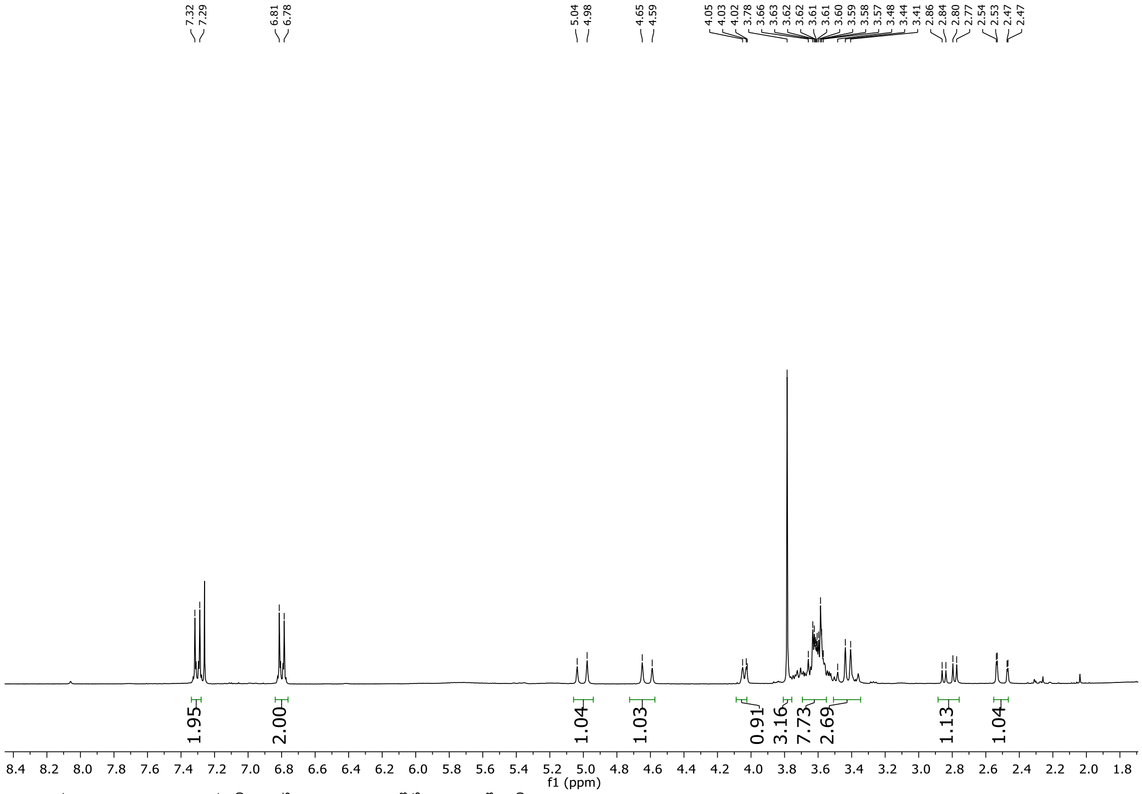

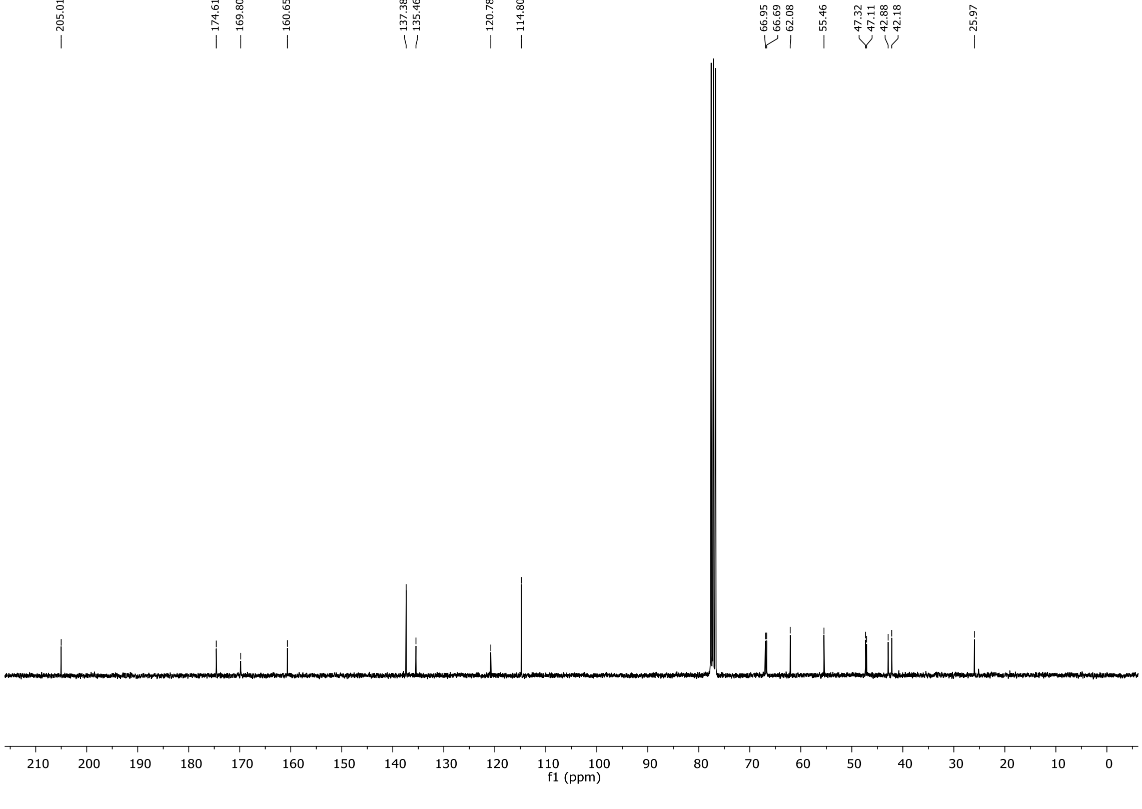

^{1}H NMR (300 MHz, $CDCl_3$) and ^{13}C NMR (75 MHz, $CDCl_3$) of compound **4b**

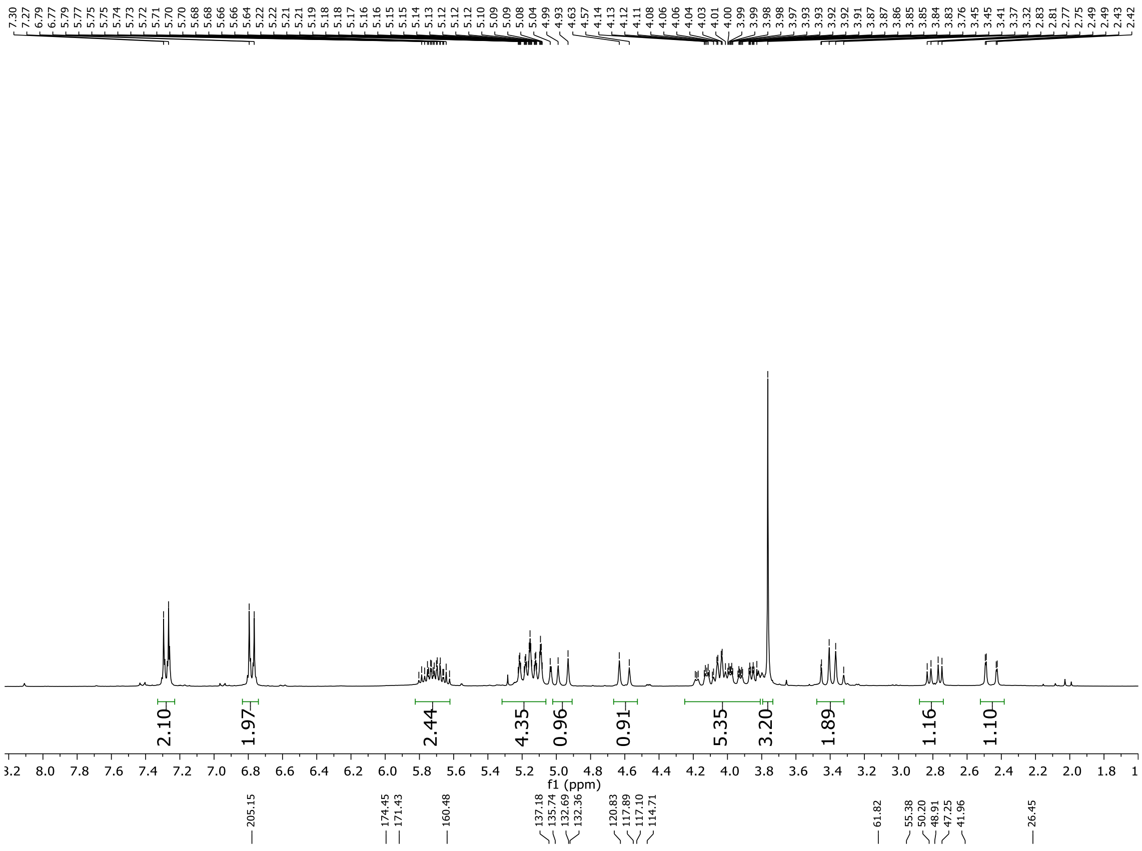

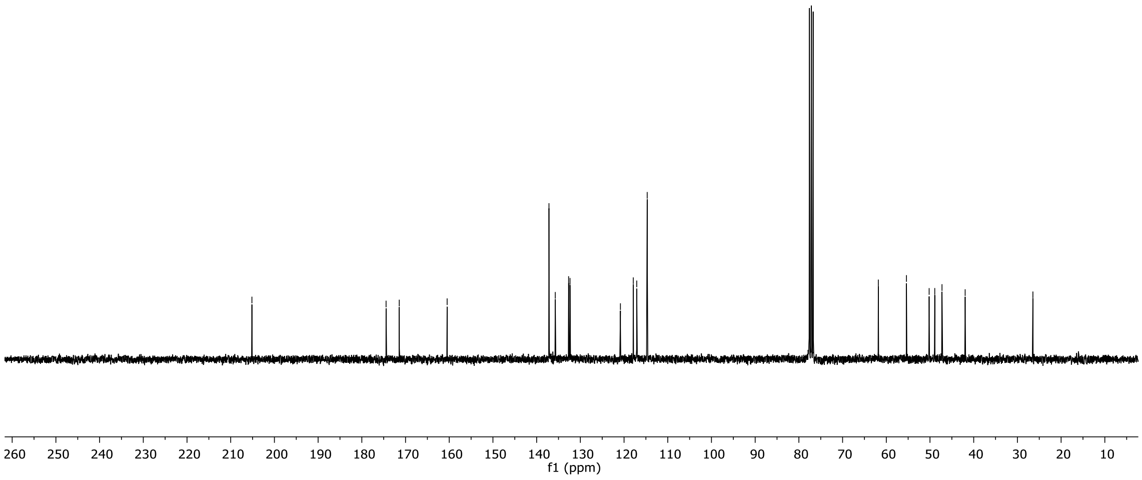

^{1}H NMR (300 MHz, $CDCl_3$) and ^{13}C NMR (75 MHz, $CDCl_3$) of compound **4c**

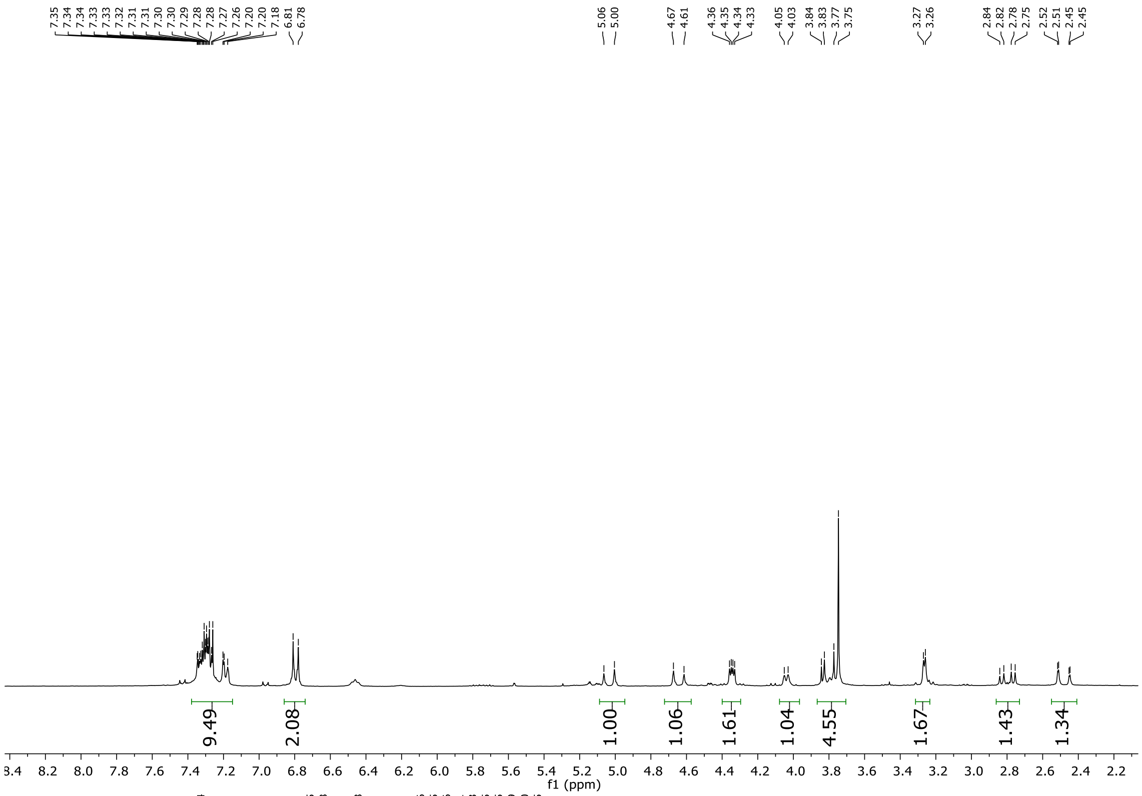

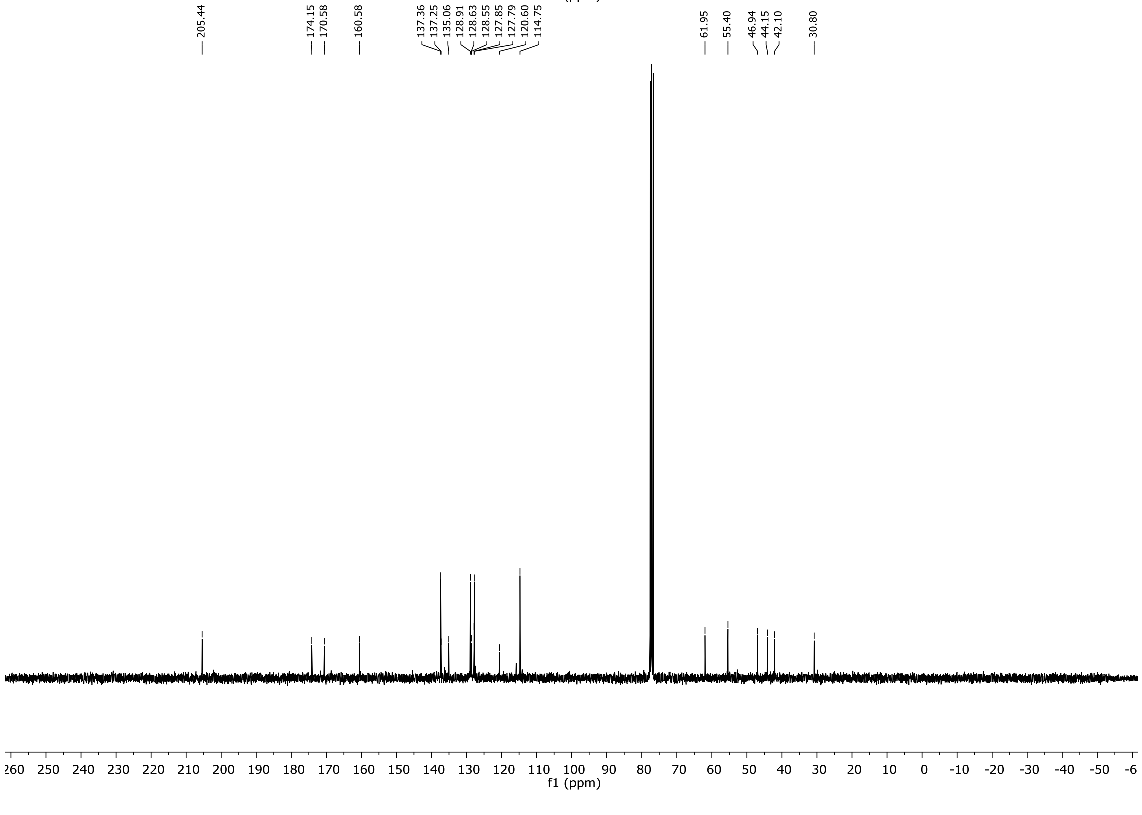

^1H NMR (300 MHz, $CDCl_3$) and ^{13}C NMR (75 MHz, $CDCl_3$) of compound **4d**

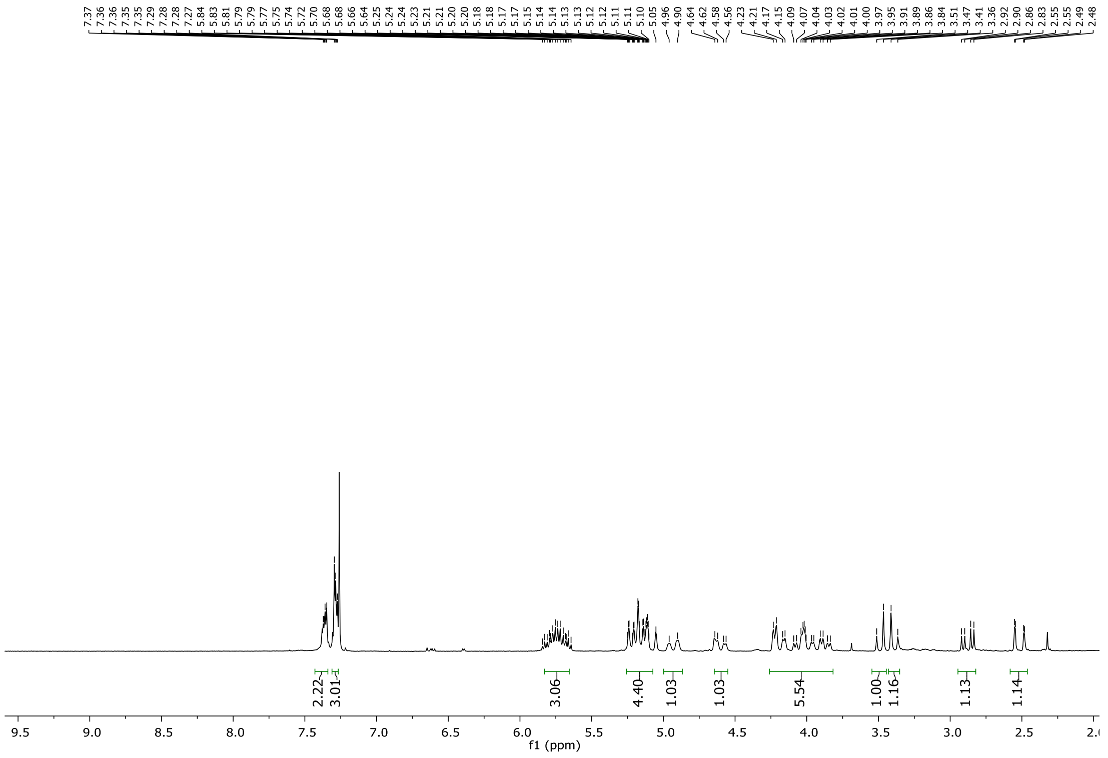

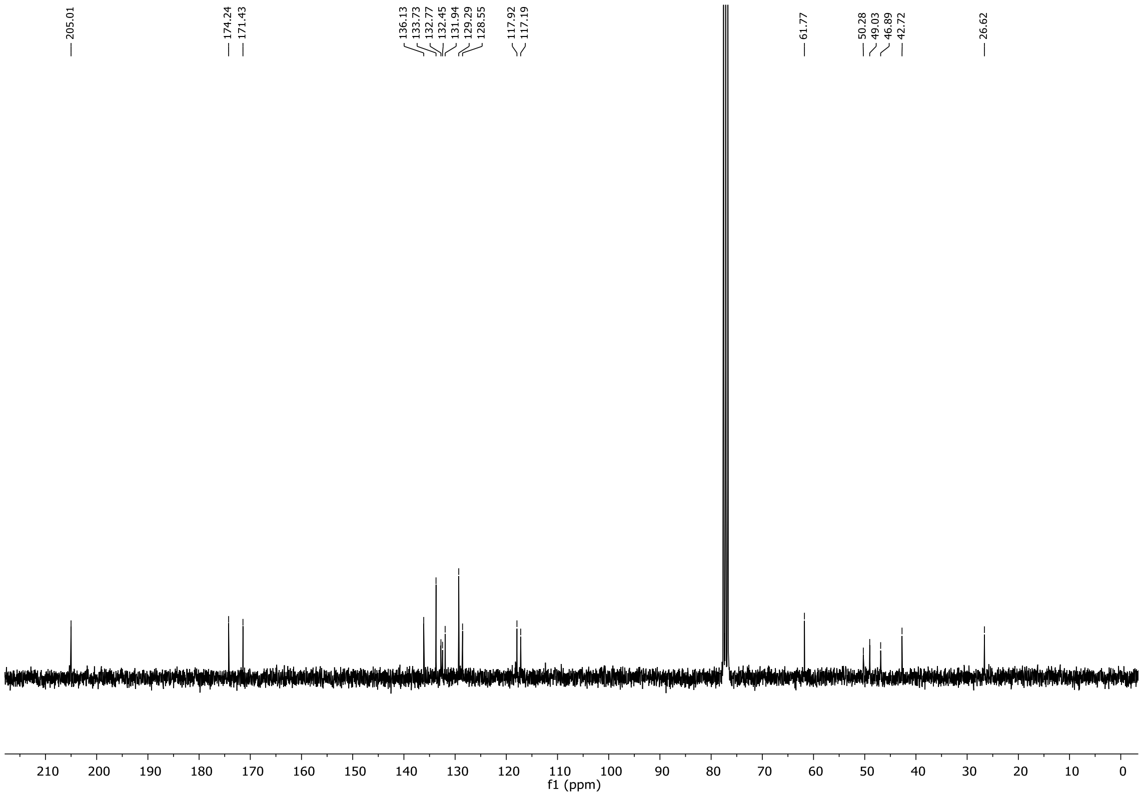

^{1}H NMR (300 MHz, $CDCl_3$) and ^{13}C NMR (75 MHz, $CDCl_3$) of compound **4e**

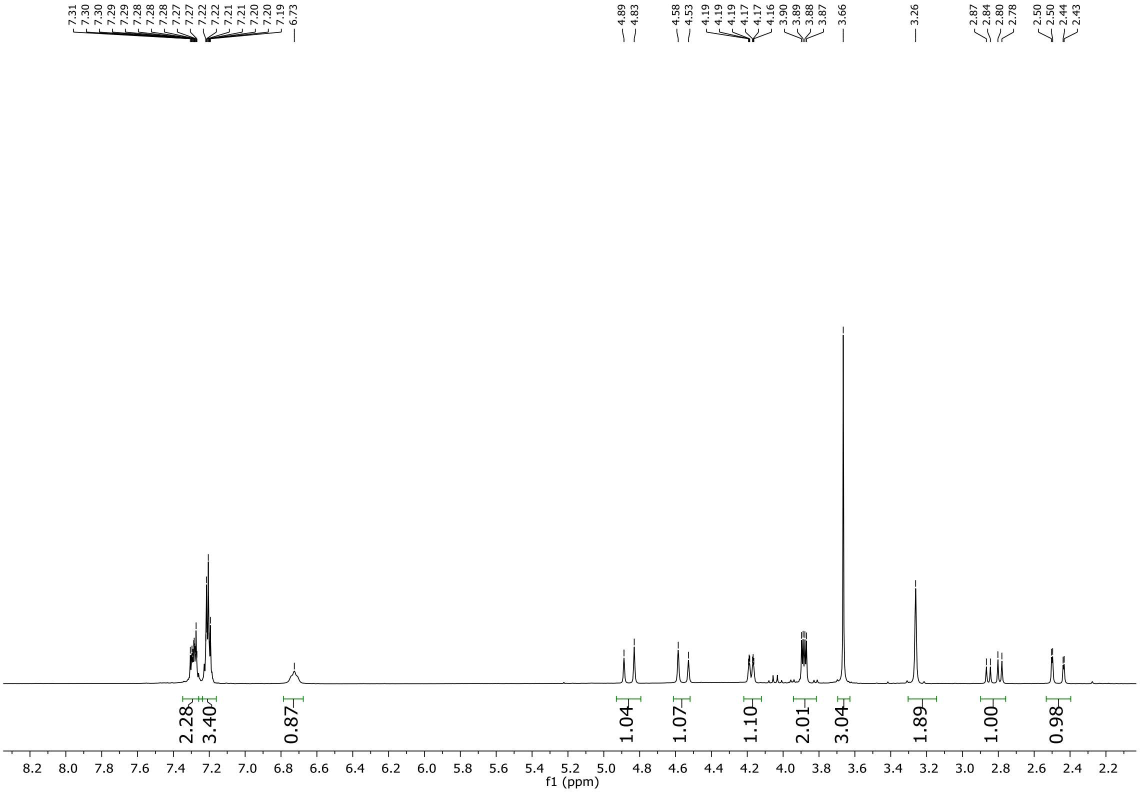

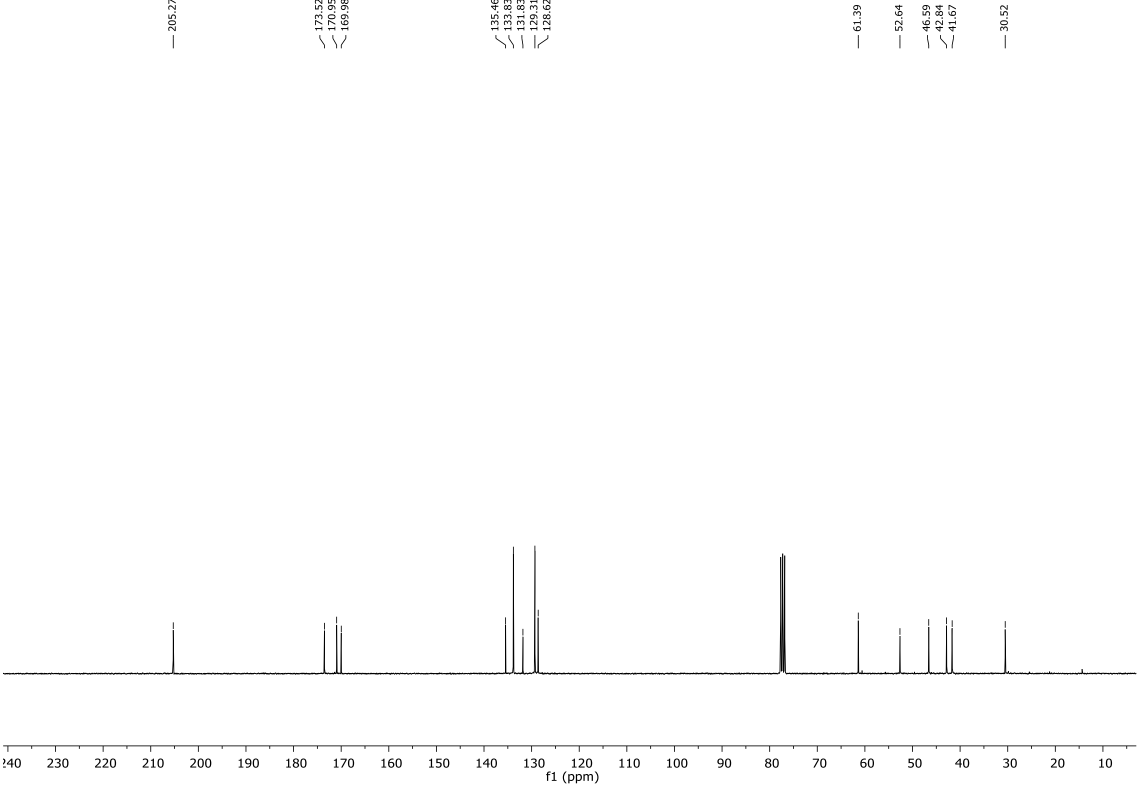

^{1}H NMR (300 MHz, $CDCl_3$) and ^{13}C NMR (75 MHz, $CDCl_3$) of compound **5a**

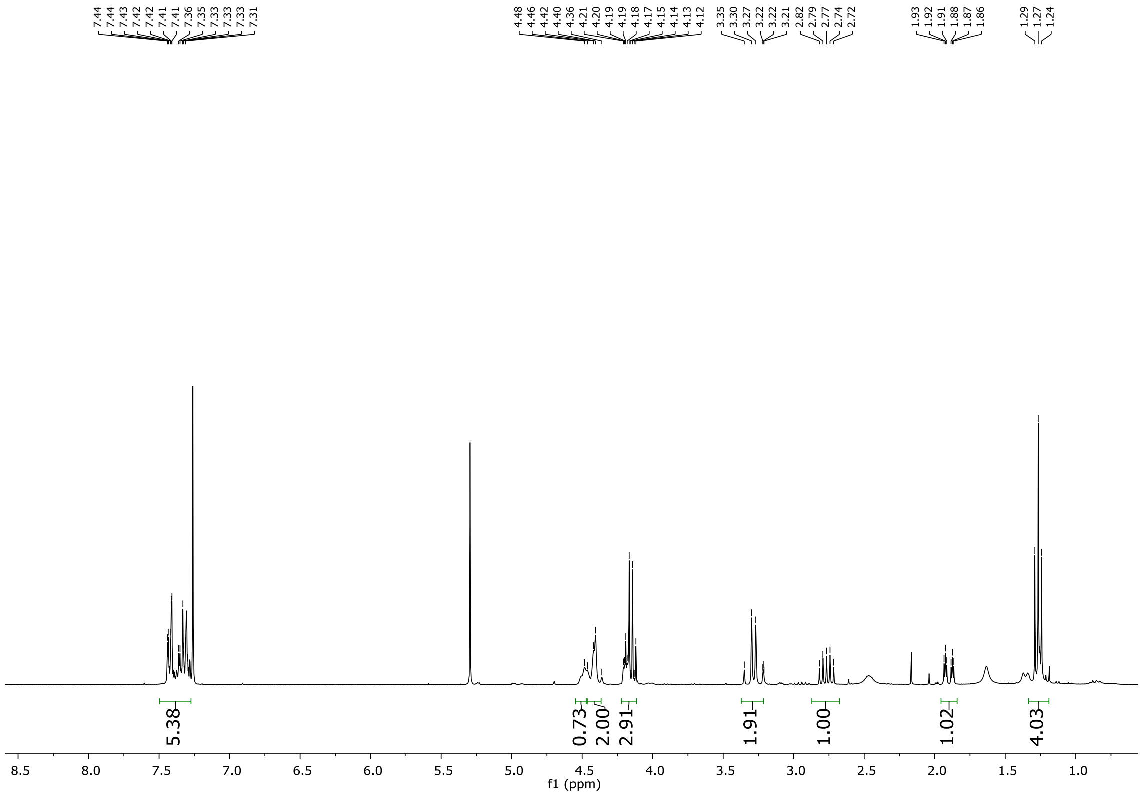

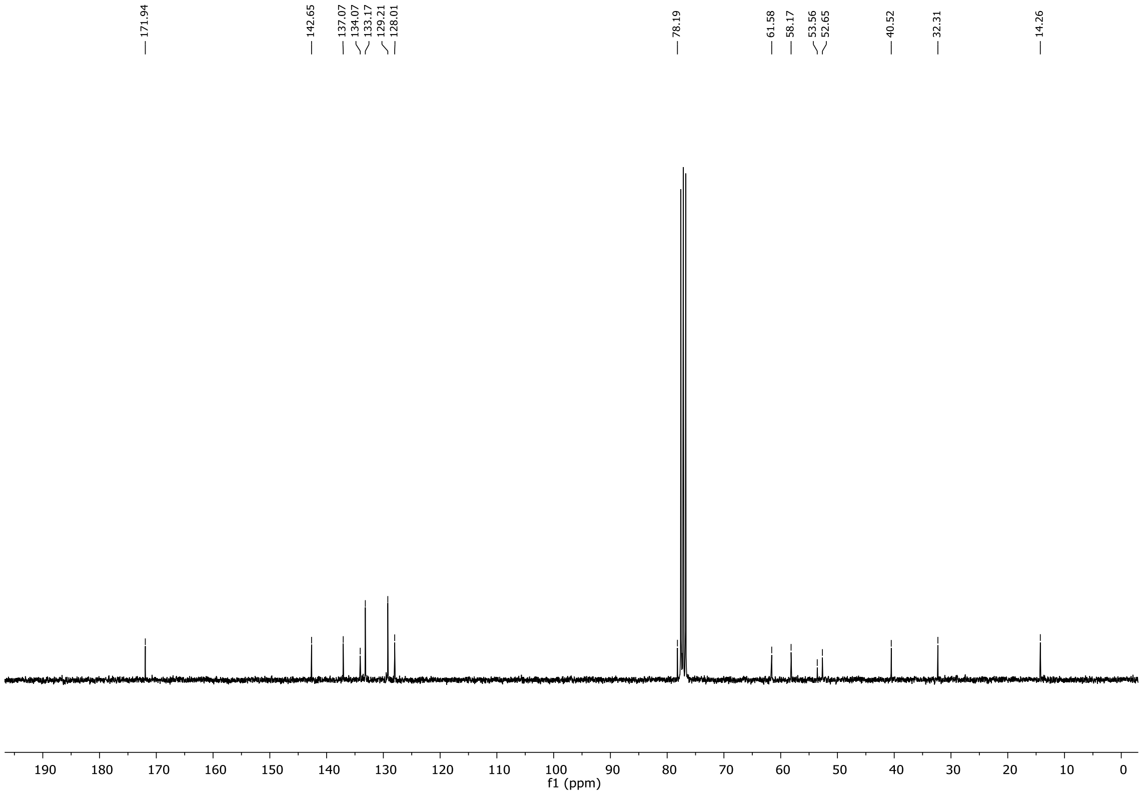

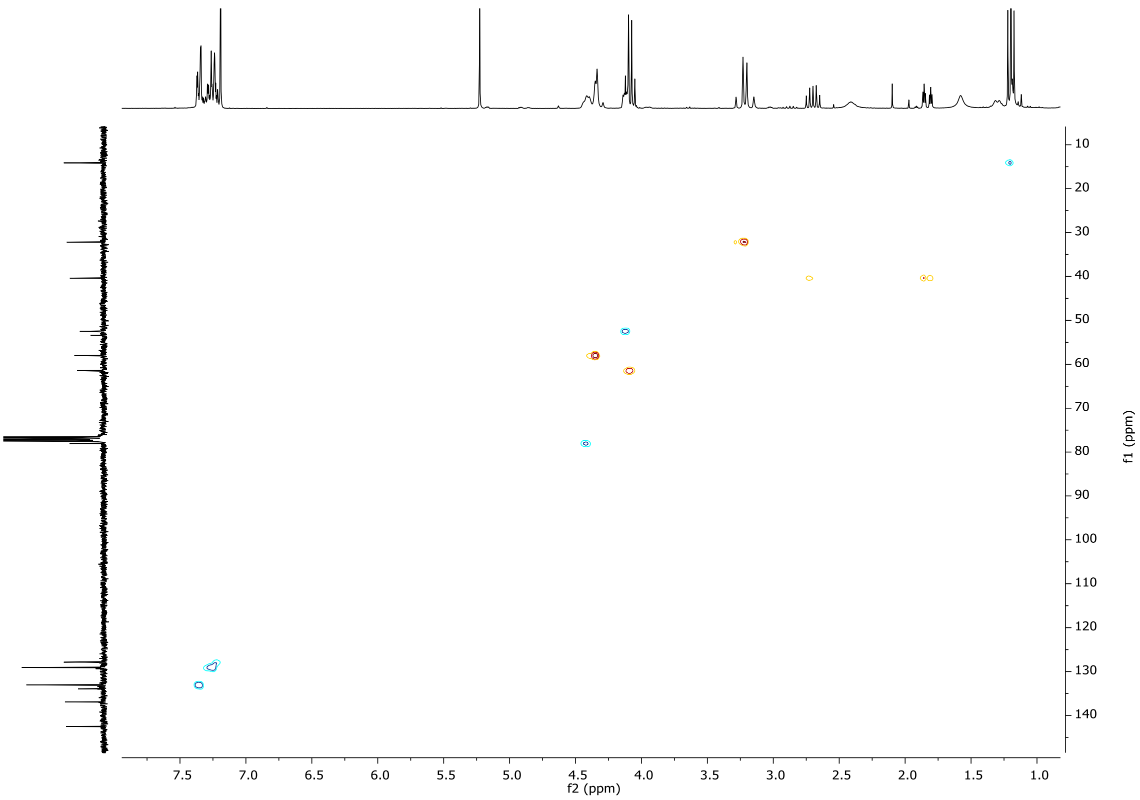

HSQC of compound **5a**

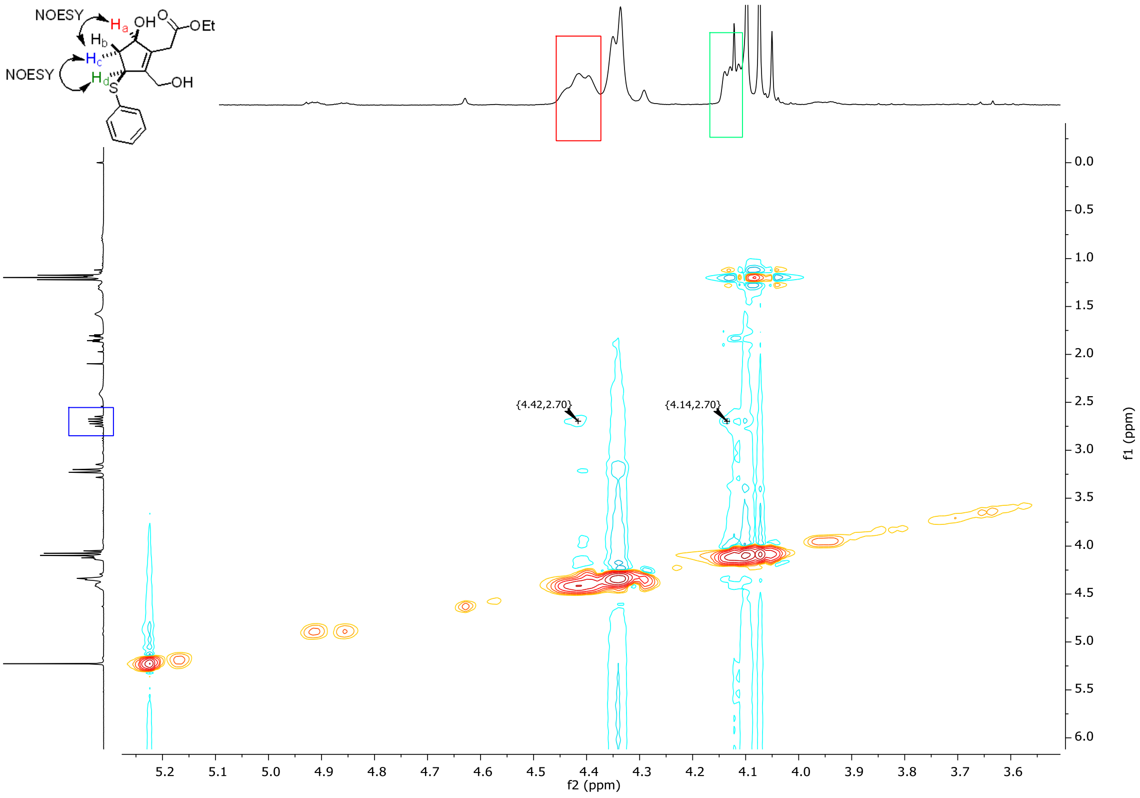

NOESY of compound **5a**

^{1}H NMR (300 MHz, $CDCl_3$) and ^{13}C NMR (75 MHz, $CDCl_3$) of compound **5b**

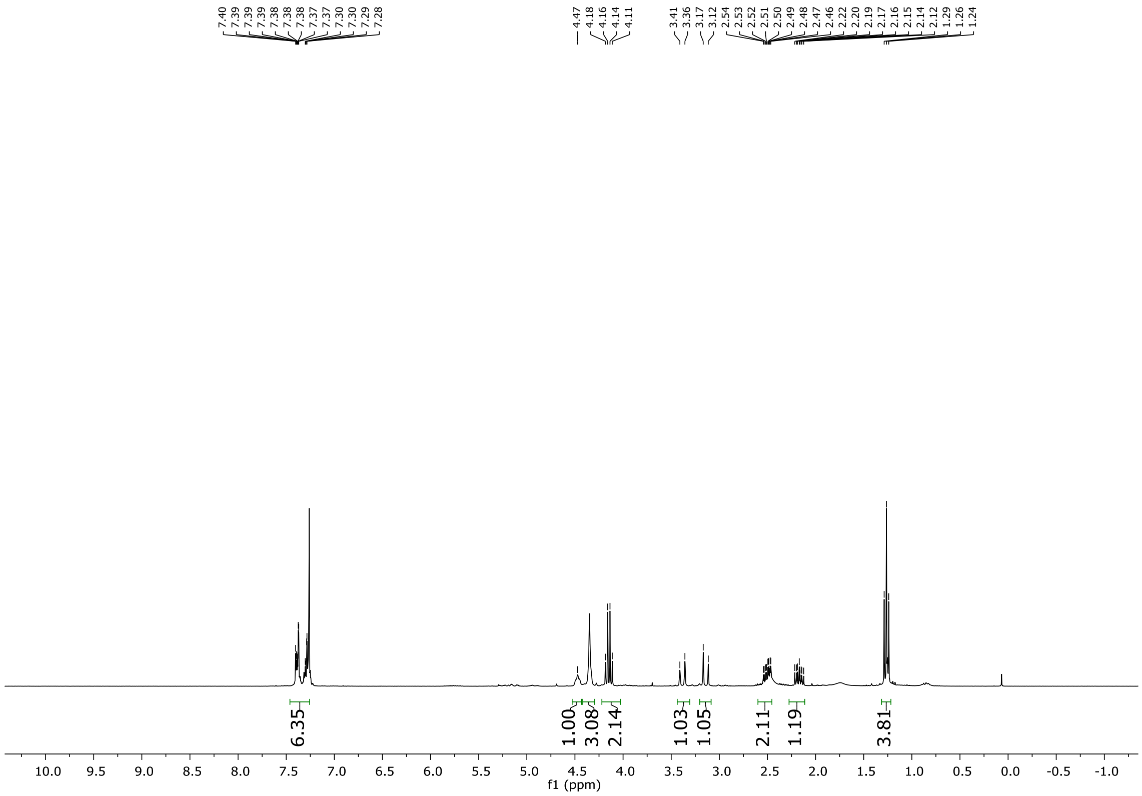

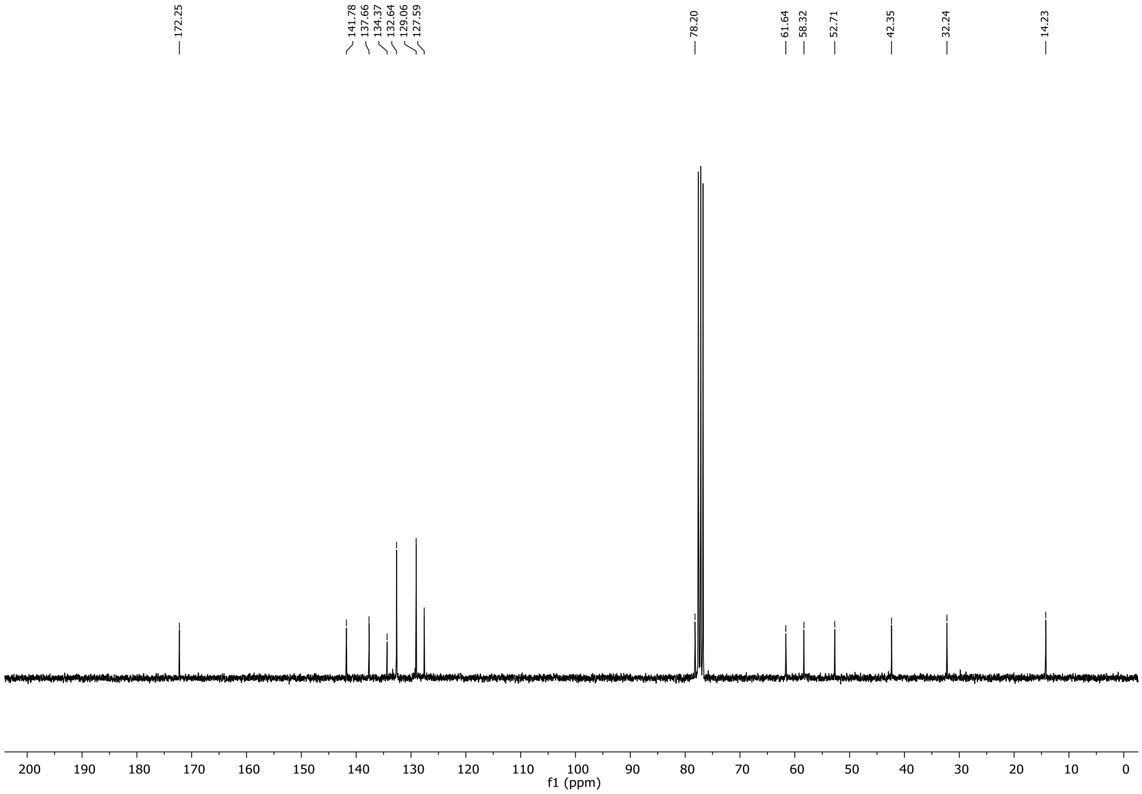

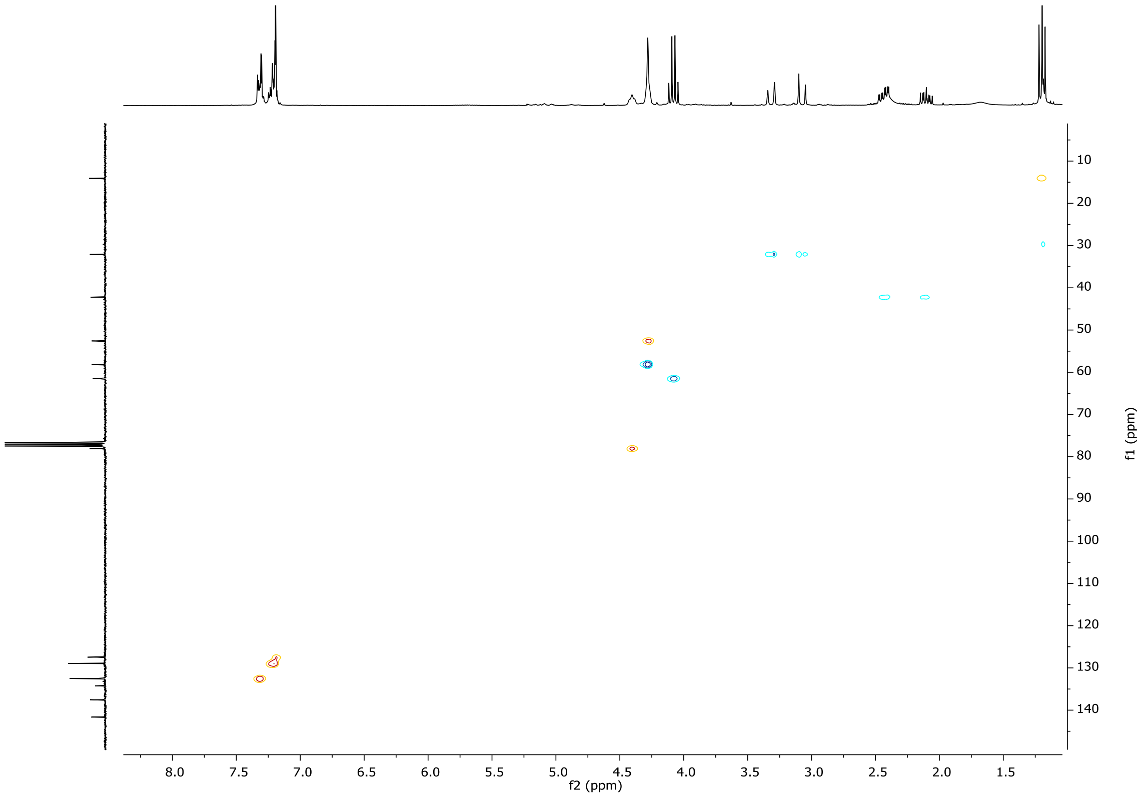

HSQC of compound **5b**

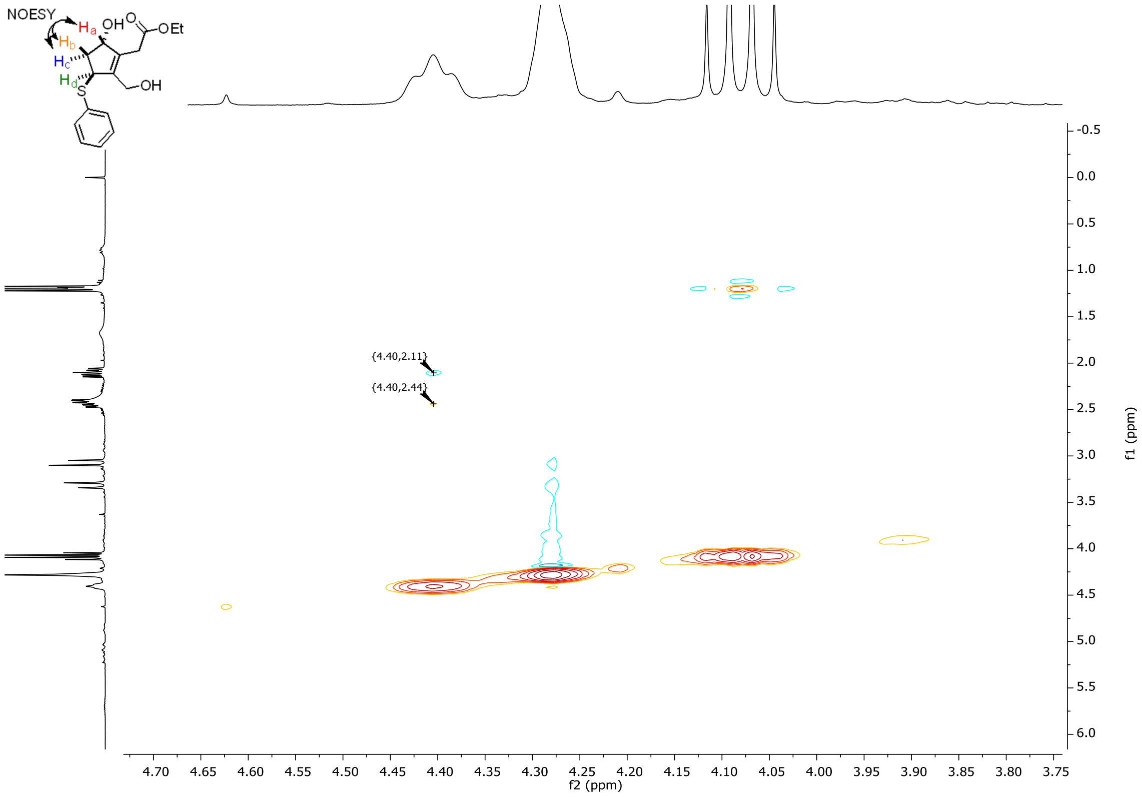

NOESY of compound **5b**

^{1}H NMR (300 MHz, $CDCl_3$) and ^{13}C NMR (75 MHz, $CDCl_3$) of compound **6**

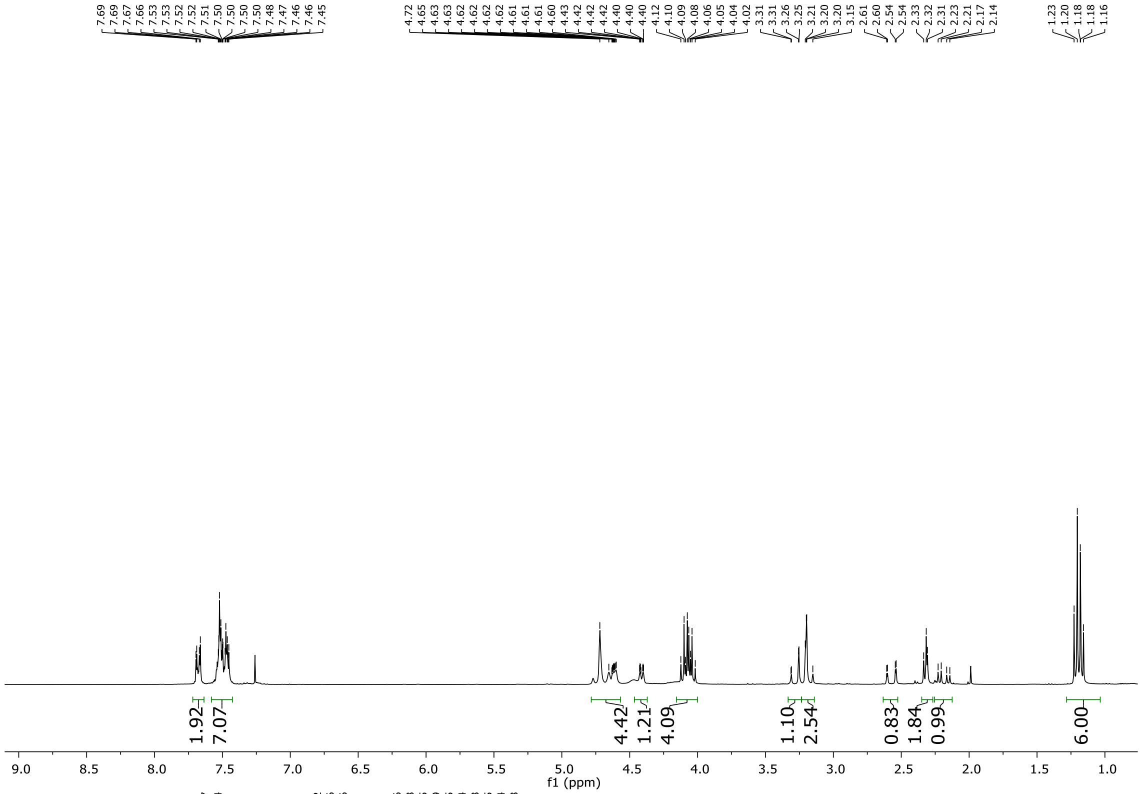

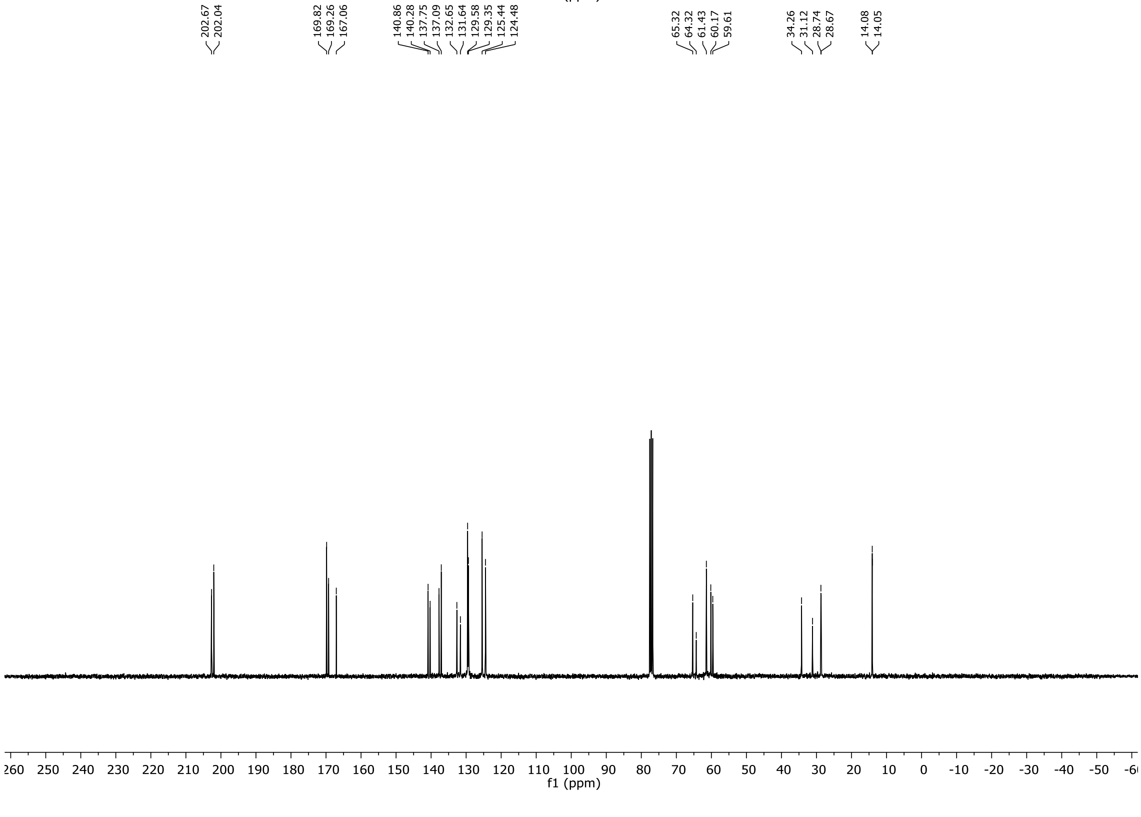

^{1}H NMR (300 MHz, $CDCl_3$) and ^{13}C NMR (75 MHz, $CDCl_3$) of compound **7**

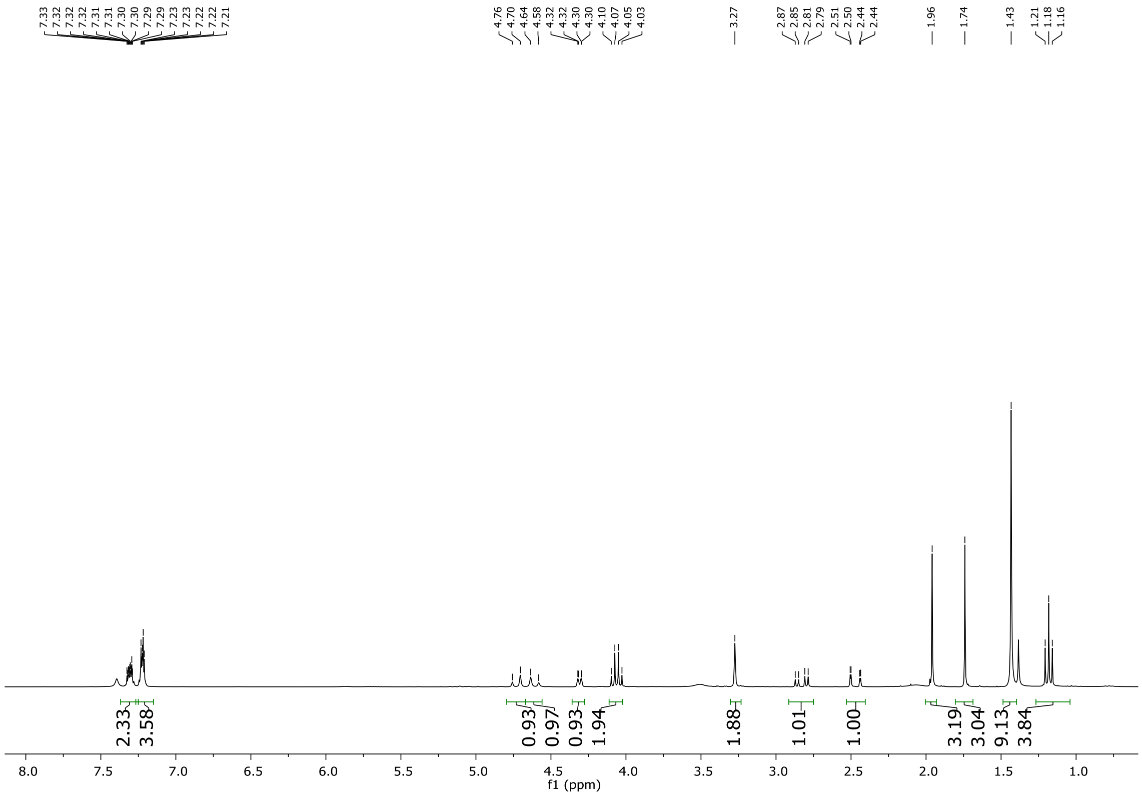

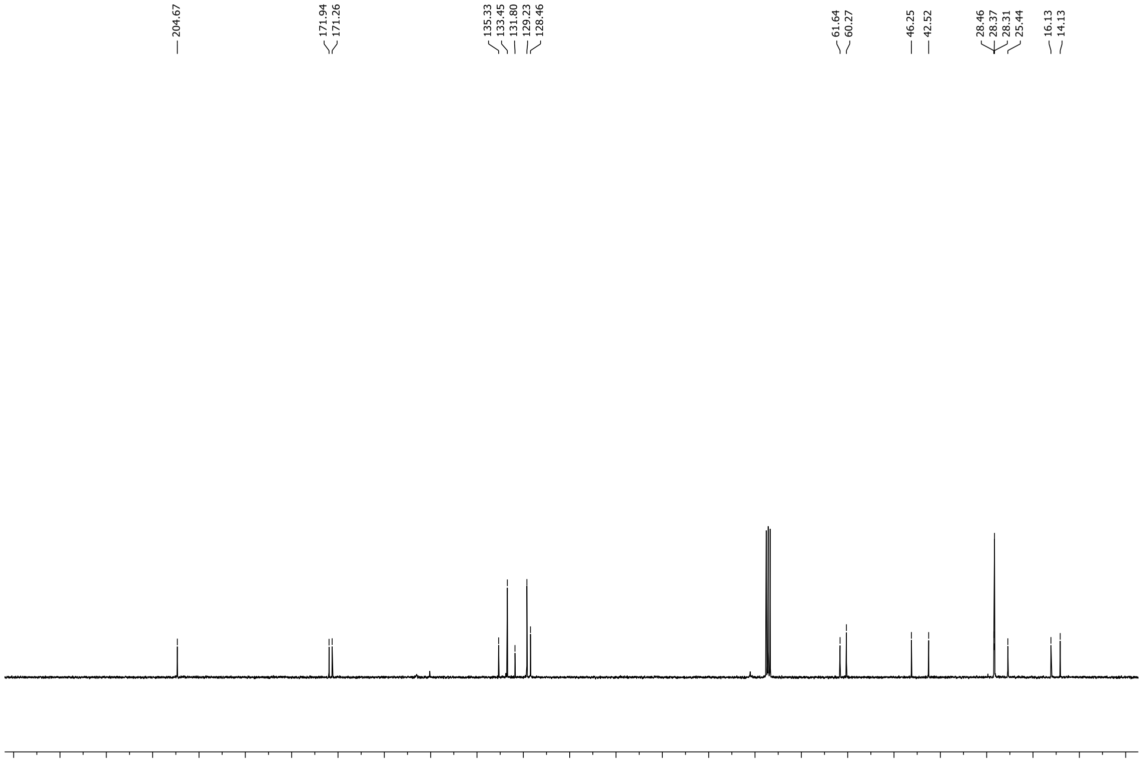

^{1}H NMR (300 MHz, $CDCl_3$) and ^{13}C NMR (75 MHz, $CDCl_3$) of compound **3**

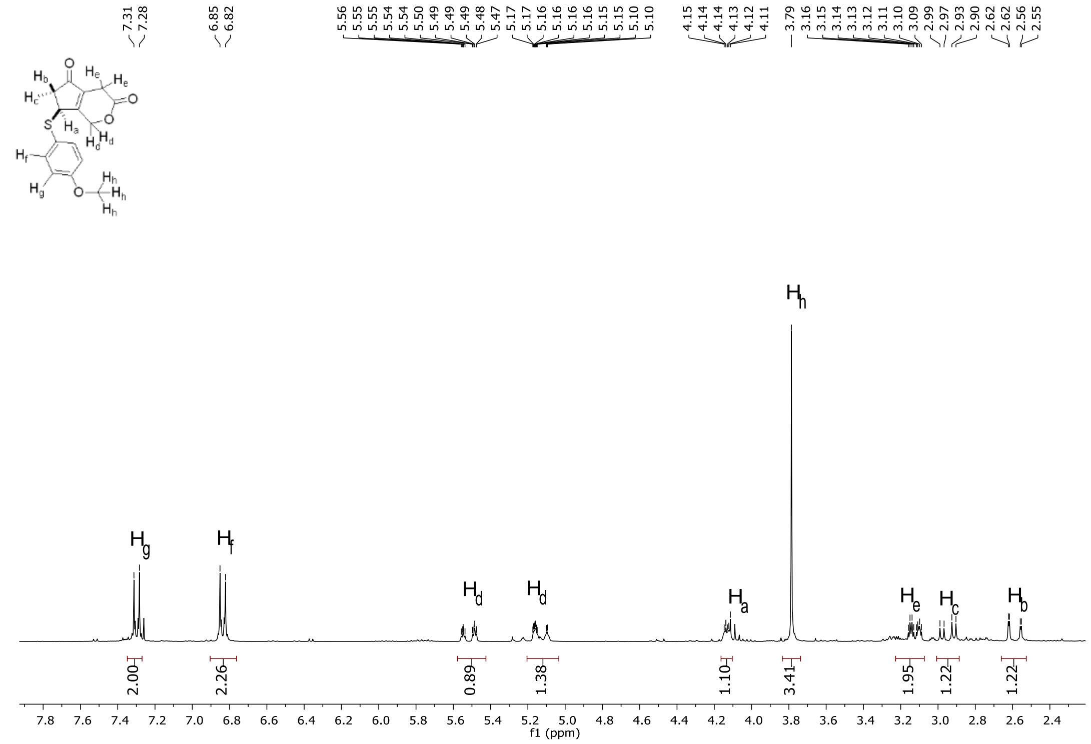

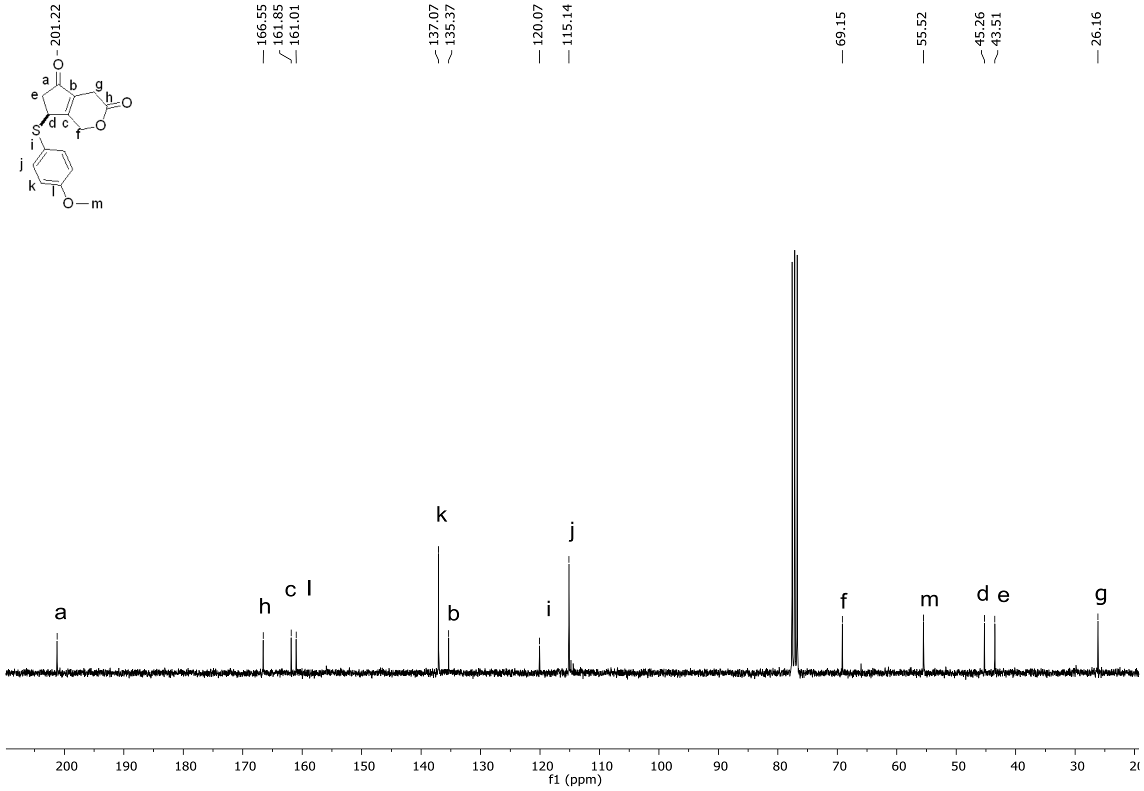

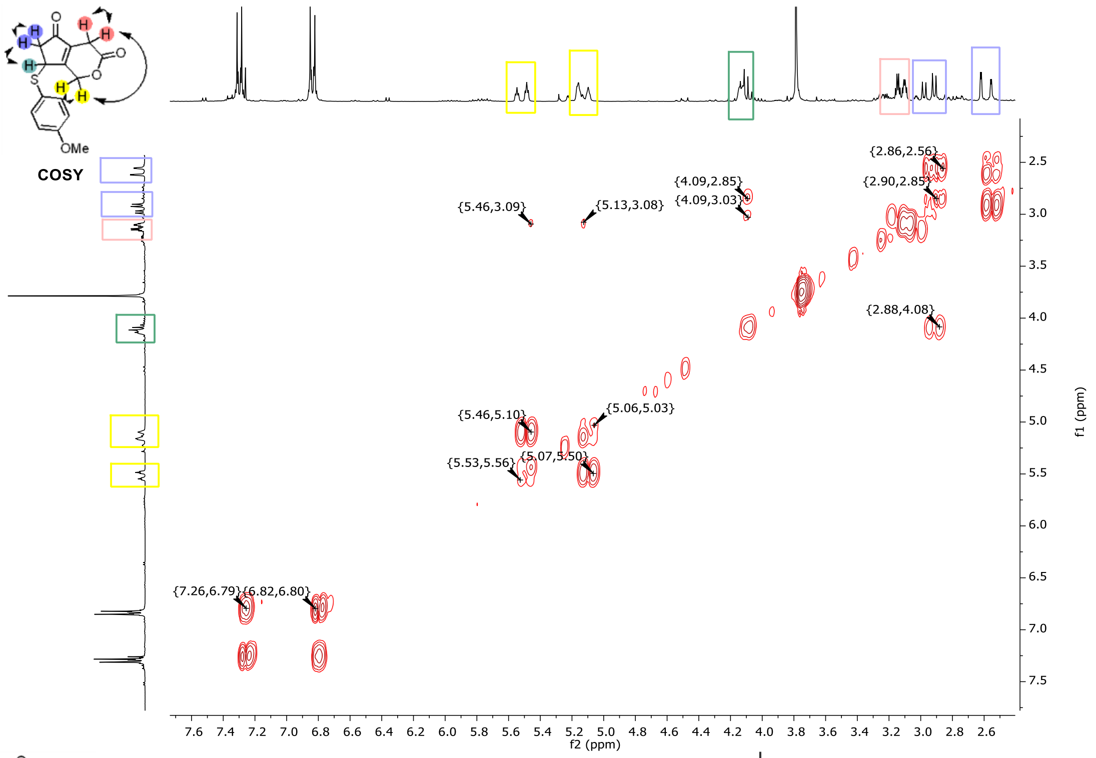

COSY
OMe
{2.86,2.56}
{2.90,2.85}
{4.09,2.85}
{4.09,3.03}
{5.46,3.09}
{5.13,3.08}
{2.88,4.08}
{5.46,5.10}
{5.06,5.03}
{5.53,5.56}
{5.07,5.50}
{7.26,6.79}
{6.82,6.80}
f2 (ppm)
f1 (ppm)

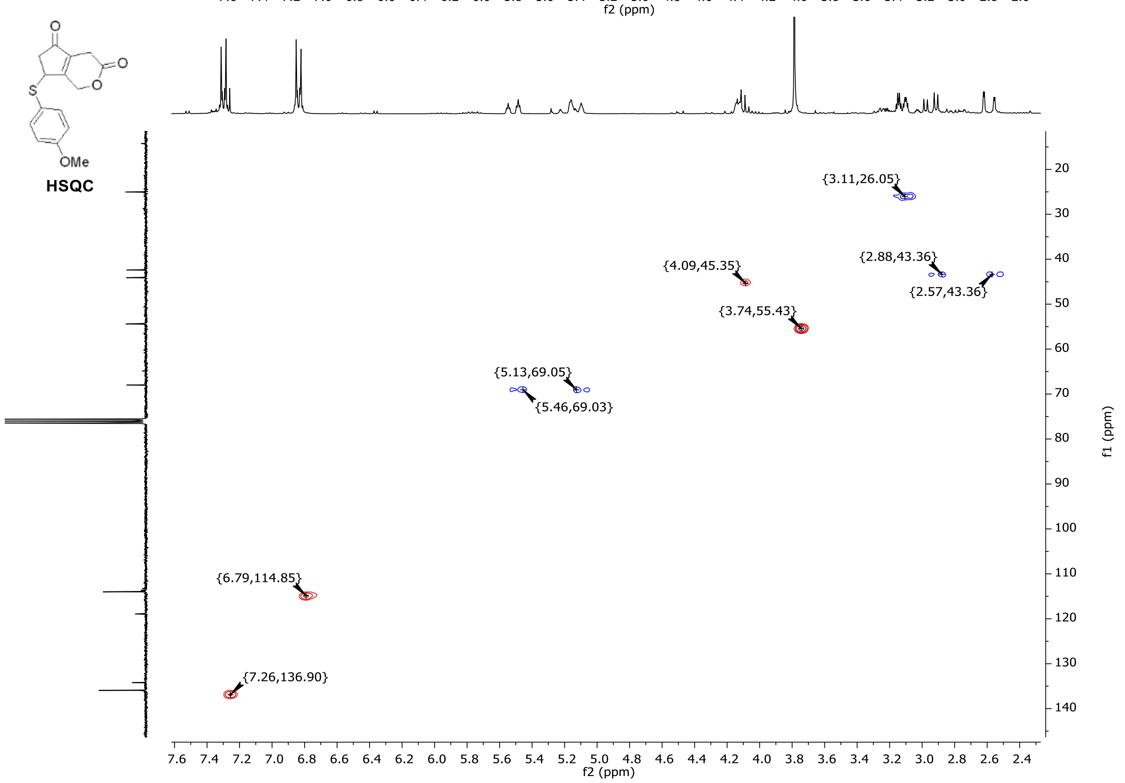

HSQC
OMe
{3.11,26.05}
{2.88,43.36}
{4.09,45.35}
{2.57,43.36}
{3.74,55.43}
{5.13,69.05}
{5.46,69.03}
{6.79,114.85}
{7.26,136.90}
f2 (ppm)
f1 (ppm)

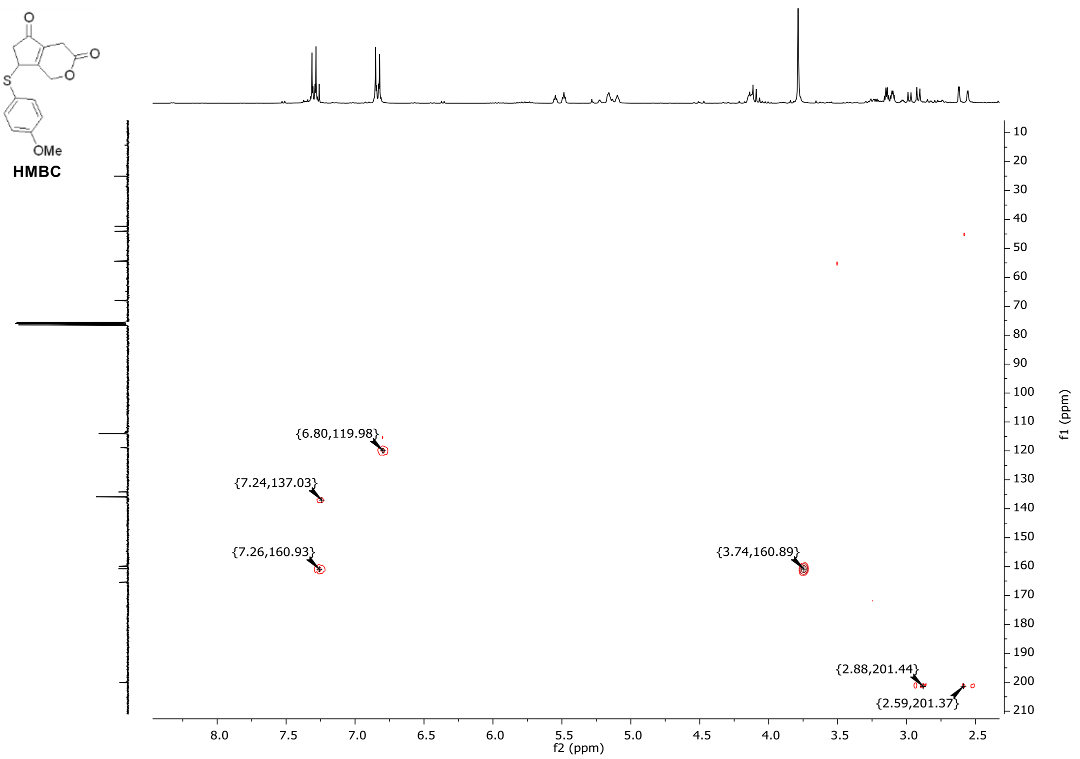
OMe
HMBC
{6.80,119.98}
{7.24,137.03}
{7.26,160.93}
{3.74,160.89}
{2.88,201.44}
{2.59,201.37}
f2 (ppm)
f1 (ppm)

HRMS copies:

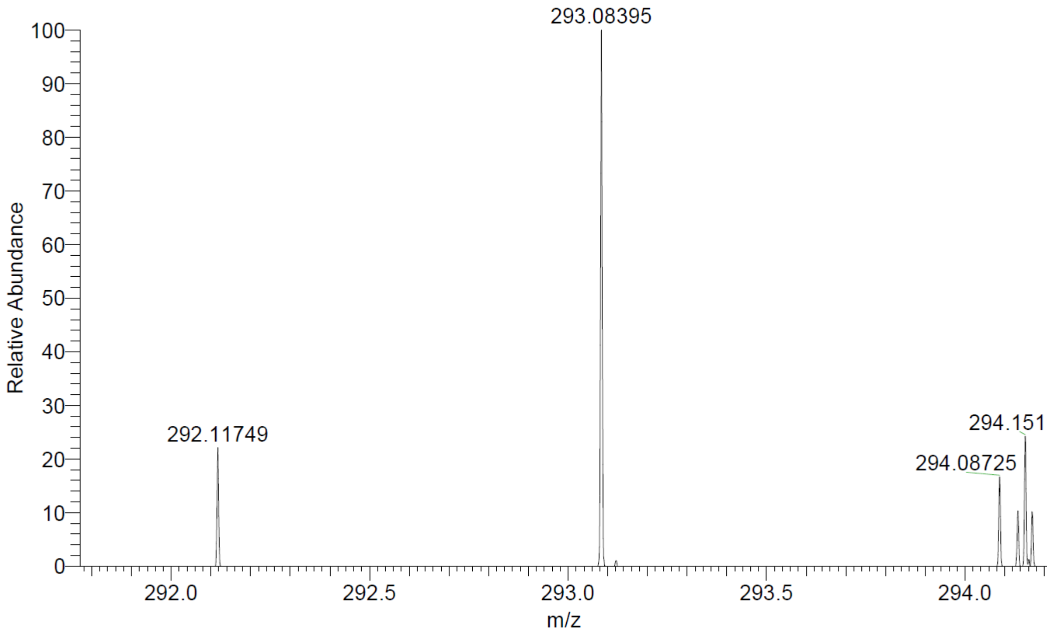

ESI-MS of compound **3a**

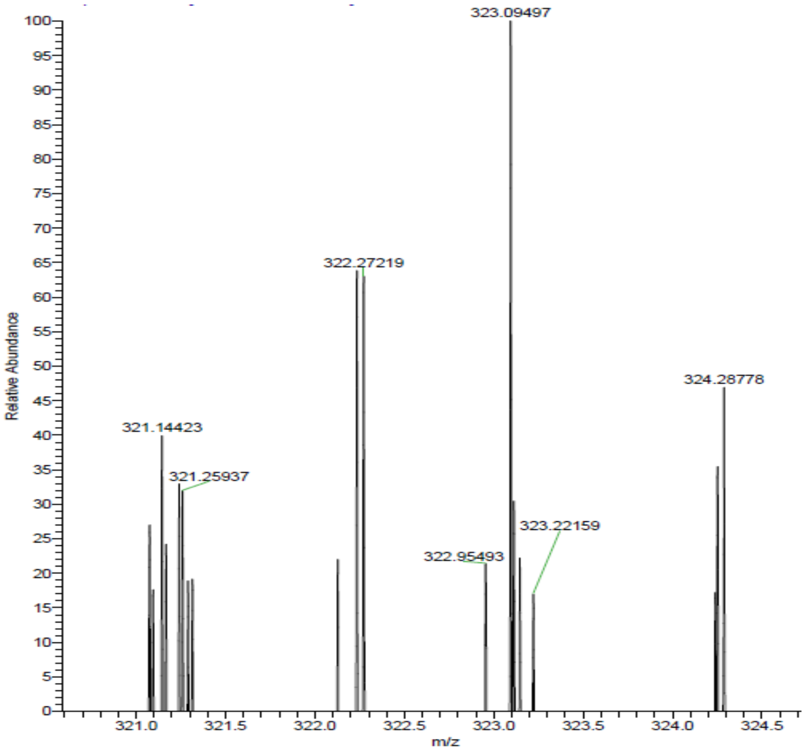

ESI-MS of compound **3b**

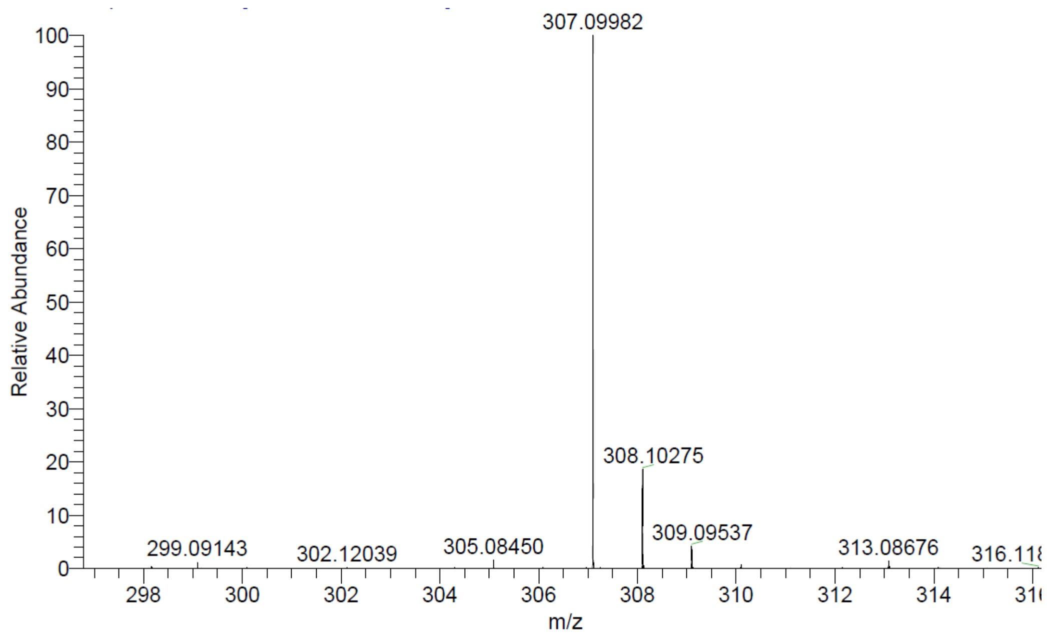

ESI-MS of compound **3c**

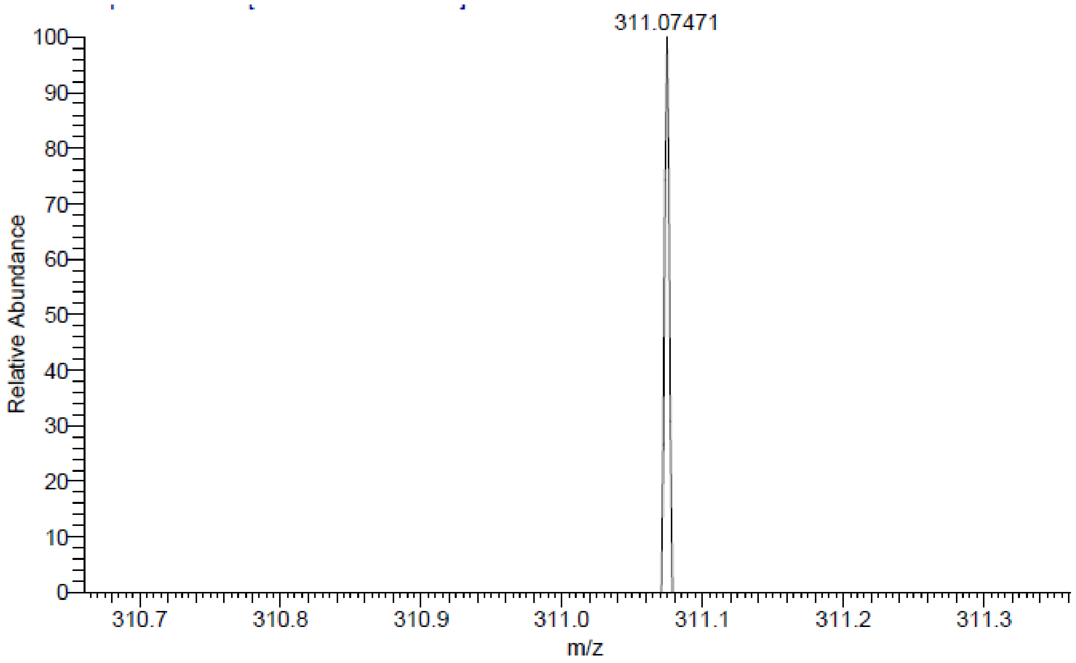

ESI-MS of compound **3d**

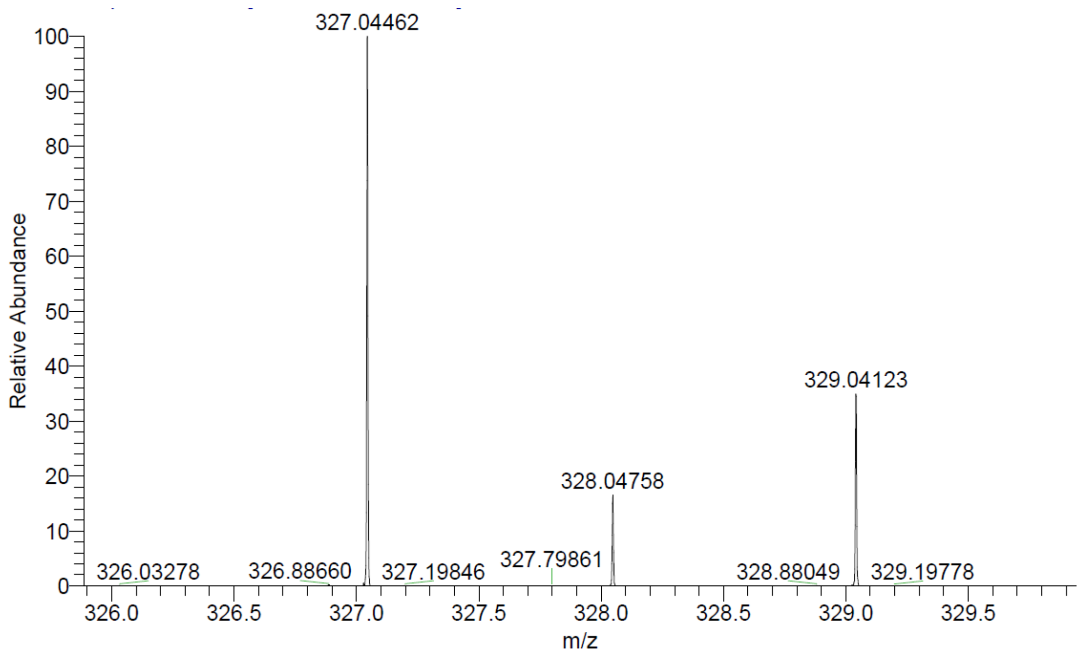

ESI-MS of compound **3e**

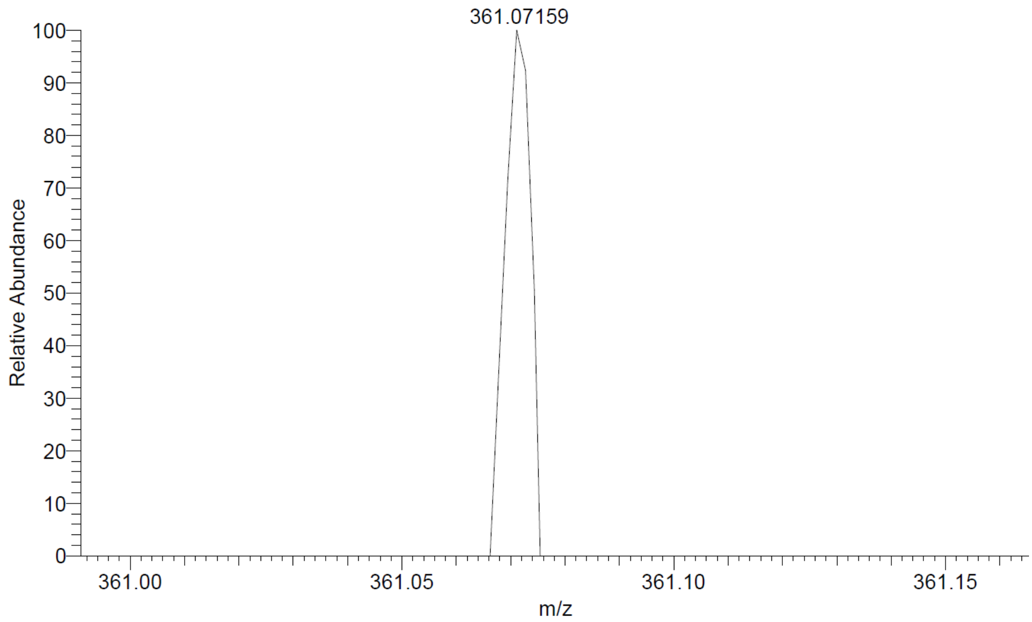

ESI-MS of compound **3f**

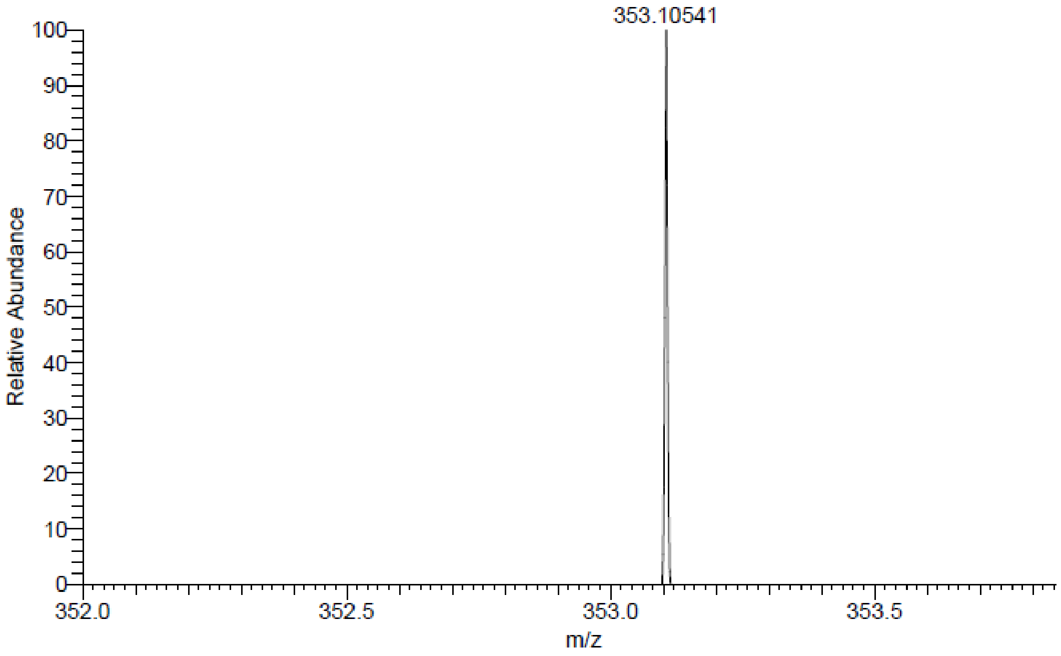

ESI-MS of compound **3g**

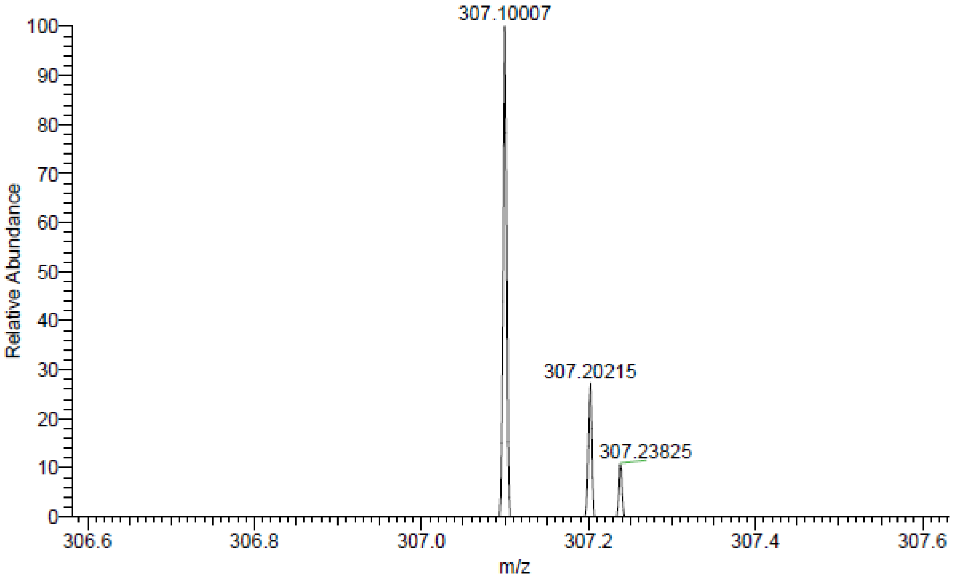

ESI-MS of compound **3h**

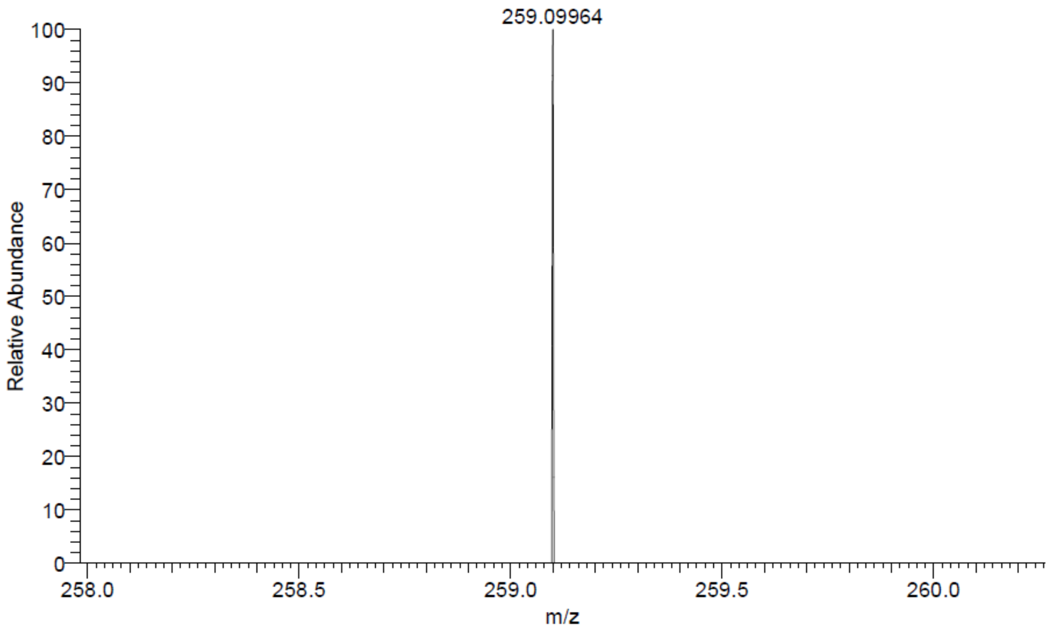

ESI-MS of compound **3i**

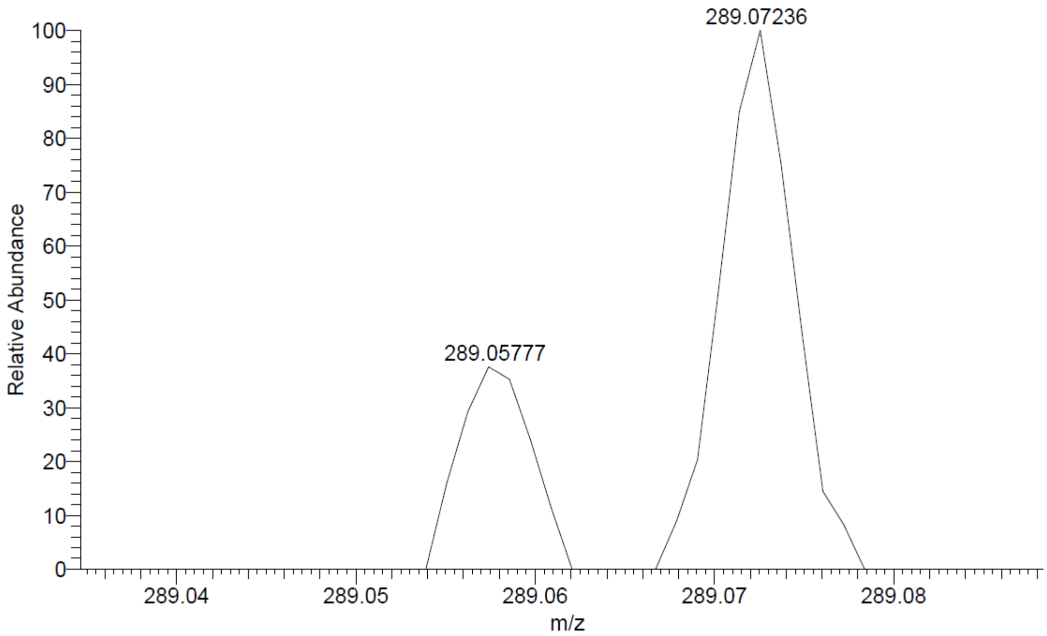

ESI-MS of compound **3j**

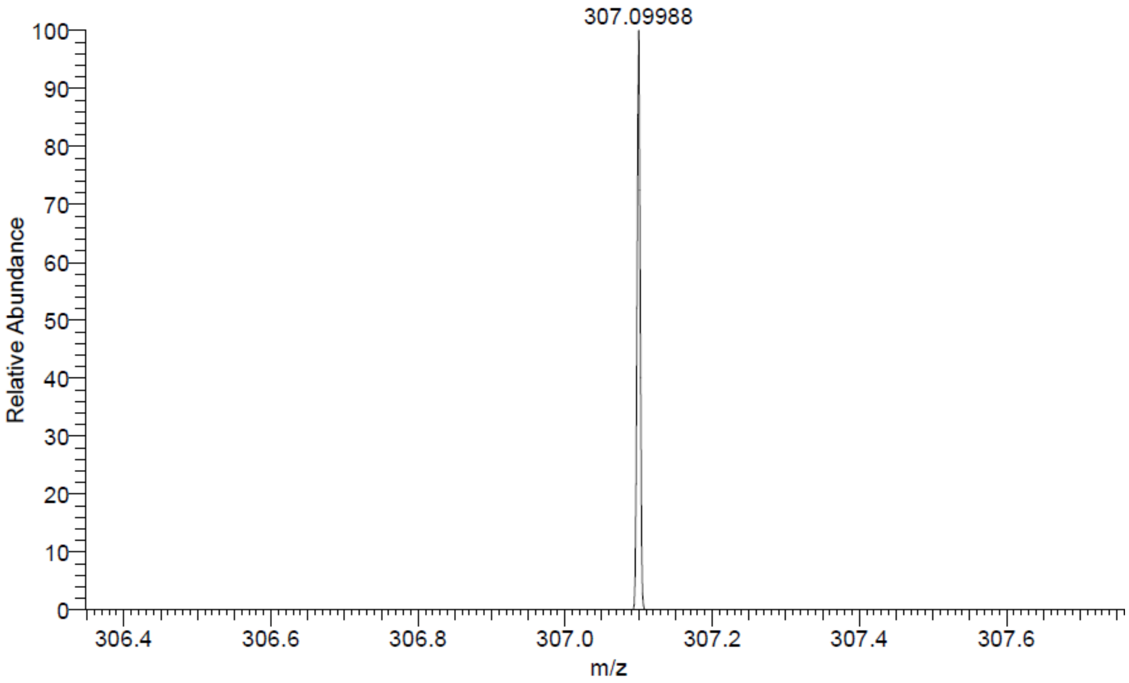

ESI-MS of compound **3k**

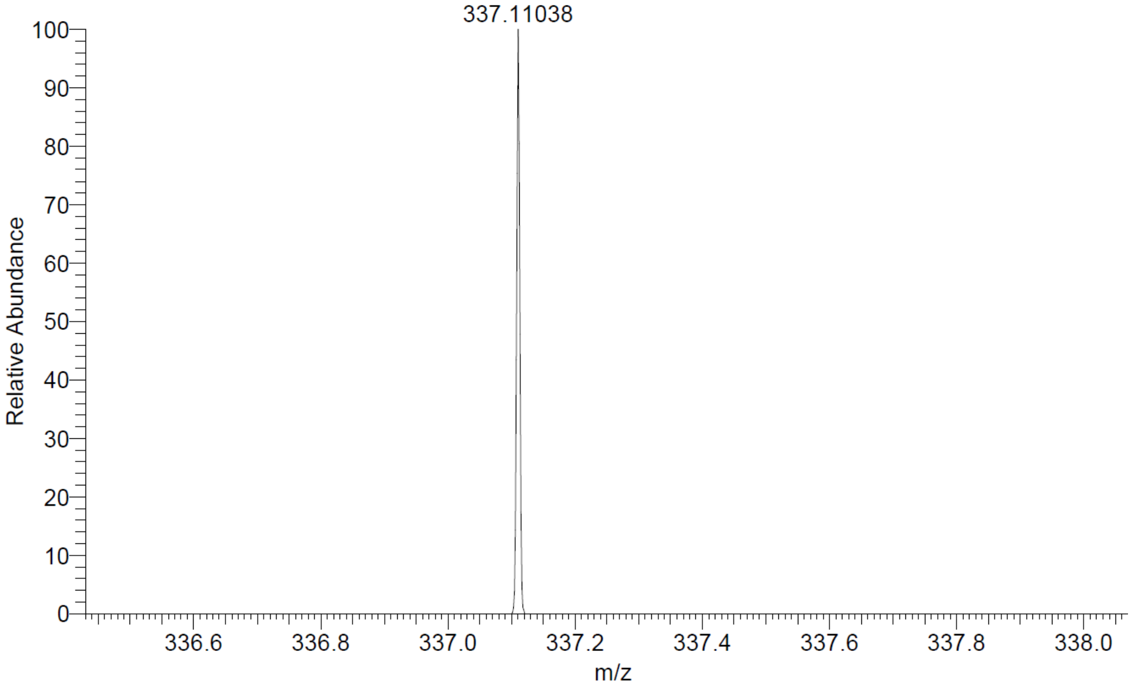

ESI-MS of compound **3l**

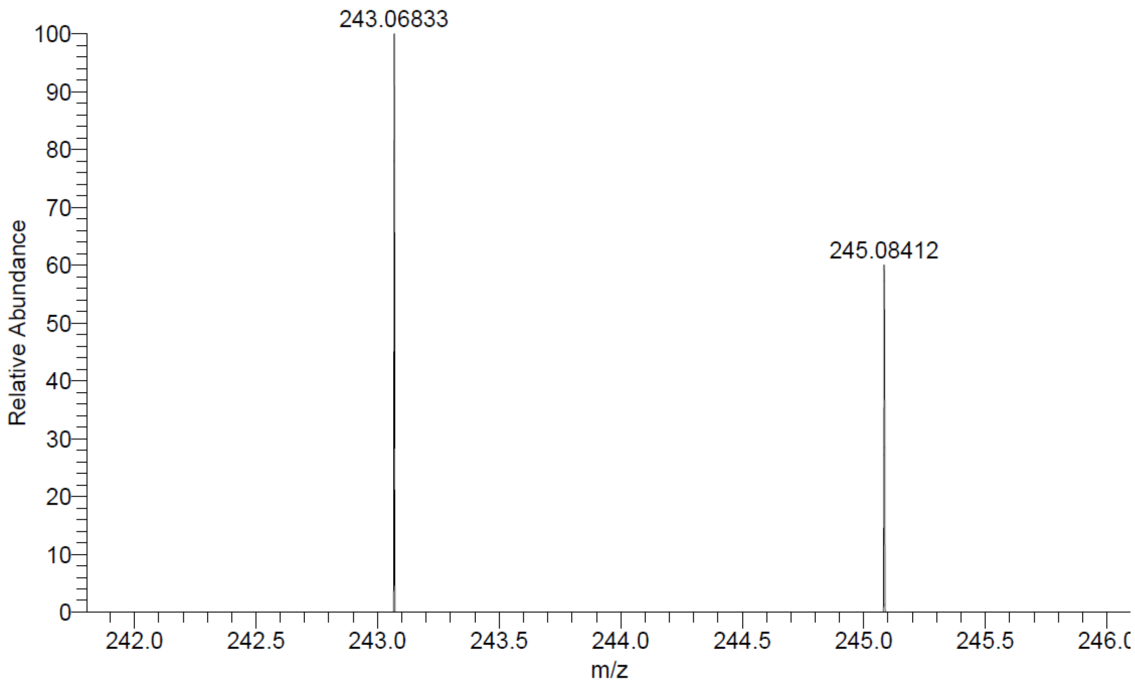

ESI-MS of compound **3m**

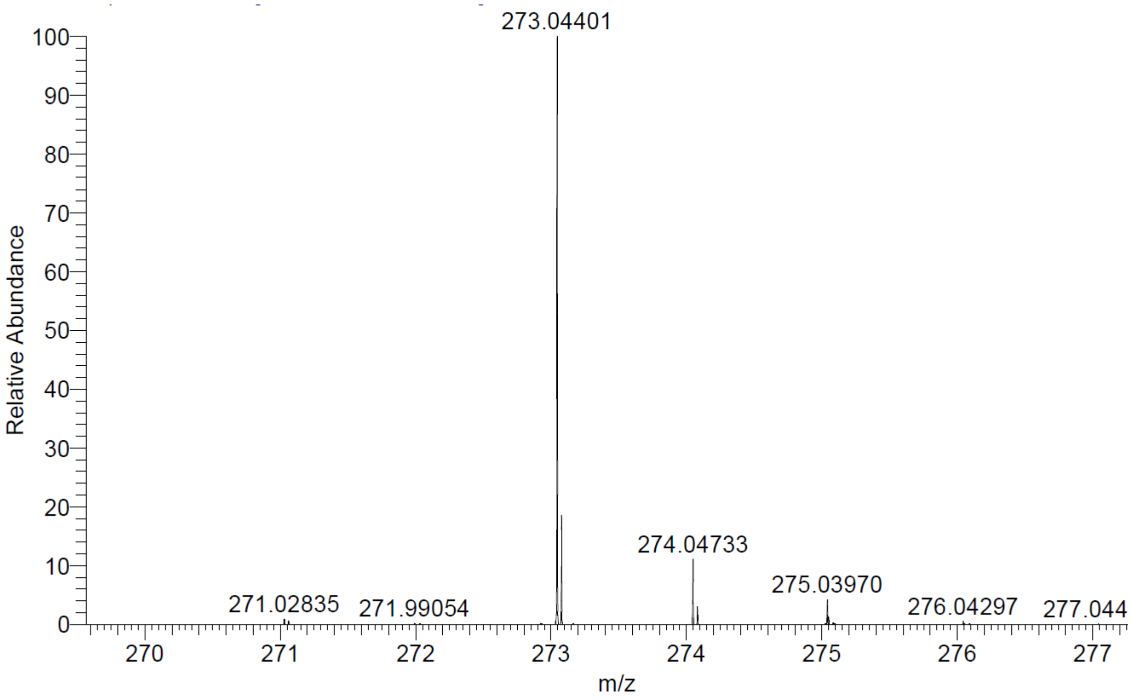

ESI-MS of compound **3n**

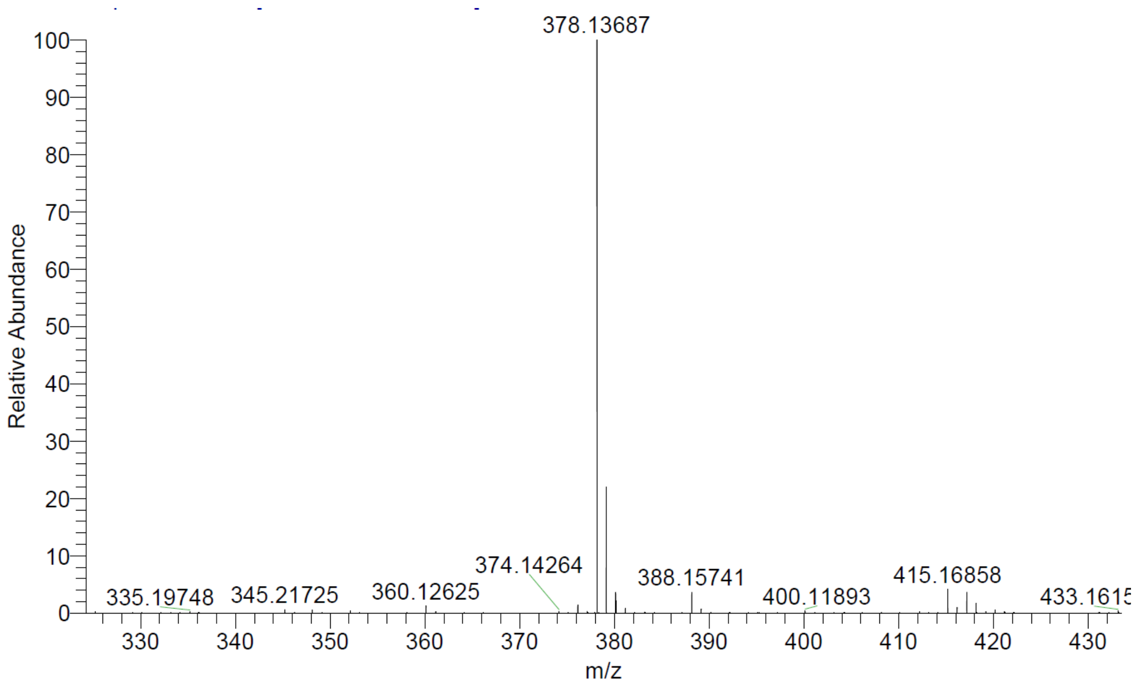

ESI-MS of compound **4a**

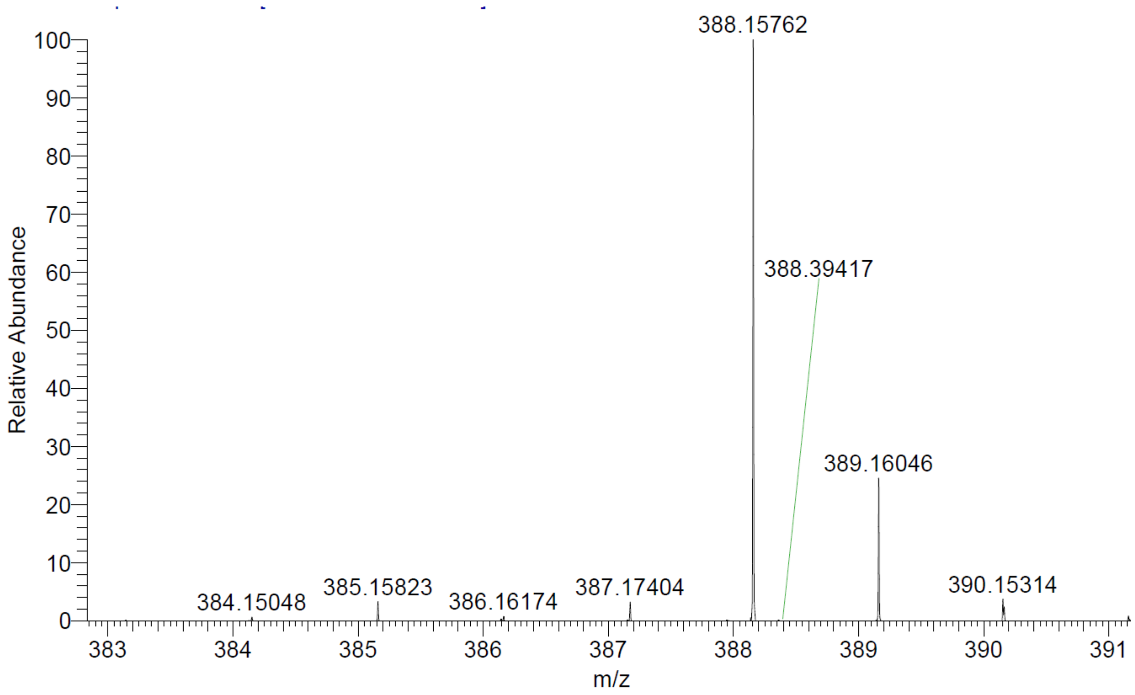

ESI-MS of compound **4b**

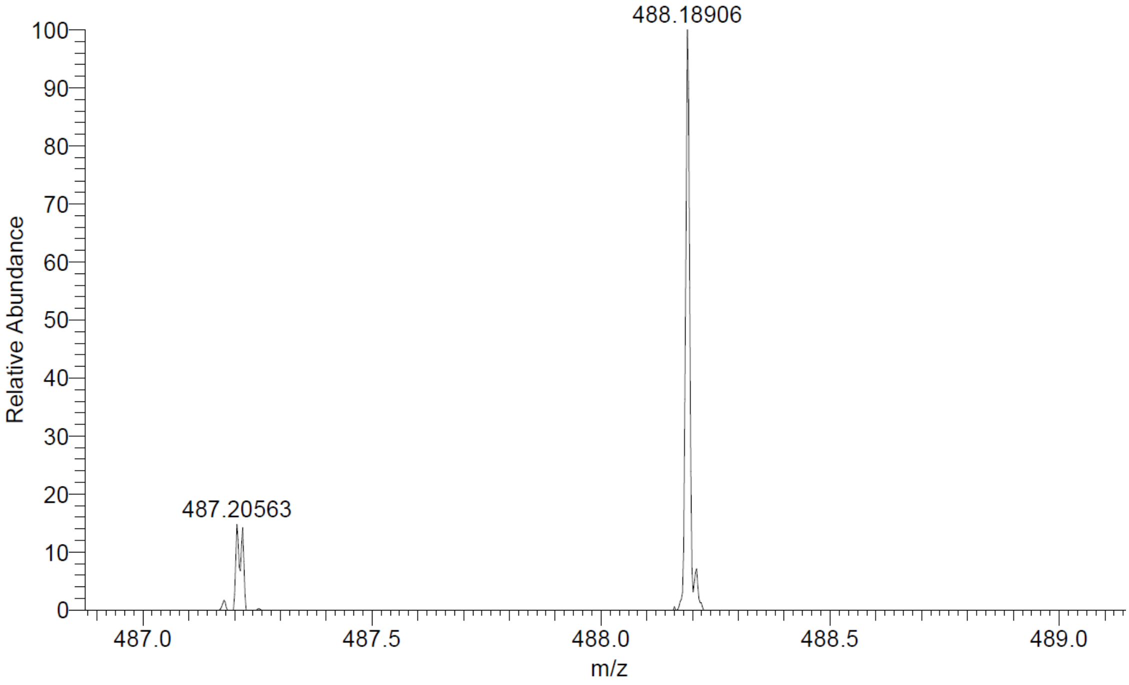

ESI-MS of compound **4c**

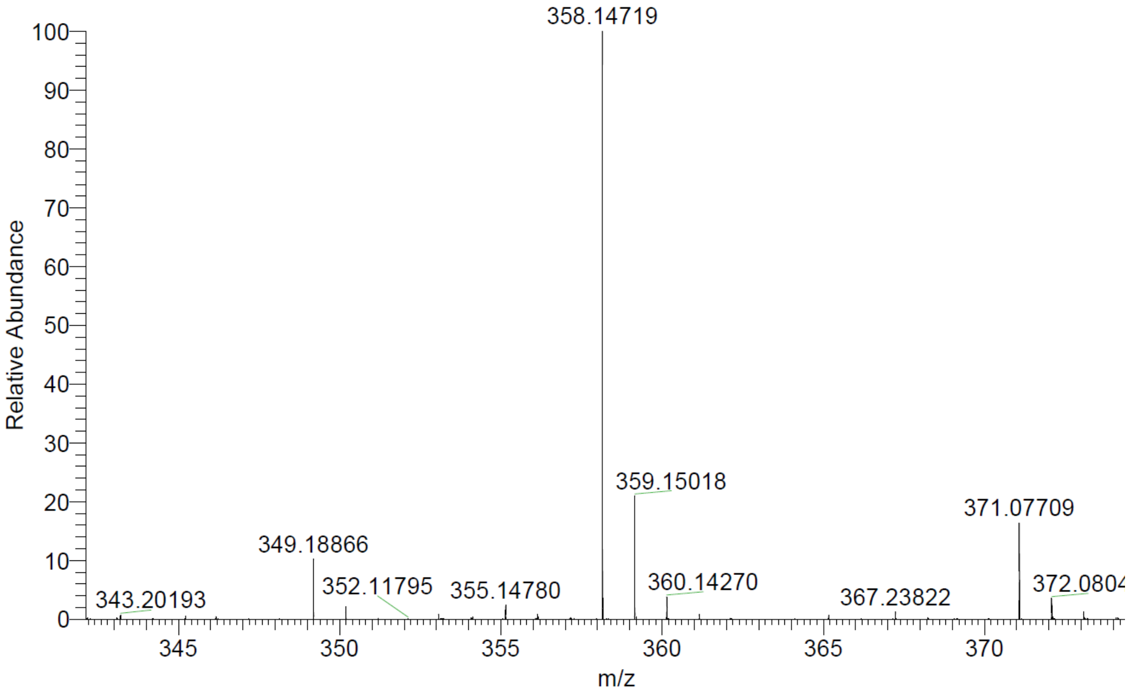

ESI-MS of compound **4d**

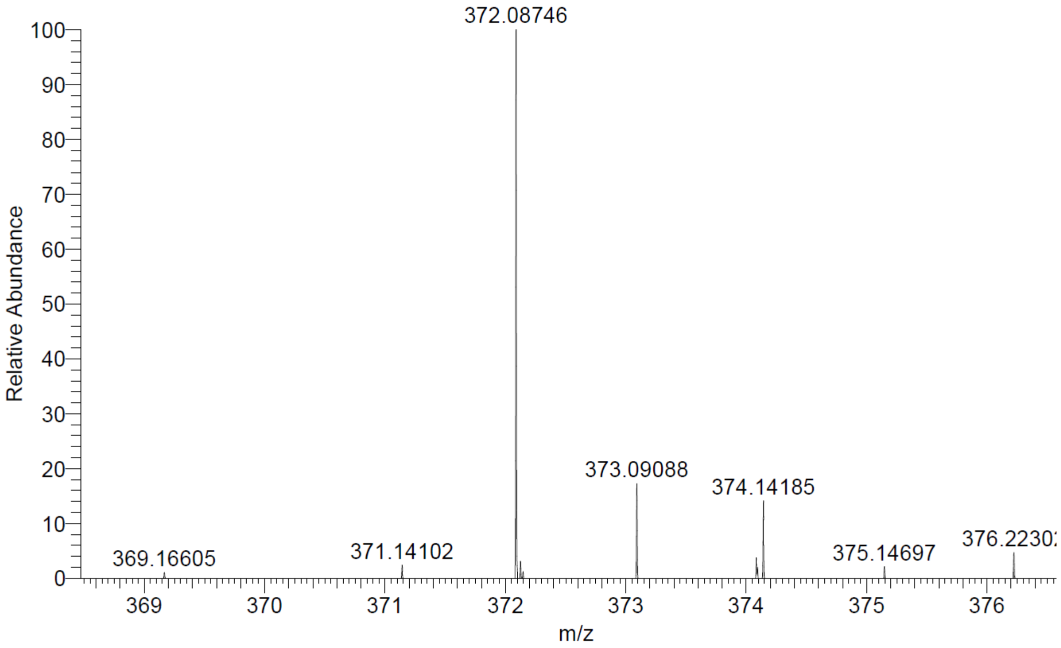

ESI-MS of compound **4e**

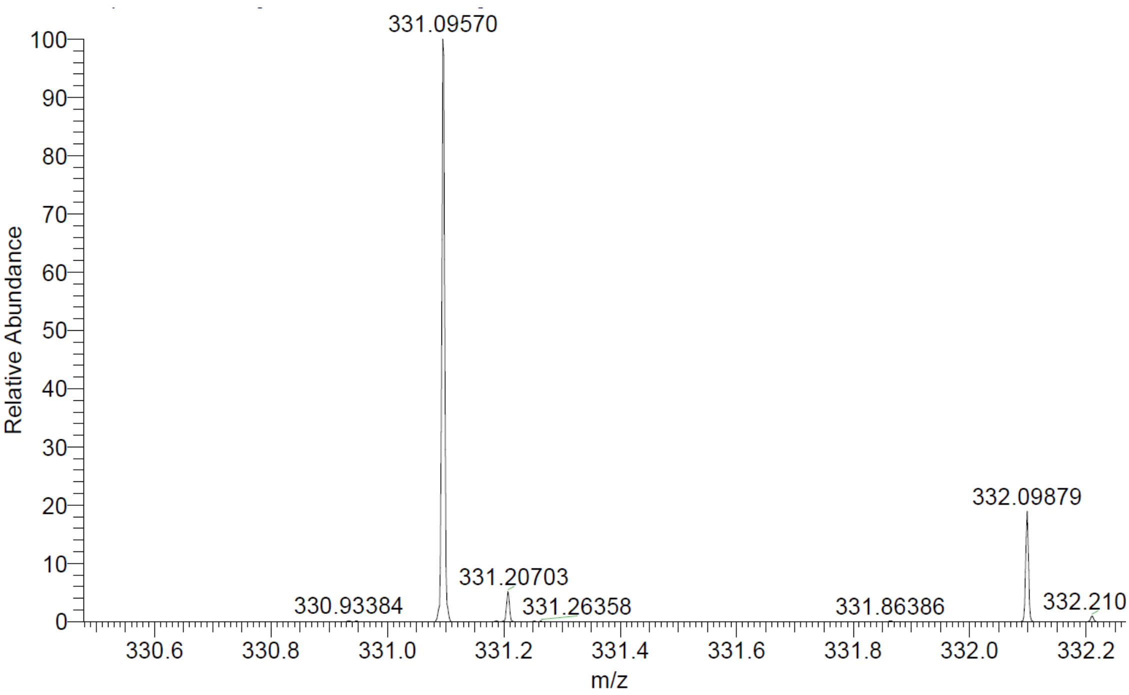

ESI-MS of compound **5**

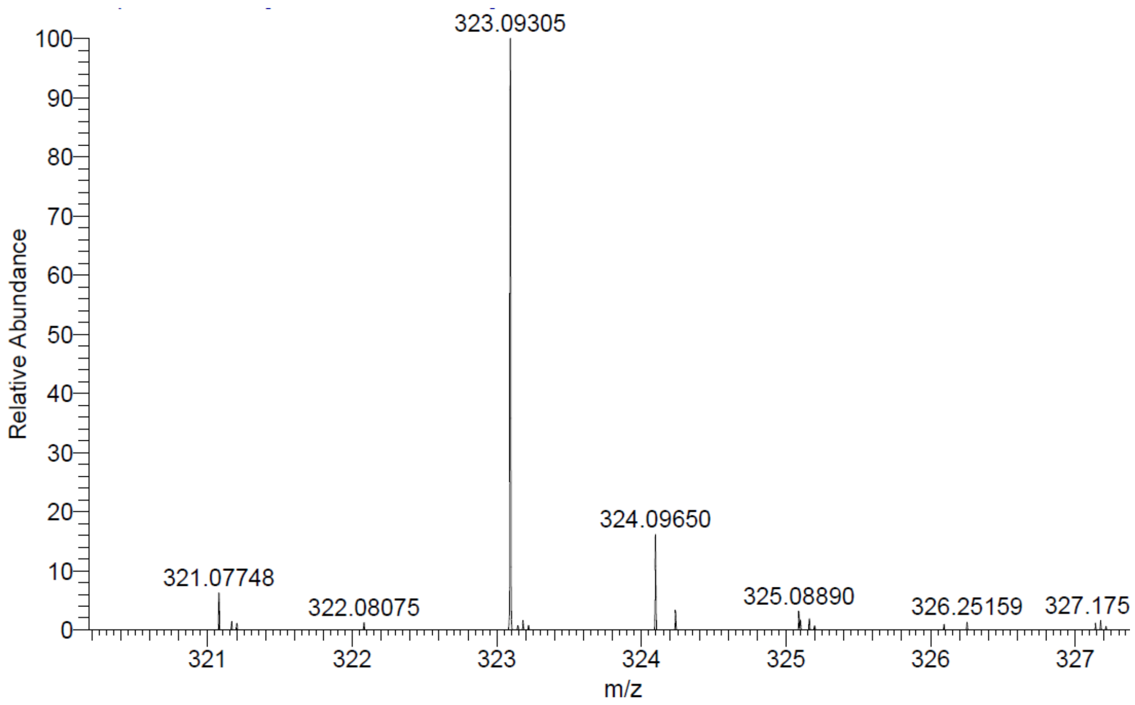

ESI-MS of compound **6**

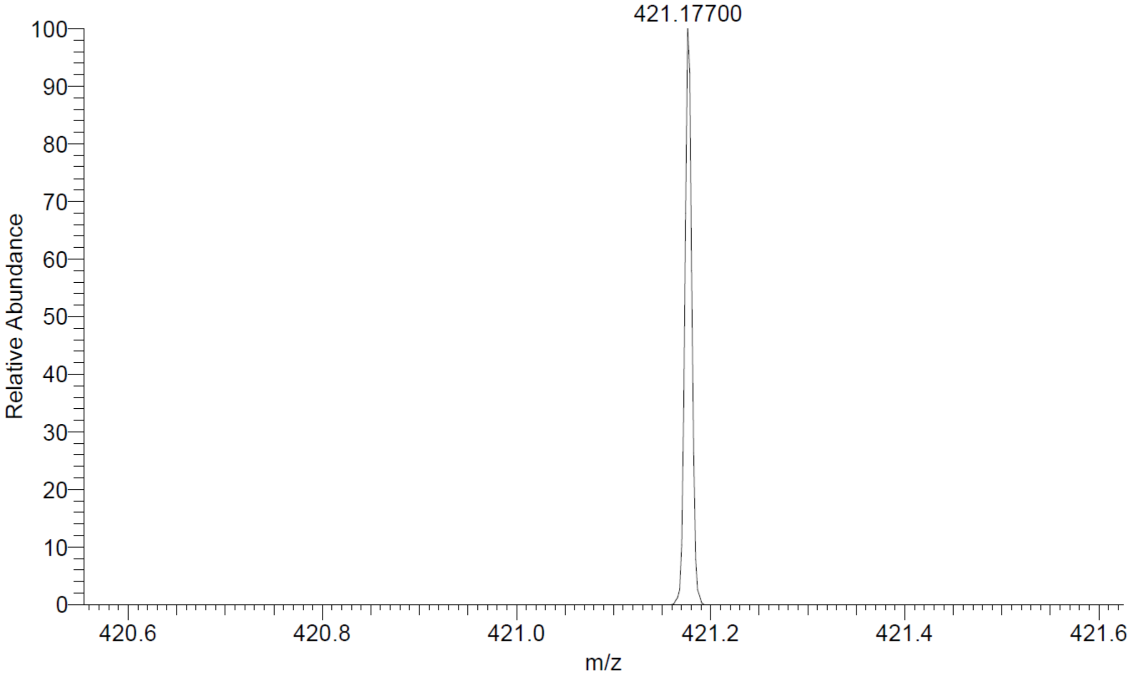

ESI-MS of compound **7**

References

1. Simeonov, S. P.; Coelho, J. A. S.; Afonso, C. A. M., *Org. Synth.* **2016,** *93*, 29.

2. Gomes, R. F. A.; Coelho, J. A. S.; Afonso, C. A. M., *ChemSusChem* **2019**, *12*, 420.

3. Zhao Y; Truhlar D. G.; *Theor. Chem. Acc.*, **2008**, *120*, 215.

4. Liu F.; Paton R. S.; Kim S.; Liang Y.; Houk K. N.; *J. Am. Chem. Soc.*, **2013**, *135*, 15642.

5. Liu F.; Liang Y.; Houk K. N.; *Acc. Chem. Res.*, **2017**, *50*, 2297.

6. (https://www.cylview.org). C. Y. Legault, CYLview, 1.0 b, Universit´e de Sherbrooke, Quebec Canada, 2009.

7. Gomes, P. A.; Cardoso M.; Dos Santos I. R.; Sousa F.; Conceição J. M.; Silva V.; Duarte D.; Pereira R.; Oliveira R.; Nogueira F.; Alves L. C.; Brayner F. A.; Santos A. C.; Pereira V.; Leite A. C.; *ChemMedChem*, **2020,** 15(22), 2164.
